# Large-scale mapping of cortical alterations in 22q11.2 deletion syndrome: Convergence with idiopathic psychosis and effects of deletion size

**DOI:** 10.1038/s41380-018-0078-5

**Published:** 2018-06-13

**Authors:** Daqiang Sun, Christopher R. K. Ching, Amy Lin, Jennifer K. Forsyth, Leila Kushan, Ariana Vajdi, Maria Jalbrzikowski, Laura Hansen, Julio E. Villalon-Reina, Xiaoping Qu, Rachel K. Jonas, Therese van Amelsvoort, Geor Bakker, Wendy R. Kates, Kevin M. Antshel, Wanda Fremont, Linda E. Campbell, Kathryn L. McCabe, Eileen Daly, Maria Gudbrandsen, Clodagh M. Murphy, Declan Murphy, Michael Craig, Jacob Vorstman, Ania Fiksinski, Sanne Koops, Kosha Ruparel, David R. Roalf, Raquel E. Gur, J. Eric Schmitt, Tony J. Simon, Naomi J. Goodrich-Hunsaker, Courtney A. Durdle, Anne S. Bassett, Eva W. C. Chow, Nancy J. Butcher, Fidel Vila-Rodriguez, Joanne Doherty, Adam Cunningham, Marianne B.M. van den Bree, David E. J. Linden, Hayley Moss, Michael J. Owen, Kieran C. Murphy, Donna M. McDonald-McGinn, Beverly Emanuel, Theo G. M. van Erp, Jessica A. Turner, Paul M. Thompson, Carrie E. Bearden

**Affiliations:** 1 Department of Psychiatry and Biobehavioral Sciences, Semel Institute for Neuroscience and Human Behavior, University of California, Los Angeles, Los Angeles, CA USA; 2 Department of Mental Health, Veterans Affairs Greater Los Angeles Healthcare System, Los Angeles, CA USA; 3 Imaging Genetics Center, Mark and Mary Stevens Neuroimaging & Informatics Institute, Keck School of Medicine, University of Southern California, Marina del Rey, CA USA; 4 Interdepartmental Neuroscience Program, University of California, Los Angeles, Los Angeles, CA USA; 5 Department of Psychology, University of California, Los Angeles, Los Angeles, CA USA; 6 Department of Psychiatry, University of Pittsburgh, Pittsburgh, PA USA; 7 Department of Psychiatry & Neuropsychology, Maastricht University, Maastricht, Netherlands; 8 Department of Psychiatry and Behavioral Sciences, State University of New York, Upstate Medical University, Syracuse, NY USA; 9 Department of Psychology, Syracuse University, Syracuse, NY USA; 10 PRC GrowUpWell, University of Newcastle, Newcastle, Australia; 11 School of Psychology, University of Newcastle, Newcastle, Australia; 12 Department of Radiology, Division of Neuroradiology, Perelman School of Medicine, University of Pennsylvania, Philadelphia, PA USA; 13 Sackler Institute for Translational Neurodevelopment and Department of Forensic and Neurodevelopmental Sciences, King’s College London, Institute of Psychiatry, Psychology & Neuroscience, London, UK; 14 Behavioural Genetics Clinic, Adult Autism Service, Behavioural and Developmental Psychiatry Clinical Academic Group, South London and Maudsley Foundation NHS Trust, London, UK; 15 National Autism Unit, Bethlem Royal Hospital, Behavioural and Developmental Psychiatry Clinical Academic Group, South London and Maudsley Foundation NHS Trust, London, UK; 16 Hospital for Sick Children, Toronto, ON Canada; 17 Department of Psychiatry, Brain Center Rudolf Magnus, University Medical Center Utrecht, Utrecht, The Netherlands; 18 Clinical Genetics Research Program, Centre for Addiction and Mental Health, Toronto, Ontario Canada; 19 Department of Psychiatry, University of Toronto, Toronto, ON Canada; 20 The Dalglish Family 22q Clinic, Department of Psychiatry, and Toronto General Research Institute, University Health Network, Toronto, ON Canada; 21 Campbell Family Mental Health Research Institute, Centre for Addiction and Mental Health, Toronto, ON Canada; 22 Department of Psychiatry, University of Pennsylvania, and the Lifespan Brain Institute, Penn Medicine and the Children’s Hospital of Philadelphia, Philadelphia, PA USA; 23 UC Davis MIND Institute and Department of Psychiatry and Behavioral Sciences, Davis, CA USA; 24 Department of Psychology, Brigham Young University, Provo, UT USA; 25 Department of Psychiatry, University of British Columbia, Vancouver, British Columbia Canada; 26 MRC Centre for Neuropsychiatric Genetics and Genomics, Division of Psychological Medicine and Clinical Neurosciences, Cardiff University, Cardiff, UK; 27 Department of Psychiatry, Royal College of Surgeons in Ireland, Dublin, Ireland; 28 Division of Human Genetics, The Children’s Hospital of Philadelphia, Philadelphia, Pennsylvania USA; 29 Department of Pediatrics, The Perelman School of Medicine at the University of Pennsylvania, Philadelphia, Pennsylvania USA; 30 Division of Clinical Genetics, The Children’s Hospital of Philadelphia, Philadelphia, Pennsylvania USA; 31 Department of Psychiatry and Human Behavior, University of California, Irvine, Irvine, CA USA; 32 Imaging Genetics and Neuroinformatics Lab, Department of Psychology, Georgia State University, Atlanta, GA USA; 33 Mind Research Network, Albuquerque, NM USA; 34 Departments of Neurology, Psychiatry, Radiology, Engineering, Pediatrics and Ophthalmology, University of Southern California, California, CA USA

**Keywords:** Neuroscience, Schizophrenia

## Abstract

The 22q11.2 deletion (22q11DS) is a common chromosomal microdeletion and a potent risk factor for psychotic illness. Prior studies reported widespread cortical changes in 22q11DS, but were generally underpowered to characterize neuroanatomic abnormalities associated with psychosis in 22q11DS, and/or neuroanatomic effects of variability in deletion size. To address these issues, we developed the ENIGMA (Enhancing Neuro Imaging Genetics Through Meta-Analysis) 22q11.2 Working Group, representing the largest analysis of brain structural alterations in 22q11DS to date. The imaging data were collected from 10 centers worldwide, including 474 subjects with 22q11DS (age = 18.2 ± 8.6; 46.9% female) and 315 typically developing, matched controls (age = 18.0 ± 9.2; 45.9% female). Compared to controls, 22q11DS individuals showed thicker cortical gray matter overall (left/right hemispheres: Cohen’s *d* = 0.61/0.65), but focal thickness reduction in temporal and cingulate cortex. Cortical surface area (SA), however, showed pervasive reductions in 22q11DS (left/right hemispheres: *d* = −1.01/−1.02). 22q11DS cases vs. controls were classified with 93.8% accuracy based on these neuroanatomic patterns. Comparison of 22q11DS-psychosis to idiopathic schizophrenia (ENIGMA-Schizophrenia Working Group) revealed significant convergence of affected brain regions, particularly in fronto-temporal cortex. Finally, cortical SA was significantly greater in 22q11DS cases with smaller 1.5 Mb deletions, relative to those with typical 3 Mb deletions. We found a robust neuroanatomic signature of 22q11DS, and the first evidence that deletion size impacts brain structure. Psychotic illness in this highly penetrant deletion was associated with similar neuroanatomic abnormalities to idiopathic schizophrenia. These consistent cross-site findings highlight the homogeneity of this single genetic etiology, and support the suitability of 22q11DS as a biological model of schizophrenia.

## Introduction

Micro-deletions or duplications of chromosomal regions (copy number variants; CNVs) are causally involved in a range of developmental brain disorders [1]. One such recurrent CNV is a deletion in the 22q11.2 region, typically encompassing ~50 protein-coding genes [2], which causes the 22q11.2 deletion syndrome (22q11DS; OMIM #188400, #192430). 22q11DS is one of the most penetrant genetic risk factors for psychotic illness [3], increasing risk around 30-fold relative to the general population [4–6]. 22q11DS is also associated with varied phenotypic expression, including cardiac defects, craniofacial anomalies, and intellectual disability [2, 7]. Given its known, relatively homogeneous genetic etiology, investigation of this microdeletion offers a unique opportunity to identify early neural biomarkers of psychosis.

Neuroanatomic alterations in 22q11DS have been investigated in several single-site studies. Early magnetic resonance imaging (MRI) studies reported whole-brain volumetric reductions in 22q11DS, particularly in midline cortical regions [8–11]. A rostro-caudal gradient of volumetric reduction was also reported, with greatest reduction in occipital lobes, while frontal regions were relatively preserved [12]. More recent studies have parcellated the cerebral cortex in detail, investigating measures of cortical thickness and surface area [13, 14], which may have distinct genetic and neurobiological origins [15, 16]. Some studies noted increases in cortical thickness in 22q11DS relative to controls, with focal thinning in the superior temporal gyrus and cingulate cortex, along with global reductions in surface area [8, 13, 14, 17–19]. It is not clear, however, if these patterns are universally found in 22q11DS. Moreover, in other neurogenetic conditions larger deletions are associated with greater phenotypic severity [20]; yet, to our knowledge, no prior studies have investigated the neuroanatomic effects of variations in 22q11.2 deletion size.

Determining the neural substrates of psychotic illness in 22q11DS has been a major focus of investigation. Meta-analyses of structural MRI studies of patients with idiopathic schizophrenia report lower volumes in frontal and temporal regions [21–24], including the anterior cingulate and insula [25, 26]. Some evidence suggests that neuroanatomic regions typically disrupted in idiopathic schizophrenia are also linked to psychosis in 22q11DS. Lower frontal and superior temporal gyrus (STG) gray matter volumes were observed in adults with 22q11DS and a schizophrenia diagnosis, relative to 22q11DS adults without schizophrenia [19, 27, 28]. Kates et al. [29] also found that progressive volumetric decreases in STG predicted later psychotic symptoms in 22q11DS youth, and lower cingulate gyrus volume was associated with more severe psychotic symptoms [30]. These initial studies support overlap between neuroanatomic abnormalities in idiopathic schizophrenia and 22q11DS-associated psychosis; however, confirmation in a large-scale study and systematic comparison of regional changes between psychosis in 22q11DS and idiopathic schizophrenia are needed.

To address these questions, researchers worldwide studying cohorts of 22q11.2 deletion carriers formed the 22q11.2 Working Group as part of the Enhancing NeuroImaging Genetics through Meta-Analysis (ENIGMA) Consortium [31–33] (http://enigma.ini.usc.edu). With the goal of data harmonization across sites, this consortium effort represents the largest-ever analysis of brain structural alterations in 22q11DS. We addressed the following research questions:

1. Is there a distinct neuroanatomic signature of 22q11DS?
2. Do cortical metrics differ between 22q11DS individuals with and without psychosis? Do these neuroanatomic patterns overlap with those of idiopathic schizophrenia?
3. Does the size of the 22q11.2 deletion affect the magnitude of cortical alterations?

## Methods

### Participants

Seven-hundred and eighty-nine individuals – 474 22q11DS subjects and 315 typically developing controls - from 10 study sites in the ENIGMA 22q11DS working group were included in the analyses. These individuals were selected from a larger pool of 944 participants, after excluding related individuals (*N* = 79) and individuals with poor quality MRI scans (*N* = 65) or extreme brain measures (*N* = 11). Study inclusion/exclusion criteria and measures are detailed for each sample in Supplementary Table S1. Institutional review boards at participating institutions approved all study procedures. Written informed consent/assent was obtained from all study participants.

### Image acquisition

Thirteen sets of T1-weighted MRI anatomical brain scans were acquired from 10 participating sites; acquisition parameters are detailed in Supplementary Table S2. The imaging data from UCLA, UC Davis and University of Toronto were each acquired on two different scanners, and were therefore treated as independent data sets in the analyses.

### Image processing

De-identified scans from each site were transferred to secure UCLA servers; image processing and analyses were conducted on secure USC Laboratory of Neuro Imaging (LONI) servers. Scans were processed using FreeSurfer (version 5.3.0) [34]. Quality control was performed for each scan, including visual inspection and the use of standardized ENIGMA quality control procedures (http://enigma.ini.usc.edu/protocols/imaging-protocols) [35, 36]. Applying FreeSurfer’s reconstruction pipeline, local cortical thickness (CT) and surface area (SA) were calculated on each vertex of reconstructed hemispheric surface model [37], and statistical analyses were conducted on each vertex. Measures of CT and SA were also obtained from 68 cortical regions (34 per hemisphere), based on the Desikan–Killiany atlas [38], and these regional measures were used to identify appropriate modeling for the above-mentioned surface-based analyses (see Supplementary Methods for details).

### Statistical analyses

#### 22q11DS vs. control differences

This comparison included 701 individuals (22q11DS *n* = 386; control *n* = 315) from 11 data sets involving 9 study sites; subjects from 2 sites (Toronto and Utrecht) that had no healthy control data were excluded from this analysis.

Group differences in CT and SA were examined using general linear models (GLM), with each brain measure as the dependent variable and group as the independent variable, adjusted for data set/site, sex, and age. Based on data visualization and model comparisons (Supplementary Figure S1a,b; Supplementary Tables S3a,b), age effects were modeled linearly for SA, while a quadratic term was included in the model for CT. Interactions between group, sex, and age were largely non-significant (Supplementary Tables S4a,b; S5a,b**)** and therefore not included in the models. Because intracranial volume (ICV) was significantly correlated with global SA but not CT (Supplementary Figure S2), ICV was included as a covariate only for SA comparisons, in all analyses. Treating data set as a random variable, mixed linear models were also used for comparison (see Supplemental Methods). Cohen’s *d* effect size estimates were derived from *t*-values for the group differences [39]. For all significance tests, the False Discovery Rate (FDR) [40] with q-value at 0.05 was applied to control false positive errors due to multiple comparisons. FDR-corrected *p*-values below 0.05 were considered significant. All surface-based analyses were conducted using FreeSurfer’s *mri_glmfit*. Tests for individual cortical regions were performed in the R statistical environment [41].

#### 22q11DS vs. control classification analysis

To examine how accurately 22q11DS subjects can be differentiated from controls based on cortical measures, a machine-learning based classification analysis was conducted on the regional CT and SA values from the same data sets described above using Glmnet. Glmnet uses an L1-norm regularization to fit a generalized linear model. It implements built-in feature selection, and is robust when predictors are highly correlated [42]. The Caret package [43] in R was used to facilitate training and testing. Specifically, the whole data set was randomly divided into training sets and testing sets 20 times at a ratio of 3:1. For each division, 10-fold cross-validations were conducted on the training set to achieve an optimized model, which was then applied to testing data to evaluate classification accuracy. Sensitivity, specificity, and accuracy of group prediction were averaged over the 20 divisions.

To further test the reliability of prediction, brain scans from the two sites with only 22q11DS cases (*n* = 88) were used as an independent validation data set, to which the model trained from the above-mentioned data was applied.

#### Effects of psychosis on brain structure

To compare cortical measures between 22q11DS subjects with (22q11DS + Psychosis) and without psychosis (22q11DS-No-Psychosis), each 22q11DS + Psychosis subject was matched to a 22q11DS-No-Psychosis subject at the same site, with the same sex, and closest age. Psychosis diagnosis was determined by structured clinical interview at each site; for a subset, diagnoses were validated across sites via a consensus procedure [44] (see Supplementary Methods, Table S1). Group comparisons were conducted using GLM controlling for site, sex, and age. No group x age interactions were significant, and thus were not included in statistical models. Based on statistical model comparisons, age was treated as a linear term for both CT and SA (Supplementary Tables S6a,b). As in the above analyses, ICV was adjusted for SA comparisons. Secondly, we conducted a classification analysis using the same Glmnet algorithm described above, in order to determine whether we could accurately distinguish 22q11DS cases with psychosis from those without, based on neuroanatomic patterns.

#### Pattern similarity in cortical measures between 22q11DS with psychosis and idiopathic schizophrenia

To further clarify if psychosis-related brain alterations in 22q11DS resemble the pattern observed in idiopathic schizophrenia, we correlated the effect sizes (Cohen’s *d*) for cortical measures from the comparison of psychotic vs. non-psychotic 22q11DS subjects with those from the ENIGMA schizophrenia working group, the largest meta-analysis of structural brain alterations in schizophrenia (4474 patients with idiopathic schizophrenia; 5098 healthy controls) [45]. To investigate the specificity of the above correlation, a parallel analysis was conducted for psychotic vs. non-psychotic 22q11DS compared to major depressive disorder (MDD; *N* = 1902) vs. healthy controls (*N* = 7658) from the ENIGMA MDD Working Group [35].

#### Proximal nested (A–B) vs typical (A–D) 22q11.2 deletions

The combined data sets of 22q11DS subjects included microdeletions of variable size; the large sample size allowed comparison of anatomical effects of the two most frequent 22q11.2 deletion types, the typical ~3 Megabase (Mb) A–D deletion (present in ~85% of cases) and the smaller, nested ~1.5 Mb A–B deletion (present in ~10% of cases) [2]. Deletion size was determined using multiplex ligation-dependent probe amplification (MLPA) [46]. Each subject with an A–B deletion was matched with 4–5 subjects with A–D deletions, and 4–5 healthy controls, based on same site and sex, and closest age. The three groups were compared in an analysis of covariance (ANCOVA) model, controlling for site, sex, and age, with post-hoc pairwise contrasts between each group. Age^2^ [2] was modeled for CT, as significant quadratic age effects were observed; however, age was modeled linearly for SA, as no significant nonlinear effects were observed (Supplementary Tables S7a,b). Again, ICV was covaried in SA comparisons.

#### Medication, Handedness and IQ Effects

Secondary analyses addressed effects of medication usage, handedness and IQ on cortical structure (Supplementary Methods, Table S8**)**.

## Results

### 22q11DS vs. control differences

There were no differences in sex or age between 22q11DS subjects and controls, either within each site, or when all sites were combined (Table 1). However, a greater proportion of controls were right-handed and, as expected, controls had significantly higher IQ than 22q11DS cases. As such, these variables were examined in secondary analyses, as noted above.

**Table 1:** Participant Demographics, By Site

	Control						22q11DS					
Site	*N*	*N* (%) female^a^	Mean age^b^	Age SD	*N* (%) right handed^c^	IQ (SD)^d^	*N*	*N* (%) female	Mean age	Age SD	*N* (%) right handed	IQ (SD)
Cardiff	11	6 (54.5)	14.5	1.6	6 (55%)	106.7 (7.5)	15	8 (53.3)	15.1	2.9	12 (67%)	77.4 (19.1)
London IoP	25	15 (60)	19.6	6	24 (96%)	115.9 (10.3)	37	17 (45.9)	16.6	7.2	28 (75.7%)	84.7 (12.9)
Maastricht +	45	15 (33.3)	29.3	9.6	6 (85.7%)	106.4 (15.4)	27	13 (48.1)	29.2	6.7	13 (76.5%)	74.5 (11.3)
Newcastle	26	14 (53.8)	16.8	3.3	19 (79%)	108.7 (15.7)	19	12 (63.2)	17.2	2.9	17 (89.5%)	73.7 (13.6)
U Penn	48	20 (41.7)	17.5	3.2	39 (81.3%)	NA++	49	19 (38.8)	17.3	3.2	38 (86.4%)	76.3 (18.6)
SUNY Upstate	20	8 (40)	20.5	1.2	17 (85%)	105.2 (14.1)	54	24 (44.4)	20.7	2.3	37 (78.7%)	73.7 (11.3)
Toronto1	12	4 (33.3)	42.4	8.7	9 (75%)	—	15	7 (46.7)	41.5	7.6	12 (80%)	72.5 (10.9)
Toronto2	0	NA	NA	NA	—	—	35	22 (62.9)	27.8	10.2	28 (80%)	72.2 (10.1)
UCDavis1	38	19 (50)	10.4	2.5	32 (86%)	114.4 (11.0)	40	17 (42.5)	10.7	2.1	29 (72.5%)	72.7(13.3)
UCDavis2	48	23 (47.9)	10.8	2.4	45 (93.8%)	113.7 (14.6)	64	30 (46.9)	11.3	2.5	53 (82.8%)	75.5 (13.2)
UCLA1	23	16 (69/6)	14.3	5.7	22 (95.7%)	118.1 (20.5)	28	14 (50)	14.4	6.7	22 (84.6%)	80.7 (14.6)
UCLA2	19	5 (26.3)	13.3	3.6	13 (72.2%)	104.7 (17.8)	38	21 (55.3)	15.8	7.9	28 (73%)	77.6 (12.2)
Utrecht	0	NA	NA	NA	—	—	53	38 (71.7)	17.6	4.2	38 (74.5%)	69.7 (8.5)
Total	315	145 (46)	18	9.2	232 (85%)	111.6** (15.1)	474	242 (51.1)	18.2	8.6	333 (74.7%)	75.2 (13.2)

+ Maastricht- IQ missing for 13 controls [other controls received Dutch National Adult Reading Test (NART)]; handedness based on 7 controls and 17 22q11DS cases; + + At UPenn, controls received Penn-CNB and WRAT as proxy measures of IQ (Supplementary Table S1)

^a^*Χ*^2^ = 1.9, *p* = .17; Cramer’s *V* = .049; b. *F* = .094; *p* = .76; *d* = .00; c. *Χ*^2^ = 11.25; *p* = .004; Cramer’s *V* = 0.13; d. *F* = 1052.43; *p* < .001; *d* = 2.5

**Fig. 1:**
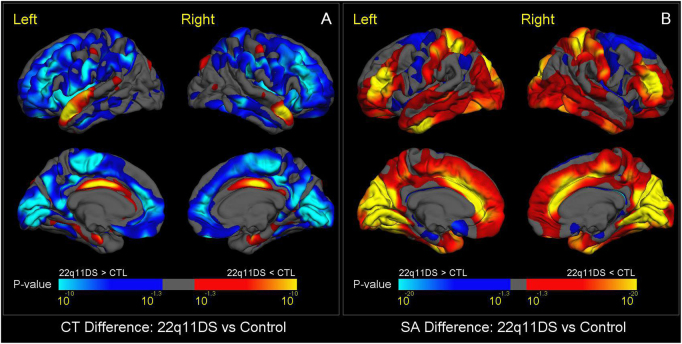
Vertex-Wise Mapping of Difference in CT and SA between 22q11DS and Healthy Control Subjects. **a** shows vertex-wise differences in CT, and **b** shows vertex-wise differences in SA. Colored areas show *p*-values for group differences after FDR correction (*q* = 0.05) for all vertices across both left and right cortical surfaces. Blue colors represent significant increases in 22q11DS subjects compared to healthy controls, whereas red-yellow colors represent significant reductions in 22q11DS subjects. Compared to controls, subjects with 22q11DS showed greater CT and smaller SA, most prominently in the posterior medial cortex including bilateral cuneus, precuneus, lingual gyrus, pericalcarine cortex, and bilateral medial and lateral frontal cortex. Subjects with 22q11DS showed a distinctive reduction of both CT and SA in bilateral cingulate cortex. They also had reduced  CT in the superior temporal gyrus, and greater SA in the superior parietal cortex and rostral middle frontal gyrus.

Overall brain metrics were highly consistent across sites (Supplementary Figure S3 and Table S9). We found widespread reductions in SA, along with globally thicker cortical gray matter in 22q11DS subjects relative to controls. The spatial pattern of thicker cortex in 22q11DS resembled that of SA reduction, with the exception of thicker cortex in bilateral insula, and thinner cortex relative to controls in bilateral parahippocampal and superior temporal gyri, and left caudal anterior cingulate cortex (Fig. 1a). The most prominent SA reductions were found bilaterally in the medial occipital and anterior cingulate cortex; superior parietal cortex and rostral middle frontal gyrus were among the lateral regions showing significantly smaller SA in 22q11DS vs. controls (Fig. 1b). Effect sizes and *p*-values of regional CT and SA differences are shown in Supplementary Tables S10a,b, respectively, indicating that effect sizes for reduced SA in 22q11DS vs. controls were roughly twice the size of the effects for increased CT. Scatterplots displaying these results are in Supplementary Figures S4a,b. The overall pattern of findings remained the same when mixed-effects models were used [Supplementary Table S11a,b].

### 22q11DS vs. control classification

An average classification accuracy of 93.8% (*p* = 4.46 × 10^−26^, sensitivity 94.2%; specificity 93.3%) was achieved across 20 runs (Supplementary Table S12a**)**. The top five contributors to the overall accuracy of the model were SA in the left caudal anterior cingulate, precentral gyrus, and bilateral cuneus, and CT in the left insula (Supplementary Table S12b). When the classifier derived from the 11 data sets with cases and controls was applied to the 2 data sets with only 22q11DS cases, a classification sensitivity of 100% was achieved (i.e., all were classified as cases).

### Effects of psychosis on brain structure

The matched groups of 22q11DS + Psychosis (*n* = 60) and 22q11DS-No-Psychosis (*n* = 60) were similar in demographic characteristics, although those with psychosis had significantly lower IQ and increased proportion of antipsychotic medication usage, as expected (Supplementary Table S13a,b).

**Fig. 2:**
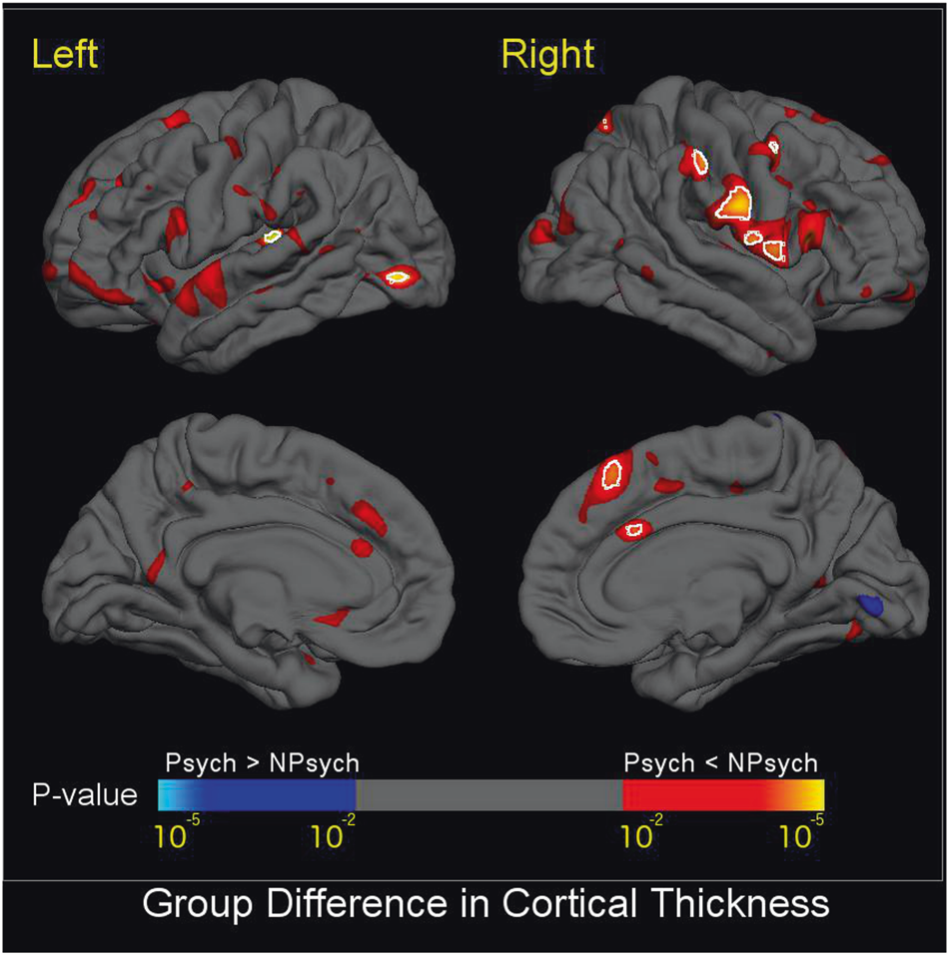
Mapping of CT Differences between 22q11DS + Psychosis and 22q11DS-No-Psychosis. Colored areas show *p*-values of group difference, and white circles include regions that pass FDR correction at *q* = 0.05. Blue colors represent thicker cortical gray matter in 22q11DS+ Psychosis compared to 22q11DS-No-Psychosis, and red-yellow colors represent thinner cortical gray matter in 22q11DS+ Psychosis vs. 22q11DS-No-Psychosis. Compared to those without psychosis, 22q11DS subjects with psychosis showed significantly thinner cortex in the left superior temporal gyrus and lateral occipital cortex, and right medial superior frontal, cingulate, pre- and post-central, and supramarginal gyri

Relative to the 22q11DS-No-Psychosis group, the 22q11DS + Psychosis group showed significantly thinner cortex in the left superior temporal gyrus and lateral occipital cortex, and right medial superior frontal, cingulate, pre- and post-central, and supramarginal gyri (Fig. 2). No significant differences in SA were found between 22q11DS subjects with and without psychosis. Effect sizes and *p*-values for cortical regions are presented in Supplementary Table S14a,b, which showed significant CT differences across several frontal and temporal regions. Overall, the cortex was significantly thinner in 22q11DS + Psychosis, with similar, moderate effect sizes in the right and left hemispheres (*d* = −0.63 and −0.58, respectively; see Supplementary Figures S5a, b for scatterplots of regional differences in CT and SA). However, the overall classification of the two groups was not significant (accuracy 61.2%, *p* = 0.19), when the same Glmnet procedure detailed above was applied.

### Pattern similarity in cortical measures between 22q11DS + Psychosis and idiopathic schizophrenia

**Fig. 3:**
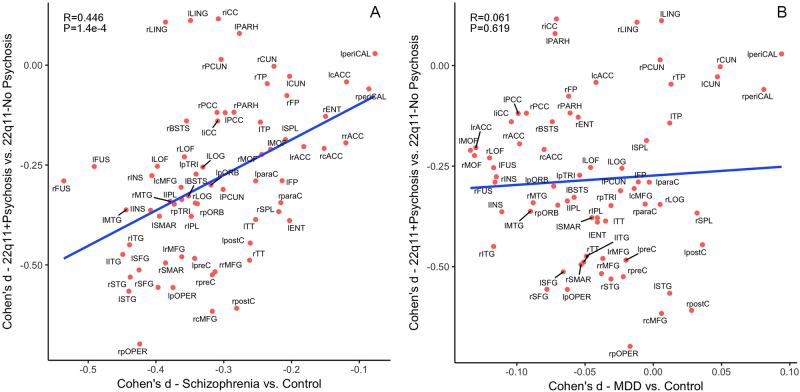
Pattern similarity in CT deficits between 22q11DS with psychosis and idiopathic schizophrenia, in contrast to major depressive disorder (MDD). Here we correlated the effect sizes (Cohen’s *d*) for regional CT measures from the comparison between 22q11DS + Psychosis and 22q11DS-No-Psychosis groups with those from the ENIGMA Schizophrenia working group [47], in contrast to the ENIGMA MDD study [35]. **a** Correlation in the effect sizes of CT deficits between idiopathic schizophrenia and 22q11 + Psychosis; **b** Correlation in the effect sizes of CT deficits between MDD and 22q11 + Psychosis. The effect sizes for CT deficits from psychotic vs. non-psychotic 22q11DS comparisons were significantly correlated with those in ENIGMA idiopathic schizophrenia vs. control comparisons. In contrast, the same effect sizes were not significantly correlated with those in ENIGMA MDD vs. control comparisons. Both *x*- and *y*-axes represent effect sizes in Cohen’s *d* in the above-mentioned comparison for all 68 cortical regions derived from the FreeSurfer cortical parcellation. Abbreviations of the cortical regions are adopted from the brainGraph package [67] as follows: BSTS banks of superior temporal sulcus, cACC caudal anterior cingulate cortex, *cMFG* caudal middle frontal gyrus, *CUN* cuneus, *ENT* entorhinal cortex, *FUS* fusiform gyrus, *IPL* inferior parietal cortex, *ITG* inferior temporal gyrus, *iCC* isthmus cingulate cortex, *LOG* lateral occipital cortex, *LOF* lateral orbitofrontal cortex, *LING* lingual gyrus, MOF medial orbitofrontal cortex, *MTG* middle temporal gyrus, PARH parahippocampal gyrus, paraC paracentral, lobule, *pOPER* pars opercularis of inferior frontal gyrus, *pORB* pars orbitalis of inferior frontal gyrus, *pTRI* pars, triangularis of inferior frontal gyrus, *periCAL* pericalcarine cortex, *postC* post-central gyrus, *PCC* posterior, cingulate cortex, *preC* precentral gyrus, *PCUN* precuneus, *rACC* rostral anterior cingulate corte, *rMFG* rostral, middle frontal gyrus, *SFG* superior frontal gyrus, *SPL* superior parietal cortex, *STG* superior temporal gyrus, *SMAR* supramarginal gyrus, *FP* frontal pole, *TP* temporal pole, *TT* transverse temporal gyrus, *INS* insula. *L* left, *R* right

Effect sizes in Cohen’s *d* for regional CT deficits in 22q11DS + Psychosis versus 22q11DS-No-Psychosis were significantly correlated with those in the ENIGMA idiopathic schizophrenia vs. control comparisons (*r* = 0.446, *p* = 1.4 × 10^−4^). In contrast, the same effect sizes were not correlated with those in the ENIGMA MDD vs. control comparisons (*r* = 0.061, *p* = 0.619). Scatterplots for the effect size correlations are shown in Fig. 3a,b.

### Proximal nested (A–B) vs typical (A–D) deletions vs. controls

After demographic matching, 23 22q11DS subjects with A–B deletions, 108 subjects with A–D deletions, and 87 control subjects were compared using ANCOVA (see demographics in Supplementary Tables S15a,b).

**Fig. 4:**
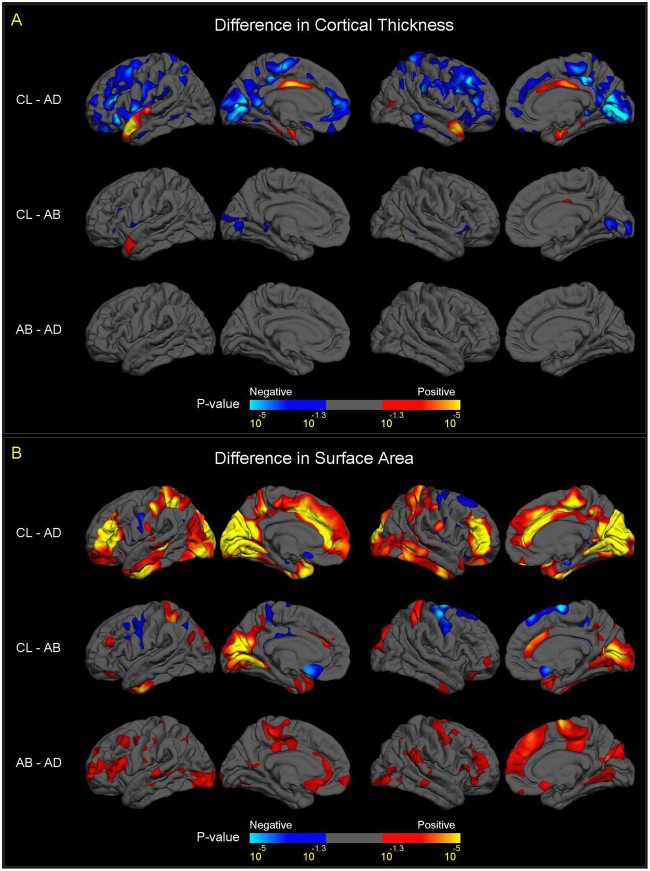
Vertex-wise mapping of differences in CT and SA, between A–B, A–D Deletion, and Control Subjects. For all figures, colored areas show *p*-values of group difference that remain significant after FDR correction (*q* = 0.05) for all vertices across both left and right cortical surfaces. The positive and negative directions in the color-bars indicate the signs of differences after subtracting one group from another labelled on the left side. **a** Differences in CT between A–B deletion, A–D deletion and control subjects. Compared to controls, subjects with A–B deletions showed thicker cortex (in blue colors) in bilateral pericalcarine cortex and bilateral inferior frontal gyrus, and thinner cortex (in red-yellow colors) in the left anterior superior temporal gyrus and right posterior cingulate cortex. Subjects with A–B deletions showed no significant difference in CT in any cortical region. The comparison of CT between subjects with A–D deletions and controls showed a similar pattern of group differences to the overall 22q11DS case-control analysis (Fig. 1a), although effects were diminished. **b** Difference in SA between A–B deletion, A–D deletion, and control subjects. Compared to controls, subjects with A–B deletions showed significantly reduced SA (in red-yellow colors), more prominent in the posterior portion of the medial and inferior cortical surface, including the bilateral cuneus, precuneus, pericalcarine, lingual, fusiform, and inferior temporal regions, and caudal anterior cingulate. Increased SA in A–B deletion cases vs. controls was observed in bilateral precentral, paracentral, and medial orbitofrontal regions (in blue colors). Compared to subjects with A–B deletions, subjects with A–D deletions showed widespread significant cortical SA reductions (in red-yellow colors), most prominently in the anterior portion of the medial cortical surface, including the paracentral lobules, cingulate, precentral, superior frontal regions, and widely distributed lateral cortical regions. Like CT, the comparisons of SA between subjects with A–D deletions and controls showed a similar pattern of group differences to the overall 22q11DS case-control analysis (Fig. 1b), although effects were diminished

The anatomical patterns of CT and SA differences between subjects with A–D deletions and controls (upper panels of Fig. 4a,b) resembled those in the overall case-control comparisons (Fig. 1).

Compared to healthy controls, subjects with A–B deletions showed significantly thinner cortex in the left anterior superior temporal gyrus and right posterior cingulate gyrus, and thicker cortex in bilateral pericalcarine and inferior frontal regions (Fig. 4a, middle panel). Subjects with A–B deletions also showed significantly reduced SA in bilateral medial occipital and cingulate cortex, as well as increased SA in sensorimotor cortex (Fig. 4b, middle panel).

Relative to those with smaller (A–B) deletions, 22q11DS subjects with A–D deletions showed highly significant, widespread reductions in cortical SA, most prominently in the anterior portion of the medial cortical surface and widely distributed lateral cortical regions (Fig. 4b, bottom panel). No differences in CT between A–B and A–D deletion cases surpassed FDR correction.

Scatterplots of regional differences in CT and SA between 22q11DS subjects with A–B vs. A–D deletions and vs. controls are shown in Supplementary Figures S6a,b, respectively; *t*-values and significance levels are presented in Supplementary Tables S16a,b. These results are very similar to the vertex-wise analysis results, indicating robust effects of deletion size on regional cortical SA, most prominently in frontal and parietal regions.

### Medication, handedness and IQ effects on cortical measures

Possible effects of medications on regional cortical measures in 22q11DS subjects were assessed using GLMs (see Supplementary Methods). No significant associations were detected between psychotropic medications at the time of MRI scan and either CT or SA (Supplementary Table S17a,b) in any cortical regions. Analysis within the 22q11DS + Psychosis group also showed minimal effects of antipsychotic medication on cortical measures (Table S18a,b). Similarly, effect sizes and significance levels for group differences were not substantively changed by covarying handedness (Supplementary Table S19a,b). Finally, patterns of group differences and significance levels largely remained unchanged when IQ was included a covariate, in overall case-control analyses (Supplementary Figures S7a,b) and when comparing 22q11DS cases with and without psychosis (Supplementary Figure S8).

## Discussion

This study, the largest neuroimaging investigation ever conducted of this well-characterized 22q11.2 deletion, revealed several key findings. First, compared to healthy controls, individuals with 22q11DS showed: (1) widespread thicker cortex bilaterally (left/right hemisphere *d* = 0.614/0.648), with the notable exception of thinner superior temporal, cingulate, and parahippocampal cortex and (2) widespread reductions in cortical SA; almost double the size of the effects observed for CT (left/right hemisphere *d* = −1.014/−1.021), with effects of greatest magnitude in parieto-occipital regions and the anterior cingulate. Secondly, 22q11DS subjects with psychosis showed significantly thinner cortex relative to those without a history of psychosis, with the strongest effects in fronto-temporal regions that are also most prominently affected in idiopathic psychosis [26, 47]^.^ Finally, we found for the first time that larger deletion size was associated with significantly reduced cortical SA.

The prominent reductions in posterior SA we observed in 22q11DS cases overall may explain the previously observed rostral-caudal gradient of volumetric deficits [12]. Further, the neuroanatomic signature of 22q11DS was so robust that cases could be classified with high accuracy. Our findings are consistent with imaging findings in the 22q11DS mouse model, indicating differentially lower SA in posterior brain regions with relative preservation of frontal regions [48, 49].

To our knowledge, this is the largest-ever comparison of demographically well-matched 22q11DS cases with and without psychotic disorder. Findings of thinner fronto-temporal cortex in 22q11DS + Psychosis align well with volumetric findings from prior, smaller studies [19, 28], but the enhanced power of this multisite study revealed a more extensive network of cortical regions. Effect sizes for the significant regional CT deficits were in the medium range (*d* = 0.45–0.70), similar to effect sizes for CT differences between idiopathic schizophrenia cases and healthy controls (*d* = −0.530/−0.516 for left/right hemisphere, respectively) [47]. Indeed, our cross-diagnosis correlational analysis indicated significant convergence with brain regions predominantly affected in idiopathic schizophrenia, which is supported by similar findings at the clinical phenotypic level [50, 51]. Furthermore, the divergence with neuroanatomic effects of MDD indicates specificity of the brain anatomic phenotype of 22q11DS + Psychosis.

Our study also provides the first evidence for phenotypic differences as a function of deletion size. Prior, small studies found no detectable effect of deletion size on phenotypic severity [52–54], but these studies were likely underpowered and, to our knowledge, none thus far investigated deletion size in relation to brain structure. Larger A–D deletions were associated with substantially reduced cortical SA, but not CT changes, compared to the smaller A–B deletions, suggesting specific effects of deletion size on cortical SA. Also of note, neuroanatomic differences between individuals with A–B deletions and controls showed a much narrower cortical distribution, restricted to pericalcarine regions, relative to typical (A–D) 22q11DS case vs. control differences.

Regarding developmental effects, we did not see much evidence for divergent trajectories of cortical development for 22q11DS cases overall, as few group x age interactions were significant. One prior longitudinal study observed delayed prefrontal thinning over a three-year follow-up period in adolescent patients with 22q11DS [55]. Given the wide age range of our sample, with fewer participants at the extreme ends of the age distribution, we may not have had sufficient power to detect interactions if they were present primarily in these developmental periods. These questions warrant further investigation in large, prospective longitudinal studies. In typical development, cortical thinning begins between ages 2 to 4 years and continues across the lifespan, whereas cortical SA follows a nonlinear maturational trajectory beginning in fetal development [56, 57], although it appeared largely linear within the age range investigated here. Increased progenitor cell production during embryonic development predominantly influences expansion of SA [58–61]; in contrast, CT depends on the neuronal output from each radial unit, and is thus considered a proxy for the number of cells in a column [58, 60]. As such, the observed pervasive SA decreases in 22q11DS may reflect reduced progenitor cell production in multiple cortical regions, implying that this distinctive phenotype in 22q11DS originates early in the course of brain development.

Currently, the precise genetic mechanisms underlying disrupted cortical circuit formation, and the dramatically elevated risk for psychosis in 22q11DS are unknown. Most of the protein-coding genes within the region are highly brain-expressed [62], with several involved in early neurodevelopment. Some of these (e.g., *RANBP1, CDC45L*) are selectively expressed in cortical progenitors in the ventricular/subventricular zones, whereas others (e.g., *DGCR8*, involved in microRNA biogenesis) are more broadly expressed in cortical neurons [63]. As *RANBP1* plays a role in rapidly dividing precursors in the developing brain, hemizygosity of this gene may lead to a reduction in the overall pool of cortical radial glial progenitors [64], and thus smaller cortical area. 22q11DS mouse models show widespread deficits in dendritic complexity and spine density, altered synaptic plasticity, and reduced hippocampal-prefrontal synchrony, changes that correlates with working memory impairments [65]. However, further studies are needed to isolate the precise genes responsible for the elevated psychosis risk and pattern of neuroanatomic abnormalities observed here.

One key advantage of this study is that we were able to conduct all analyses on raw data, ensuring consistent data processing and allowing vertex-wise analyses, results of which were highly consistent with ROI analyses. Some limitations, however, must be noted. The cross-site variability in age, stage of the disease, incidence of psychosis, and distribution of deletion types potentially confounded cortical measures. For this reason, we matched site/data set, sex, and age in several comparisons to address this variability. Given that only ~10% of 22q11.2 deletions overall are of the A–B type [2], this group is necessarily small; although effect sizes for SA differences were large, these findings nevertheless warrant replication in independent samples. Further, some 22q11DS subjects without psychosis in the current analyses might develop symptoms at a later point, so their inclusion in the non-psychotic group would likely have attenuated real group differences. Also, investigation of the neuroanatomic effects of other common associated comorbidities of 22q11DS (e.g., cardiac defects, autism spectrum disorders) was outside the scope of this study, but should be pursued in follow-up studies in similarly sized samples.

This genetically-defined neurodevelopmental condition offers a biologically tractable framework to dissect genetic mechanisms underlying brain phenotypes associated with complex neuropsychiatric disorders. Importantly, the brain phenotype of 22q11DS + Psychosis is substantially shared with idiopathic schizophrenia, suggesting that genetic subtypes of psychosis can provide insights into brain mechanisms associated with psychosis more broadly. Currently, a large-scale whole genome sequencing study (International 22q11.2 Brain-Behavior Consortium; IBBC) [44] is underway to investigate both rare and common variants that may contribute to psychosis risk in these patients [66]. This large-scale ‘genetics first’ approach, in combination with translational studies in animal and in vitro models, is likely to yield novel insights into the elusive molecular biology of psychosis.

## Disclaimer

These funding sources had no further role in study design, in the collection, analysis, and interpretation of the data, in the writing of the report, nor in the decision to submit the paper for publication.

## Electronic supplementary material

Supplement 1

Supplement 2

Supplement 3

Supplement 4
