# Supplementary material for: Large-scale mapping of cortical alterations in 22q11.2 deletion syndrome: Convergence with idiopathic psychosis and effects of deletion size
Source: Mol Psychiatry. 2018 Jun 13;25(8):1822–34. doi: 10.1038/s41380-018-0078-5 (PMC6292748; doi:10.1038/s41380-018-0078-5)
Supplement: Supplementary file 1 — Supplement 1 [file 41380_2018_78_MOESM1_ESM.docx]

**Supplemental Methods:**

**Study Participant Ascertainment and Assessment Procedures**

Across sites, all cases received a molecularly confirmed diagnosis of 22q11.2 deletion. All 22q11DS subjects included in the psychotic disorder group had a DSM schizophrenia spectrum psychotic disorder diagnosis (schizophrenia, schizoaffective disorder, or psychosis not otherwise specified), as determined via structured diagnostic interview conducted by a trained mental health professional at each site, and supplemented by collateral information and medical records (see **Supplementary Table S1** for details regarding study instruments and study inclusion/exclusion criteria). A cross-site reliability procedure was also undertaken, in which two investigators with clinical expertise independently reviewed a subset of representative cases from each site^1^. References provide additional detail regarding clinical characteristics of each study sample.

**Image Processing Details:**

Two independent imaging analysis approaches - region-of-interest (ROI) based analyses and surface-based analyses - were conducted using FreeSurfer’s imaging processing procedures.

1) ROI-based analyses: the cerebral cortex of each brain scan was parcellated into 68 detailed regions (34 from each hemisphere), based on the Desikan–Killiany atlas^2^, and measures of cortical thickness (CT) and surface area (SA) for each cortical region were obtained. These cortical measures were used for data visualization, selection of statistical models, and group comparisons. Statistical estimates including Cohen’s *d* and *p*-values from group comparisons were calculated, and a classification analysis between 22q11DS and control subjects was also performed, based on the cortical ROI measures. The selected statistical models were applied in the vertex-based analyses.

2) Surface-based analysis: By applying FreeSurfer’s reconstruction pipeline, local CT and SA were calculated and assigned to vertices on each reconstructed hemispheric surface model. CT is defined as the shortest distance between the pial and the gray/white surfaces^3^, and local SA is estimated by averaging the compression and expansion of triangles surrounding a vertex on a standardized tessellation when it is registered to individual brain surfaces^4^. The cortical maps were then smoothed with a Gaussian kernel of 10mm, and statistical analyses with the above selected models were conducted on each vertex of the cortical surface. The results from the surface-based analyses were reported as the primary analyses in this paper, as they provided more detailed information regarding cortical changes both within and across pre-defined ROIs; the corresponding results from the ROI-based analyses, in forms of both tables and scatterplots, were presented in the supplementary materials.

**Image Exclusion Criteria:**

Each image segmentation was individually examined by the first author (DS) by overlaying the segmentation label of each structure on the T1-weighted brain scan. Further, we pooled ROI measures from all sites and generated scatter-plots for each ROI in order to identify major outliers. A ROI measures was considered a statistical outlier if it was >2.698 standard deviations away from the global mean. For each subject’s MRI scan that was marked as a statistical outlier, DS re-inspected the segmentation in order to verify the scan was properly segmented. If a subject was a statistical outlier, but was properly segmented it was kept in the analysis. Otherwise the subject’s scan was removed (N=11).

**Medication effects on cortical measures**

Medication use at the time of MRI scan acquisition was grouped into typical antipsychotics (N=14), atypical antipsychotics (N=63), antidepressants (N=83), and anticonvulsants (mood stabilizers; N=22) (**Supplementary Table S8).** Only one subject had an available record of lithium use, so lithium use was excluded. Other psychotropic agents were excluded from the analysis due to high heterogeneity. Using all the available medication data, the effects of the medications on regional cortical measures were modeled in general linear models, including the medication categories as independent variables, while controlling for other confounding factors of site, sex, and age. The results are presented in Supplementary **Table S17a, b.)**

In a secondary analysis, we investigated the effects of antipsychotic medication in the 22q11DS-Psychosis group only to further reduce any confounding effect from psychotic diagnoses. Specifically, we conducted an ANCOVA analysis, controlling for site, sex, and age, to examine if there were differences in cortical measures among subjects who were on typical antipsychotics (N=8), atypical antipsychotics (N=36), both (N=5), vs. no antipsychotic (N=13) (see Supplementary **Table S18a,b)**.

**22q11DS Case vs Control Analyses Utilizing Mixed Linear Models**

Treating dataset/site as a random variable, mixed linear models were also used for comparison. Results from the mixed-effects models, where dataset/site was treated as a random effect, are included in **Supplementary Table S8a** (CT) and **S8b** (SA). The overall pattern of findings remained the same when mixed-effects models were used.

1. Gur RE, Bassett AS, McDonald-McGinn DM, Bearden CE, Chow E, Emanuel BS *et al.* A neurogenetic model for the study of schizophrenia spectrum disorders: the International 22q11.2 Deletion Syndrome Brain Behavior Consortium. *Mol Psychiatry* 2017; **22**(12)**:** 1664-1672.

2. Desikan RS, Segonne F, Fischl B, Quinn BT, Dickerson BC, Blacker D *et al.* An automated labeling system for subdividing the human cerebral cortex on MRI scans into gyral based regions of interest. *Neuroimage* 2006; **31**(3)**:** 968-980.

3. Fischl B, Salat DH, Busa E, Albert M, Dieterich M, Haselgrove C *et al.* Whole brain segmentation: automated labeling of neuroanatomical structures in the human brain. *Neuron* 2002; **33**(3)**:** 341-355.

4. Joyner AH, J CR, Bloss CS, Bakken TE, Rimol LM, Melle I *et al.* A common MECP2 haplotype associates with reduced cortical surface area in humans in two independent populations. *Proc Natl Acad Sci U S A* 2009; **106**(36)**:** 15483-15488.
