# Supplementary material for: Large-scale mapping of cortical alterations in 22q11.2 deletion syndrome: Convergence with idiopathic psychosis and effects of deletion size
Source: Mol Psychiatry. 2018 Jun 13;25(8):1822–34. doi: 10.1038/s41380-018-0078-5 (PMC6292748; doi:10.1038/s41380-018-0078-5)
Supplement: Supplementary file 2 — Supplement 2 [file 41380_2018_78_MOESM2_ESM.docx]

**SUPPLEMENTARY MATERIALS**

**Supplementary Figures**

Ggplot2, a R package, was used for the plotting and curve fitting for all the below supplementary figures. All curve fitting was done with Locally Weighted Scatter-plot Smoother (LOESS) smoothing (Cleveland, Grosse & Shyu 1992).

**Figure S1a.** Age Effects on Cortical Thickness for 22q11DS vs Control Comparison. A non-linear age effect can be observed in the majority of the regions and both hemispheres, which is further confirmed by comparing models with and without a quadratic age term (Table S3a). No clear pattern of group-age interactions can be seen (confirmed in Table 5a).

**Figure S1b.** Age Effects on Cortical Surface Area for 22q11DS vs Control Comparison. No clear non-linear age effect is observed in the majority of the regions or either hemisphere, which is further confirmed by comparing models with and without a quadratic age term (Table S3b). No clear pattern of group-age interactions can be seen (confirmed in Table 5b).

**Figure S2.** Relationships between Total Intracranial Volume (ICV) and Global CT/SA. The relationships were fitted between ICV and CT averaged across both hemispheres (left panel), and between ICV and total cortical surface area (right panel), respectively. No linear effect is observed for CT, while a clear linear effect is seen for SA.

**Figure S3.** Differences in Global Brain Measures, by Study Site. Scatter- and violin-plots were made to show the distribution of four global brain measures (ICV, total brain volume, mean cortical thickness, and total cortical surface area) for each of the 13 datasets from 10 study sites. Despite cross-site variations, consistent patterns of group differences are clearly observed. Red dots represent group means, and red vertical lines represent within-group standard deviations.

**Figure S4a.** Scatterplots of Regional Differences in Cortical Thickness between 22q11DS and Control Subjects. The data-points were adjusted for study site, sex, and both linear and quadratic age terms. Red dots represent group means, and red vertical lines represent with-group standard deviations of the residuals from the above modeling.

**Figure S4b**. Scatterplots of Regional Differences in Surface Area between 22q11DS and Control Subjects. The data-points were adjusted for study site, sex, age and ICV. Red dots represent group means, and red vertical lines represent within-group standard deviations of the residuals from the above modeling.

**Figure S5a.** Scatterplots of Regional Cortical Thickness Differences between 22q11DS+Psychosis vs. 22q11DS-No Psychosis Subjects. The data-points were adjusted for study site, sex, and age. Red dots represent group means, and red vertical lines represent within-group standard deviations of the residuals from the above modeling.

**Figure S5b**. Scatterplots of Regional Surface Area Differences between 22q11DS+Psychosis vs. 22q11DS-No Psychosis Subjects. The data-points were adjusted for study site, sex, age, and ICV. Red dots represent group means, and red vertical lines represent within-group standard deviations of the residuals from the above modeling.

**Figure S6a.** Scatterplots of Regional Cortical Thickness Differences between A-B Deletion, A-D Deletion and Control Subjects. The data-points were adjusted for study site, sex, age and quadratic age. Red dots represent group means, and red vertical lines represent within-group standard deviations of the residuals from the above modeling. P-values shown are for the overall differences across the 3 groups.

**Figure S6b.** Scatterplots of Regional Surface Area Differences between A-B Deletion, A-D Deletion and Control Subjects. The data-points were adjusted for study site, sex, age, and ICV. Red dots represent group means, and red vertical lines represent within-group standard deviations of the residuals from the above modeling. P-values shown are for the overall differences across the 3 groups.

**Figure S7a.** IQ Adjusted and Unadjusted Group Differences (Cohen’s d) for CT: 22q11DS Cases vs. Controls. Effect size values in Cohen’s d were plotted for the group differences in CT between 22q11DS cases and controls, contrasting those from the statistical model that adjusted for IQ with that didn’t adjust for IQ, in order to examine the influence of IQ on CT differences. Solid circles show Cohen’s d values unadjusted for IQ, and hollow circles show values adjusted for IQ. The black color indicates statistical significance after FDR corrections, while the gray color indicates non-significant results. The figure demonstrates that although the absolute effect sizes are reduced when adjusted for IQ, the significance levels and overall pattern of group differences largely remain the same.

**Figure S7b.** IQ Adjusted and Unadjusted Group Differences (Cohen’s d) for SA: 22q11DS Cases vs. Controls. Effect size values in Cohen’s d were plotted for the group differences in SA between 22q11DS cases and controls, contrasting those from the statistical model that adjusted for IQ with that didn’t adjust for IQ, in order to examine the influence of IQ on SA differences. Solid circles show Cohen’s d values unadjusted for IQ, and hollow circles show values adjusted for IQ. The black color indicates statistical significance after FDR corrections, while the gray color indicates non-significant results. The figure demonstrates that although the absolute effect sizes are reduced when adjusted for IQ, the significance levels and overall pattern of group differences largely remain the same.

**Figure S8.** IQ Adjusted and Unadjusted Group Differences (Cohen’s d) for CT: 22q11DS cases with psychosis and without psychosis. Effect size values in Cohen’s d were plotted for the group differences in CT between 22q11DS cases with a diagnosis of psychosis and without psychosis, contrasting those from the statistical model that adjusted for IQ with that didn’t adjust for IQ, in order to examine the influence of IQ on CT differences. Solid circles show Cohen’s d values unadjusted for IQ, and hollow circles show values adjusted for IQ. The black color indicates statistical significance after FDR corrections, while the gray color indicates non-significant results. The figure demonstrates that although the absolute effect sizes are slightly altered when adjusted for IQ, the significance levels and overall pattern of group differences largely remain the same.

**Supplementary Tables**

**Table S1**: Clinical characteristics of 22q11DS and control participants, across sites. Inclusion/exclusion criteria and instruments for diagnosing psychotic disorder and psychotic symptom severity, by site. References indicate representative publications for each study sample.

**Table S2:** Scanner acquisition parameters, by site. References indicate publications reporting on site-specific imaging data.

**Table S3a.** Quadratic Age Effects on Cortical Thickness: 22q11DS Cases vs Controls. The following two models 1) CT ~ Group + Site + Sex + Age, and 2) CT ~ Group + Site + Sex + Age + Age^2^, were compared using F-tests. Results showed that for the majority of regions, a significantly increased proportion of variance can be accounted for by including the quadratic term of age in the models.

**Table S3b**. Quadratic Age Effects on Surface Area. The following two models 1) SA ~ Group + Site + Sex + Age, and 2) SA ~ Group + Site + Sex + Age + Age^2^, were compared using F-tests. Results showed that for the majority of regions, no further proportion of variance is accounted for by including the quadratic term of age in the models.

**Table S4a**. Group x Sex Interactions on Cortical Thickness. The following two models 1) CT ~ Group + Site + Sex + Age, and 2) CT ~ Group + Site + Sex + Age + Group * Sex, were compared using F-tests. Results showed that for the majority of regions, no further proportion of variance is accounted for by including the interaction term in the models.

**Table S4b.** Group x Sex Interactions on Surface Area. The following two models 1) SA ~ Group + Site + Sex + Age, and 2) SA ~ Group + Site + Sex + Age + Diagnosis * Sex, were compared using F-tests. Results showed that for the majority of regions, no further proportion of variance is accounted for by including the interaction term in the models.

**Table S5a.** Group x Age Interactions on Cortical Thickness. The following two models 1) CT ~ Group + Site + Sex + Age + Age^2^, and 2) CT ~ Group + Site + Sex + Age + Age^2^ + Group * Age, were compared using F-tests. Results showed that for the majority of regions, no further proportion of variance is accounted for by including the interaction term in the models.

**Table S5b.** Group x Age Interactions on Surface Area. The following two models 1) SA ~ Group + Site + Sex + Age + ICV, and 2) CT ~ Group + Site + Sex + Age + ICV + Group * Age, were compared using F-tests. Results showed that for the majority of regions, no further proportion of variance is accounted for by including the interaction term in the models.

**Table S6a.** Linear vs Quadratic Age Effects on Cortical Thickness: 22q11DS- Psychosis vs. No Psychosis. The following two models 1) CT ~ Group + Site + Sex + Age, and 2) CT ~ Group + Site + Sex + Age + Age^2^, were compared using F-tests. Results showed that for all regions, no further proportion of variance is accounted for by including the quadratic term of age in the models.

**Table S6b**. Linear vs. Quadratic Age Effects on Surface Area: 22q11DS-Psychosis vs. No Psychosis. The following two models 1) SA ~ Group + Site + Sex + ICV + Age, and 2) SA ~ Group + Site + Sex + ICV + Age + Age^2^, were compared using F-tests. Results showed that for all regions, no further proportion of variance is accounted for by including the quadratic term of age in the models.

**Table S7a**. Linear vs. Quadratic Age Effects on Cortical Thickness: A-D and A-B Deletion and Controls. The following two models 1) CT ~ Group + Site + Sex + Age, and 2) CT ~ Group + Site + Sex + Age + Age^2^, were compared using F-tests. Results showed that for the majority of regions, a significantly increased proportion of variance can be accounted for by including the quadratic term of age in the models.

**Table S7b**. Linear vs. Quadratic Age Effects on Surface Area: A-D and A-B Deletion and Controls. The following two models 1) SA ~ Group + Site + Sex + ICV + Age, and 2) SA ~ Group + Site + Sex + ICV + Age + Age^2^, were compared using F-tests. Results showed that for all regions, no further proportion of variance is accounted for by including the quadratic term of age in the models.

**Table S8.** Use of Psychotropic Medications in 22q11DS subjects.

**Table S9.** Effect Sizes (Cohen's *D*) For Group Differences in Global Brain Metrics Across Sites. Differences in 4 global brain measures (ICV, total brain volume, mean cortical thickness, and total cortical surface area) between 22q11DS cases and controls were compared for each dataset/site separately. Consistent patterns of group differences can be observed across sites.

**Table S10a:** Group Differences in Cortical Thickness, 22q11DS Cases vs Controls: Effect size (Cohen’s *d*), 95% Confidence Interval (CI), Percent Difference, and P-Value. The following linear model was used for the group comparison: CT ~ Group + Site + Sex + Age + Age^2^. Degrees of freedom (df) =686 for all comparisons.

**Table S10b:** Group Differences in Surface Area, 22q11DS Cases vs Controls: Effect size (Cohen’s d), 95% Confidence Interval (CI), Percent Difference, and P-Value. The following linear model was used for the group comparison: SA ~ Group + Site + Sex + Age + ICV. Df= 686 for all comparisons.

**Table S11a:** Group Differences in Cortical Thickness, 22q11DS Cases vs Controls, Mixed Effect Models: Effect size (Cohen’s d), 95% Confidence Interval (CI), Percent Difference, and P-Value. The following mixed effect linear model was used for the group comparison: CT ~ Group + Sex + Age + Age^2^, random = ~ 1 | Site. Df=686 for all comparisons.

**Table S11b:** Group Differences in Surface Area, 22q11DS Cases vs Controls, Mixed Effect Models: Effect size (Cohen’s d), 95% Confidence Interval (CI), Percent Difference, and P-Value. The following mixed effect linear model was used for the group comparison: SA ~ Group + Sex + Age + ICV, random = ~ 1 | Site. Df = 686 for all comparisons.

**Table S12a:** Classification Analysis: Accuracy, Sensitivity and Specificity. Random divisions were applied to the whole dataset to obtain 20 training sets and 20 corresponding testing sets at a ratio of 3:1 in numbers of subjects. For each division, the glmnet algorithm was used to calculate the sensitivity, specificity, and accuracy, as well as the significance level, of group prediction. These above values were averaged over the 20 divisions.

**Table S12b:** Classification Analysis, GLMNet Weights for Cortical Structures. The glmnet algorithm was applied to the full dataset to obtain the weights of cortical measures, which indicate the contribution of each measure in predicting the group label of a subject. Note that since glmnet performs an intrinsic feature selection with L1-norm regularization, some cortical measures had a weight of zero and therefore did not contribute in the prediction models.

**Table S13a.** Demographics of Matched 22q11DS+Psychosis (N=60) and 22q11DS-No Psychosis Subjects (N=60).

**Table S13b.** Psychotropic Medications, IQ and Deletion Type in 22q11DS Subjects with and without Psychosis.

**Table S14a**. Differences in Cortical Thickness, 22q11DS Psychosis vs. 22q11DS-No Psychosis: Effect size (Cohen’s d), 95% Confidence Interval (CI), Percent Difference, and P-Value. The following linear model was used for the group comparison: CT ~ Group + Site + Sex + Age. Df=107 for all comparisons.

**Table S14b.** Differences in Surface Area, 22q11DS Psychosis vs. 22q11DS-No Psychosis: Effect size (Cohen’s d), 95% Confidence Interval (CI), Percent Difference, and P-Value. The following linear model was used for the group comparison: SA ~ Group + Site + Sex + Age + ICV. Df= 106 for all comparisons.

**Table S15a**. Demographics of Matched A-B and A-D Deletion and Control Subjects.

**Table S15b.** Psychotropic Medications, IQ and Psychosis in 22q11DS Subjects with A-B vs. A-D Deletions.

**Table S16a.** Comparison of Cortical Thickness Differences among Subjects with A-D deletions, A-B Deletions and Controls. The following linear model was used for the group comparison: CT ~ Group + Site + Sex + Age + Age^2^. Df=203 for all comparisons.

**Table S16b.** Comparison of Surface Area Differences between Subject with A-D deletions, A-B Deletions and Controls. The following linear model was used for the group comparison: SA ~ Group + Site + Sex + Age + ICV. Df=203 for all comparisons.

**Table S17a**. Effects of Psychotropic Medications on Cortical Thickness (t-values and p-values for comparison of 22q11DS patients on vs. not on each category of psychotropic medication).

**Table S17b**. Effects of Psychotropic Medications on Cortical Surface Area (t-values and p-values for comparison of 22q11DS subjects on vs. not on each category of psychotropic medication).

**Table S18a**. Effects of Antipsychotic Medications on Cortical Thickness in 22q11DS-Subjects with Psychotic Disorder. Group differences among subjects who were on typical antipsychotics, atypical antipsychotics, both types of antipsychotics, and no antipsychotics were compared using Analysis of Covariance (ANCOVA). P-values are from the overall differences among the 4 groups. Only the right inferior temporal cortex showed significant difference among all 68 regions from the analysis.

**Table S18b**. Effects of Antipsychotic Medications on Surface Area in 22q11DS Subjects with Psychotic Disorder. Group differences among subjects who were on typical antipsychotics, atypical antipsychotics, both types of antipsychotics, and no antipsychotics were compared using Analysis of Covariance (ANCOVA). P-values are from the overall differences among the 4 groups. No significant difference was found in any cortical regions from the analysis.

**Table S19a.** Effects of Covarying for Handedness on CT (Effect Sizes and P-Values). Effect size values in Cohen’s d were shown here for the group differences in CT between 22q11DS cases and controls, contrasting those from the statistical model that adjusted for handedness with that did not adjust for it, in order to examine the influence of handedness on group differences in CT. As indicated, Cohen’s d values and the significance levels remain almost the same whether handedness was adjusted for or not.

**Table S19b**. Effects of Covarying for Handedness on SA (Effect Sizes and P-Values). Effect size values in Cohen’s d were shown here for the group differences in SA between 22q11DS cases and controls, contrasting those from the statistical model that adjusted for handedness with that did not adjust for it, in order to examine the influence of handedness on group differences in SA. As indicated, Cohen’s d values and the significance levels remain almost the same whether handedness was adjusted for or not.

**Supplementary Table S1**: Clinical characteristics of 22q11DS and control participants, across sites: inclusion/exclusion criteria and instruments for diagnosing psychotic disorder and psychotic symptom severity, by site. References indicate representative publications for each study sample.

| **Site** | | **Study Inclusion and Exclusion criteria** | | **Instrument for psychiatric diagnosis**  **/ Rating psychotic symptoms severity** | | | **Instrument for IQ Assessment** | | **Citations** |
| --- | --- | --- | --- | --- | --- | --- | --- | --- | --- |
| UCLA (1,2) | | Inclusion Criteria 22q11DS and Controls:  1) no significant abuse of drugs or alcohol during the last 6 months or prior abuse/dependence likely to lead to central  nervous system impairment;  2) between 5 and 50 years of age;  3) sufficient acculturation and fluency in the English language to avoid invalidating research measures  Inclusion Criteria 22q11DS Only:  1) Confirmed diagnosis of 22q11.2 microdeletion (by FISH  or microarray)  2) no evidence of a comorbid neurological disorder (e.g., uncontrolled epilepsy, encephalitis);  3) Verbal IQ >= 60 in order to complete the Structured  Interview for Prodromal Syndromes  Inclusion Criteria Controls Only:  1) no evidence of current or past significant psychopathology;  2) self- and parent report of no prior treatment for psychiatric disorder;  3) no evidence of traumatic brain injury, or other  neurological disorder or impairment;  4) no history of significant medical complications likely to affect cognitive functioning (e.g., Type I diabetes, cancer, neural tube defects, etc.)  5) no first-degree relative has been diagnosed with or treated  for psychotic disorder; and  8) sex, age, race, and parental educational level comparable to that of the patient participants  6) verbal IQ >= 70 | | SCID1 interview (over 10) C-DISC2 (18 & under)  SIPS3  BPRS4 | | | WASI 57 | | Ho et al., 201214  Jalbrzikowski et al., 201215, 201316,  201417  Jonas et al., 201518  Schreiner et al., 201319, 201720 |
| State University of New  York at Upstate  (SUNY) | Inclusion Criteria 22q11DS and Controls:  1) Between the ages of 9 and 15 years of age at the 1st timepoint, or 12 and 18 years if they entered the study at the  2nd timepoint.  2) No orthodontia or paramagnetic implants.  3) Birthweight Over 2500 grams  4) No traumatic brain injury or loss of consciousness for >  15 minutes  5) No fetal exposure to drugs or alcohol  Inclusion Criteria 22q11DS Only:  Confirmed diagnosis of 22q11.2 microdeletion (by FISH or microarray)  Exclusion Criteria Controls Only:  1) History of severe psychiatric disorder in self or 1st degree relatives  2) Placement in a gifted or special education classroom  3) Seizure or other neurological / genetic disorder | | | SCID1  SIPS3  BPRS4  K-SADS-PL5 | | WISC-III 58 | | Radoeva et al., 201421,  Antshel et al., 201322, Radoeva et al., 201223, Kunwar et al.,201224,  Kates et al., 201125, Kates et al., 201126, Coman et al., 201027, Roizen et al., 201028, Antshel et al., 201029, Antshel et al., 200830, Kates et al., 200731, Antshel et al., 200732, Kates et al., 200733, Antshel et al., 200734, Aneja et al., 200735, Antshel et al., 200636, Kates et al., 200637 | |
| UC Davis | Inclusion Criteria 22q11DS Only:  Confirmed diagnosis of 22q11.2 microdeletion (by FISH or microarray)  Exclusion Criteria 22q11DS cases and Controls:  1) Brain infarct, CNS infection, head injury, other focal neurologic abnormality  2) Current or past use of antipsychotic medications  3) Existing diagnosis of psychosis | | | SCID1 | | WISC-IV 59  WASI 57 | | Scott et al., 201638,  Deng et al., 201539,  Stephenson et al., 201440 | |
| University of  Pennsylvania/Children’s Hospital of Philadelphia | Inclusion Criteria 22q11DS and Controls:  1) Age ≥8  2) Ability to provide informed consent/assent  3) English proficiency  4) Ambulatory and stable medical status  5) Estimated IQ >70  Inclusion Criteria 22q11DS Only:  Confirmed diagnosis of 22q11.2 microdeletion (by FISH or microarray)  Exclusion Criteria 22q11DS and Controls:  1) Pervasive developmental disorder per medical records or  mental retardation (IQ < 70)  2) Medical or neurological disorders that may affect brain function (e.g., uncontrolled seizures, head trauma, CNS tumor, and infection) or visual performance (e.g., blindness). | | | K-SADS-PL5  SIPS3 | | WISC 59  WAIS 60 (22q11Ds);  WRAT/Penn-CNB (controls) 61 | | Yi et al., 2013 41,  Niarchou et al., 201742,  Tang et al., 201743,  Tang et al., 201744 | |
| Utrecht University  Medical Center, Netherlands | Inclusion Criteria 22q11DS:  1) Confirmed diagnosis of 22q11.2 deletion  2) age >=12  3) VIQ >55 | | | K-SADS-PL5 | | Dutch version of: WISC-III 58  or  WISC-R 62;  WAIS –III 60 | | Fiksinski et al., 201745 | |
| Kings College (Institute  of Psychiatry), London | Inclusion Criteria 22q11DS and Controls:  1) no significant abuse of drugs or alcohol during the last 6 months or prior abuse/dependence likely to lead to central nervous system impairment;  2) between 5 and 50 years of age;  3) sufficient acculturation and fluency in the English language to avoid invalidating research measures  Inclusion Criteria 22q11DS Only:  1) Confirmed diagnosis of 22q11.2 microdeletion by FISH  test  2) no evidence of a comorbid neurological disorder (e.g., uncontrolled epilepsy, encephalitis);  3) Verbal IQ >= 60 in order to complete the Structured  Interview for Prodromal Syndromes  Inclusion Criteria Controls Only:  1) no evidence of current or past significant psychopathology or psychiatric disorders;  2) no evidence of traumatic brain injury, or other neurological disorder or impairment;  3) no history of significant medical complications likely to affect cognitive functioning (e.g., Type I diabetes, cancer,  neural tube defects, etc.)  4) no first-degree relative has been diagnosed with or treated for psychotic disorder; and  5) sex, age, race, and parental educational level comparable to that of the patient participants  6) verbal IQ >= 70 | | | SCID1  C-DISC2  SIPS3  BPRS4 | | WASI 57 | | In preparation | |
| Maastricht | Inclusion Criteria 22q11DS and Controls:  1) age >18 years  Inclusion Criteria 22q11DS Only:  Confirmed diagnosis of 22q11.2 microdeletion (by FISH or microarray)  Exclusion Criteria 22q11DS and Controls:  Present substance use or history of abuse or dependency, neurological affliction, or pregnancy. | | PANSS6  MINI7  PAS-ADD8 | | Dutch version of WAIS –III 60  Dutch version of the National Adult Reading Test (DART)63 | | | Bakker et al., 201646  Da Silva Alves et al., 201147 | |
| Cardiff | Inclusion Criteria 22q11DS and Controls:  1) Age ≥ 10  Inclusion Criteria 22q11DS Only:  Confirmed diagnosis of 22q11.2 microdeletion (by FISH or microarray)  Exclusion Criteria 22q11.2DS and Controls: Contraindications for MRI scanning (e.g. MRI incompatible implants or prostheses) | | CAPA 9  SIPS3  Adults only: SIPS3  PANSS6  PAS-ADD8  SCID-II 10  SAPS11  SANS12  SPI-A13 | | WASI 57 | | | Monks et al., 201448,  Niarchou et al., 201449, 201550,  Chawner et al., In press51 | |
| Newcastle | Inclusion Criteria 22q11DS and Control:  1) English language fluency  Inclusion Criteria 22q11DS Only:  Confirmed diagnosis of 22q11.2 microdeletion (by FISH or microarray)  Exclusion Criteria 22q11DS and Controls:  Clinically detectable medical disorder known to affect brain structure (e.g., hypertension), or a history of head injury.  Exclusion Criteria Controls Only:  1) The presence of a genetic disorder, mental health  problems, a history of severe head injury, seizure disorder, or other ocular, neurological or major medical problems that could influence task performance. | | K-SADS-PL5  SCID1 | | WISC-III 58  WASI 57 | | | Campbell et al., 201052, 201553,  McCabe et al., 201254, 201355,201456 | |
| Toronto 1 | Inclusion Criteria 22q11.2DS only:  1) Between the ages of 30 and 65 years 2) Molecularly diagnosed with 22q11.2DS 3) No other diagnosed genomic disorder other than 22q11.2DS  Inclusion Criteria Controls only: 1) Between the ages of 30 and 65 years 2) No diagnosed genomic disorder 3) No diagnosed psychiatric disorders 4) No diagnosed motor/movement disorders 5) No history or current use of street drugs 6) No diagnosed major feature of 22q11.2DS: Psychosis, Mood disorder, Anxiety disorder, Intellectual disability, Congenital heart disease, Parkinson’s disease  Exclusion Criteria 22q11.2DS and Controls: 1) Metal implants contraindicated to MRI 2) Cardiac pacemaker 3) Pregnancy 4) Prior history of stroke 5) Deep brain stimulator | | SCID^1^ | | WAIS –III 60 | | | Butcher et al., 2017^57^,  Chow et al., 2006^58^,  Butcher et al., 2012^59^,  Van et al., 2016^60^ | |
| Toronto 2 | Inclusion criteria (22q11DS only): 1) 22q11.2 deletion confirmed by FISH or microarray studies  2) Age > 16 3) No known prior traumatic brain injury  Exclusion criteria 22q11.2DS and Controls: 1) Contraindications to MRI scanning (metal implants, metal artificial heart valves, cardiac pacemaker, pregnancy, recent surgery (<6 months), intra ocular metal) 2) Excessive anxiety or agitation | | SCID^1^ | | WAIS –III 60 | | | Chow et al., 2011^61^ | |

1 Structured Clinical Interview for DSM-IV-TR Axis I Disorders, Research Version, Patient Edition. (SCID-I/P).

First MB, Spitzer RL, Gibbon M, Williams JBW. New York: Biometrics Research, New York State Psychiatric Institute; 2002.

2 Diagnostic Interview Schedule for Children.

Shaffer D, Schwab-Stone M, Fisher P, Cohen P, Piacentini J, Davies M et al. The Diagnostic Interview Schedule for Children–Revised Version (DISC-R), I: preparation, field testing, interrater reliability, and acceptability. J *Am Acad Child Adolesc Psychiatry* 1993; **32**:643–650.

3 Structured Interview for Prodromal Syndromes.

McGlashan TH*.* Structured Interview for Prodromal Syndromes (SIPS). Yale University: New Haven, 2001.

4 Brief Psychiatric Rating Scale.

Ventura J, Green MF, Shaner A, Liberman RP. Training and quality assurance with the Brief Psychiatric Rating Scale: The drift busters. *Int J Methods*

*Psychiatr Res* 1993;**3**: 221–244.

5 Kiddie Schedule for Affective Disorders and Schizophrenia- Present and Lifetime Version.

Kaufman J, Birmaher B, Brent D, Rao U, Flynn C, Moreci P et al. Schedule for affective disorders and schizophrenia for school-age children-present and lifetime version (K-SADS-PL): initial reliability and validity data. *J Am Acad Child Adolesc Psychiatry* 1997; **36**:980–988.

6 Positive and Negative Syndrome Scale.

Kay SR*,* Fiszbein A*,* Opler LA*.* The positive and negative syndrome scale (PANSS) for schizophrenia*. Schizophr Bull* 1987*;***13***:*261*–*276*.*

7 Mini International Neuropsychiatric Interview.

Sheehan DV, Lecrubier Y, Harnett-Sheehan K, Janavs J, Weiller E, Keskiner A et al. The validity of the Mini International Neuropsychiatric Interview (MINI) according to the SCID-P and its reliability. *Eur Psychiatry* 1997; **12**:232–241.

8 Psychiatric Assessment Schedules for Adults with Developmental Disabilities.

Moss S*,* Patel P*,* Prosser H*,* Goldberg D*,* Simpson N*,* Rowe S et al*.* Psychiatric morbidity in older people with moderate and severe learning disability. I: Development and reliability of the patient interview (PAS-ADD)*. Br J Psychiatry* 1993; **163***:*471*–*480*.*

9 Child and Adolescent Psychiatric Assessment (CAPA) version 4.2.

Angold A, Prendergast M, Cox A, Harrington R, Simonoff E, Rutter M. The Child and Adolescent Psychiatric Assessment (CAPA). *Psychol Med* 1995; **25**:739–753.

10 Structured Clinical Interview for DSM_IV Axis II Personality Disorders.

First MB, Spitzer RL, Gibbon M, Williams JBW. Structured Clinical Interview for DSM-IV Axis I Disorders: Patient Edition Biometrics Research. Biometrics Research, New York State Psychiatric Institute: New York, 2002.

11 Scale for the Assessment of Positive Symptoms.

Andreasen NC. Scale for the assessment of positive symptoms. University of Iowa; Iowa City, 1984.

12 Scale for the Assessment of Negative Symptoms.

Andreasen NC. Scale for the assessment of negative symptoms. University of Iowa; Iowa City*,* 1984*.*

13 Schizophrenia Proneness Instrument-Adult.

Schultze-Lutter F, Addington J, Ruhrmann S, Klosterkötter J. Schizophrenia Proneness Instrument (SPI-A). Giovanni Fioriti: Rome, Italy, 2007.

14 Ho JS, Radoeva PD, Jalbrzikowski M, Chow C, Hopkins J, Tran W et al. Deficits in mental state attributions in individuals with 22q11.2 deletion syndrome (velo-cardio-facial syndrome). *Autism* 2012; **5**: 407-418.

15 Jalbrzikowski M, Carter C, Senturk D, Chow C, Hopkins JM, Green MF et al. Social cognition in 22q11.2 microdeletion syndrome: Relevance to psychosis. *Schizophr Res* 2012; **142**(0)**:** 99-107.

16 Jalbrzikowski M, Jonas R, Senturk D, Patel A, Chow C, Green MF et al. Structural abnormalities in cortical volume, thickness, and surface area in 22q11.2 microdeletion syndrome: Relationship with psychotic symptoms. *NeuroImage: Clin* 2013; **3:** 405–415.

17 Jalbrzikowski M, Villalon-Reina JE, Karlsgodt KH, Senturk D, Chow C, Thompson PM et al. Altered white matter microstructure is associated with social cognition and psychotic symptoms in 22q11.2 microdeletion syndrome. *Front Behav Neurosci* 2014; **8:** 393.

18 Jonas RK, Jalbrzikowski M, Montojo CA, Patel A, Kushan L, Chow CC et al. Altered brain structure-function relationships underlie executive dysfunction in 22q11.2 deletion syndrome. *Mol Neuropsychiatry* 2015; **1**: 235–246.

19 Schreiner MJ, Lazaro MT, Jalbrzikowski M, Bearden CE. Converging levels of analysis on a genomic hotspot for psychosis: Insights from 22q11.2 Deletion Syndrome. *Neuropharmacology* 2013; **68:** 157-173.

20 Schreiner M, Forsyth JK, Karlsgodt KH, Anderson AE, Hirsh N, Kushan L et al. Intrinsic connectivity network-based classification and detection of psychotic symptoms in youth with 22q11.2 deletions. *Cereb Cortex* 2017; **27:** 3294-3306.

21 Radoeva PD, Coman IL, Salazar CA, Gentile KL, Higgins AM, Middleton FA et al. Association between autism spectrum disorder in individuals with velocardiofacial (22q11.2 deletion) syndrome and PRODH and COMT genotypes. *Psychiatr Genet* 2014; **24:**269-272.

22 Antshel KM, Hendricks K, Shprintzen R, Fremont W, Higgins AM, Faraone SV et al. The longitudinal course of attention deficit/hyperactivity disorder in velo-cardio-facial syndrome. *J Pediatr* 2013;**163**:187-193.

23 Radoeva PD, Coman IL, Antshel KM, Fremont W, McCarthy CS, Kotkar A et al. Atlas-based white matter analysis in individuals with velo-cardio-facial syndrome (22q11.2 deletion syndrome) and unaffected siblings. *Behav Brain Funct* 2012;**8**:38.

24 Kunwar A, Ramanathan S, Nelson J, Antshel KM, Fremont W, Higgins AM, et al. Cortical gyrification in velo-cardio-facial (22q11.2 deletion) syndrome: a longitudinal study. *Schizophr Res* 2012;**137**:20-25.

25 Kates WR, Antshel KM, Faraone SV, Fremont WP, Higgins AM, Shprintzen RJ et al. Neuroanatomic predictors to prodromal psychosis in velocardiofacial syndrome (22q11.2 deletion syndrome): a longitudinal study. *Biol Psychiatry* 2011;**69**:945-952.

26 Kates WR, Bansal R, Fremont W, Antshel KM, Hao X, Higgins AM et al. Mapping cortical morphology in youth with velocardiofacial (22q11.2 deletion)

syndrome. *J Am Acad Child Adolesc Psychiatry* 2011;**50**:272-282.

27 Coman IL, Gnirke MH, Middleton FA, Antshel KM, Fremont W, Higgins AM et al. The effects of gender and catechol O-methyltransferase

(COMT) Val108/158Met polymorphism on emotion regulation in velo-cardio-facial syndrome (22q11.2 deletion syndrome): An fMRI study. *Neuroimage*

2010;**53**:1043-1050.

28 Roizen NJ, Higgins AM, Antshel KM, Fremont W, Shprintzen R, Kates WR. 22q11.2 deletion syndrome: are motor deficits more than expected for IQ level? *J Pediatr* 2010;**157**:658-661.

29 Antshel KM, Shprintzen R, Fremont W, Higgins AM, Faraone SV, Kates WR. Cognitive and psychiatric predictors to psychosis in velocardiofacial syndrome:ma 3-year follow-up study. *J Am Acad Child Adolesc Psychiatry* 2010;**49**:333-344.

30 Antshel KM, Peebles J, AbdulSabur N, Higgins AM, Roizen N, Shprintzen R et al. Associations between performance on the

Rey-Osterrieth Complex Figure and regional brain volumes in children with and without velocardiofacial syndrome. *Dev Neuropsychol* 2008;**33**:601-622.

31 Kates WR, Antshel KM, Fremont WP, Shprintzen RJ, Strunge LA, Burnette CP et al. Comparing phenotypes in patients with idiopathic autism to patients with velocardiofacial syndrome (22q11 DS) with and without autism*. Am J Med Genet A* 2007;**143A**:2642-2650.

32 Antshel KM, Aneja A, Strunge L, Peebles J, Fremont WP, Stallone K et al. Autistic spectrum disorders in velo-cardio facial syndrome (22q11.2 deletion). *J Autism Dev Disord* 2007;**37**:1776-1786.

33 Kates WR, Krauss BR, Abdulsabur N, Colgan D, Antshel KM, Higgins AM et al. The neural correlates of non-spatial working memory in velocardiofacial syndrome (22q11.2 deletion syndrome). *Neuropsychologia* 2007;**45**:2863-2873.

34 Antshel KM, Stallone K, Abdulsabur N, Shprintzen R, Roizen N, Higgins AM et al. Temperament in velocardiofacial syndrome. *J Intellect Disabil Res*

2007;**51**:218-227.

35 Aneja A, Fremont WP, Antshel KM, Faraone SV, AbdulSabur N, Higgins AM et al. Manic symptoms and behavioral dysregulation in youth with velocardiofacial syndrome (22q11.2 deletion syndrome). *J Child Adolesc Psychopharmacol* 2007;**17**:105-114.

36 Antshel KM, Fremont W, Roizen NJ, Shprintzen R, Higgins AM, Dhamoon A et al. ADHD, major depressive disorder, and simple phobias are prevalent psychiatric conditions in youth with velocardiofacial syndrome*. J Am Acad Child Adolesc Psychiatry* 2006;**45**:596-603.

37 Kates WR, Antshel KM, Abdulsabur N, Colgan D, Funke B, Fremont W et al. A gender-moderated effect of a functional COMT polymorphism on prefrontal brain morphology and function in velo-cardio-facial syndrome (22q11.2 deletion syndrome). *Am J Med Genet B Neuropsychiatr Genet* 2006;**141B**:274-280.

38 Scott JA, Goodrich-Hunsaker N, Kalish K, Lee A, Hunsaker MR, Schumann CM et al. The hippocampi of children with chromosome 22q11.2 deletion syndrome have localized anterior alterations that predict severity of anxiety. *J Psychiatry Neurosci* 2016;**41**:203-213.

39 Deng Y, Goodrich-Hunsaker NJ, Cabaral M, Amaral DG, Buonocore MH, Harvey D et al. Disrupted fornix integrity in children with chromosome 22q11.2 deletion syndrome. *Psychiatry Res* 2015;**232**:106-114.

40 Stephenson DD, Beaton EA, Weems CF, Angkustsiri K, Simon TJ. Identifying patterns of anxiety and depression in children with chromosome 22q11.2 deletion syndrome: comorbidity predicts behavioral difficulties and impaired functional communications. *Behav Brain Res* 2015;**276**:190-198.

41 Yin JJ, Tang SX, McDonald-McGinn DM, Calkins ME, Whinna DA, Souders MC *et al*. Contribution of congenital heart disease to neuropsychiatric outcome in school-age children with 22q11.2 deletion syndrome. *Am J Med Genet B Neuropsychiatr Genet* 2014; **0**:137–147.

42 Niarchou M, Moore TM, Tang SX, Calkins ME, McDonald-McGuinn DM, Zackai EH et al.The dimensional structure of psychopathology in 22q11.2 Deletion Syndrome*. J Psychiatr Res* 2017;**92**:124-131.

43 Tang SX, Moore TM, Calkins ME, Yi JJ, McDonald-McGinn DM, Zackai EH et al. Emergent, remitted and persistent psychosis-spectrum symptoms in

22q11.2 deletion syndrome. *Transl Psychiatry* 2017;**7**:e1180.

44 Tang SX, Moore TM, Calkins ME, Yi JJ, Savitt A, Kohler CG et al. The Psychosis Spectrum in 22q11.2 Deletion Syndrome Is Comparable to That of

Nondeleted Youths. *Biol Psychiatry* 2017;**82**:17-25.

45 Fiksinski AM, Breetvelt EJ, Duijff SN, Bassett AS, Kahn AS, Vorstman JA. Autism spectrum and psychosis risk in the 22q11.2 deletion syndrome. Findings from a prospective longitudinal study. *Schizophr Res* 2017; e-pub ahead of print 21 January 2017; doi: 10.1016/j.schres.2017.01.032.

46 Bakker G, Caan MWA, Vingerhoets WAM, da Silva-Alves F, de Koning M, Boot E, *et al.* Cortical morphology differences in subjects at increased vulnerability for developing a psychotic disorder: A comparison between subjects with ultra-high risk and 22q11.2 deletion syndrome. *PLoS ONE* 2016; **11:** e0159928.

47 Da Silva-Alves F, Schmitz N, Bloemen O, can der Meer J, Meijer J, Boot E et al. White matter abnormalities in adults with 22q11 deletion syndrome with and without schizophrenia. *Schizophr Res* 2011;**132**: 75-83.

48 Monks S, Niarchou M, Davies AR, Walters JT, Williams N, Owen MJ *et al.* Further evidence for high rates of schizophrenia in 22q11.2 deletion syndrome. *Schizophr Res* 2014;**153**: 231-236.

49 Niarchou M, Zammit S, van Goozen SHM, Thapar A, Tierling HM, Owen MJ et al. Psychopathology and cognition in children with 22q11.2 deletion syndrome. *Br J Psychiatry* 2014; **204**: 46–54.

50 Niarchou M, Martin J, Thapar A, Owen MJ, van den Bree MBM. The clinical presentation of attention deficit‐hyperactivity disorder (ADHD) in children with 22q11.2 deletion syndrome. *Am J Med Genet* 2015; **168**: 730–738.

51 Chawner S, Doherty JL, Moss H, Niarchou M, Walters J, Owen MJ et al. Case-control study finds no evidence that 22q11.2 Deletion Syndrome is associated with cognitive deterioration. *Br J Psychiatry* (in press)

52 Campbell LE, Azuma R, Ambery F, Stevens A, Smith A, Morris RG et al. Executive functions and memory abilities in children with 22q11.2 deletion syndrome. *Aust N Z J Psychiatry* 2010; **44:** 364-371.

53 Campbell LE, McCabe KL, Melville JL, Strutt PA, Schall U. Social cognition dysfunction in adolescents with 22q11.2 deletion syndrome (velo-cardio-facial syndrome): relationship with executive functioning and social competence/functioning*. J Intellect Disabil Res* 2015; **59:** 845-859.

54 McCabe KL & Carr VJ. Relationship between childhood adversity and clinical and cognitive features in schizophrenia. *J Psychiatr Res* 2012; **46**: 600-607.

55 McCabe KL, Melville JL, Rich D, Strutt PA, Cooper G, Loughland CM et al*.* Divergent patterns of social cognition performance in autism and 22q11.2 deletion syndrome (22q11DS). *J Autism Dev Disord* 2013; **43**: 1926-1934.

56 McCabe KL, Atkinson RJ, Cooper G, Melville JL, Harris J, Schall U et al. Pre-pulse inhibition and antisaccade performance indicate impaired attention modulation of cognitive inhibition in 22q11.2 deletion syndrome. *J Neurodev Disord* 2014; **6**:38.

57Wechsler Abbreviated Scale of Intelligence

Wechsler D. Wechsler abbreviated scale of intelligence. Harcourt Brace & Company. New York, NY: The Psychological Corporation; 1999.

58Wechsler Intelligence Scale for Children – Third Edition

Wechsler D.  Wechsler Intelligence Scale for Children.3rd ed. San Antonio, TX: Psychological Corp; 1991.

59Wechsler Intelligence Scale for Children –Fourth Edition

Wechsler D.  Wechsler Intelligence Scale for Children.4th ed. San Antonio, TX: Psychological Corp; 2003.

60Wechsler Adult Intelligence Scale – Third Edition

Wechsler D. Wechsler adult intelligence scale - 3rd ed. San Antonio,TX: Psychological Corp; 1997.

61Wide range achievement test – Fourth edition (WRAT)

Wilkinson GS & Robertson GJ. Wide range achievement test – Fourth edition: Professional manual. Lutz, FL: Psychological Assessment Resources; 2006.

62Wechsler Intelligence Scale for Children—Revised

Wechsler D. Manual of the Wechsler intelligence scale for children-revised. New York: Psychological Corp; 1974.

63Dutch version of the National Adult Reading Test (DART)

Schmand B, Bakker D, Saan R, Louman J. De Nederlandse Leestest voor Volwassenen (NLV): een maat voor het premorbide intelligentieniveau [The Dutch Adult Reading Test (DART): A measure of premorbid intelligence]. *Tijdschrift voor Gerontologie en Geriatrie* 1991; **22**:15-19.

**Supplementary Table 2:** Scanner acquisition parameters, by site. References indicate publications reporting on site-specific imaging data.

| **Site** | **Scanner vendor and type** | **Sequence** | **Field Strength** | **Acquisition Direction** | **# of Slices** | **Slice Thickness (mm)** | **Voxel Size (mm3)** | **TI (ms)** | **TE (ms)** | **TR (ms)** | **Flip Angle** | **Citation** |
| --- | --- | --- | --- | --- | --- | --- | --- | --- | --- | --- | --- | --- |
| UCLA 1 | Siemens Tim Trio | MPRAGE | 3T | sagittal | 160 | 1.2 | 1.0x1.0x1.2 | 900 | 2.86 | 2300 | 9 | Lin et al., 2017^1^; Jalbrzikowski et al., 2017 ^2^;  Jonas et al., 2015^3^; Montojo et al., 2015^4^; Montojo et al., 2014^5^; Jalbrzikowski et al., 2013^6^ |
| UCLA 2 | Siemens Tim Trio | MPRAGE | 3T | sagittal | 160 | 1.2 | 1.0x1.0x1.2 | 900 | 2.91 | 2300 | 9 | Lin et al., 2017^1^; Jalbrzikowski et al., 2017 ^2^;  Jonas et al., 2015^3^; Montojo et al., 2015^4^; Montojo et al., 2014^5^; Jalbrzikowski et al., 2013^6^ |
| SUNY | Siemens Tim Trio | MPRAGE_SAG_BWM | 3T | sagittal | 176 | 1 | 1.0x1.0x1.0 | 1100 | 3.31 | 2530 | 7 | Radoeva et al., 2012^7^,  Kunwar et al.,2012^8^,  Kates et al., 2011^9^,  Kates et al., 2011^10^,  Coman et al., 2010^11^,  Antshel et al., 2008^12^,  Kates et al., 2007^13^,  Kates et al., 2006^14^ |
| UC Davis 1 | Siemens Tim Trio | MPRAGE_SAGMagdeburgIPAT | 3T | axial | 160 | 1 | 1.0x1.0x1.0 | 1100 | 2.93 | 1820 | 12 | DeBoer et al., 2007^15^, Simon et al., 2008^16^ |
| UC Davis 2 | Siemens Tim Trio | MPRAGE_0.9mm iso w/flow comp | 3T | sagittal | 192 | 0.9 | 0.9x0.9x0.9 | 1100 | 4.37 | 2200 | 7 | Beaton et al., 2010^17^ Srivastavaet al., 2012^18^ Villalon Reina et al., 2013^19^,  Deng et al., 2015^20^, Scott et al., 2015^21^ |
| University of Penn | Siemens | MPRAGE | 3T | axial | 160 | 1 | 0.9375x  0.9375x1.0 | 1100 | 3.51 | 1810 | 9 | Schmitt et al., 2015^22^, 2016^23^ |
| Utrecht | Philips Achieva, since March 2016 Philips Ingenia | 3D T1TFE | 3T | axial | 160 | 1 | 0.875x0.875x1 | Min. TI delay: 821.929016 | 4.6 | 9960 | 8 |  |
| IoP | GE | MPRAGE | 3T | sagittal | 166 | 1.2 | 1.0x1.0x1.0 | 650 | 2.9 | 6900 | 8 | In preparation |
| Maastricht | Philips Intera | MPRAGE | 3T | axial | 120 | 1.2 | 1.17×1.17×1.20 | TI delay: 807.8926 | 4.6 | 9800 | 8 | Bakker et al., 2016^24^  Da Silva Alves et al., 2011^25^ |
| Cardiff | GE | 3D FSPGR | 3T | oblique-axial | 172 | 1 | 1.0x1.0x1.0 | 450 | 3 | 7900 | 20 |  |
| Cardiff | Siemens Prisma | MPRAGE | 3T | sagittal | 176 | 1 | 1.0x1.0x1.0 | 850 | 3.06 | 2300 | 9 |  |
| Newcastle | Siemens Avanto | MPRAGE | 1.5T | sagittal | 176 | 1 | 0.98x0.98x1.0 | 1100 | 4.3 | 1980 | 15 |  |
| Toronto 1 | GE | 3D T1 FSPGR | 3T | sagittal | 200 | 0.99 | 0.9 | 650 | 3 | 6700 | 8 | Butcher et al., 2017^26^ |
| Toronto 2 | GE Signa | 3D T1 Inversion-prepped SPGR | 1.5T | coronal | 124 | 1.5 | 1.5x0.78x0.78 | 300 | 5.1 | 1200 | 20 | Chow et al., 2011^27^ |

^1^ Lin A, Ching CRK, Vajdi A, Sun D, Jonas RK, Jalbrzikowski M et al. Mapping 22q11.2 gene dosage effects on brain morphometry. *J Neurosci* 2017; **37**: 6183-6199.

^2^ Jalbrzikowski M, Ahmed KH, Patel A, Jonas R, Kushan L, Chow C et al. Categorical versus dimensional approaches to autism-associated intermediate phenotypes in 22q11.2 microdeletion syndrome. *Biol Psychiatry Cogn Neurosci Neuroimaging* 2017; **2**: 53-65.

^3^ Jonas RK, Jalbrzikowski M, Montojo CA, Patel A, Kushan L, Chow CC et al. Altered brain structure-function relationships underlie executive dysfunction in 22q11.2 deletion syndrome. *Mol Neuropsychiatry* 2015; **1**: 235–246.

^4^ Montojo CA, Congdon E, Hwang L, Jalbrzikowski M, Kushan L, Vesagas TK et al. Neural mechanisms of response inhibition and impulsivity in 22q11.2 deletion carriers and idiopathic attention deficit hyperactivity disorder. *Neuroimage Clin* 2015; **9:** 310–321.

^5^ Montojo CA, Ibrahim A, Karlsgodt KH, Chow C, Hilton AE, Jonas RK et al. Disrupted working memory circuitry and psychotic symptoms in 22q11.2 deletion syndrome. *Neuroimage Clin* 2014; **4:** 392–402.

^6^ Jalbrzikowski M, Jonas R, Senturk D, Patel A, Chow C, Green MF et al. Structural abnormalities in cortical volume, thickness, and surface area in 22q11.2 microdeletion syndrome: Relationship with psychotic symptoms. *Neuroimage Clin* 2013; **3:** 405–415.

^7^ Radoeva PD, Coman IL, Antshel KM, Fremont W, McCarthy CS, Kotkar A et al. Atlas-based white matter analysis in individuals with velo-cardio-facial syndrome (22q11.2 deletion syndrome) and unaffected siblings. *Behav Brain Funct* 2012;**8**:38.

^8^ Kunwar A, Ramanathan S, Nelson J, Antshel KM, Fremont W, Higgins AM, et al. Cortical gyrification in velo-cardio-facial (22q11.2

deletion) syndrome: a longitudinal study. *Schizophr Res* 2012;**137**:20-25.

^9^ Kates WR, Antshel KM, Faraone SV, Fremont WP, Higgins AM, Shprintzen RJ et al. Neuroanatomic predictors to prodromal psychosis in velocardiofacial syndrome (22q11.2 deletion syndrome): a longitudinal study. *Biol Psychiatry* 2011;**69**:945-952.

^10^ Kates WR, Bansal R, Fremont W, Antshel KM, Hao X, Higgins AM et al. Mapping cortical morphology in youth with velocardiofacial (22q11.2 deletion) syndrome. *J Am Acad Child Adolesc Psychiatry* 2011;**50**:272-282.

^11^ Coman IL, Gnirke MH, Middleton FA, Antshel KM, Fremont W, Higgins AM et al. The effects of gender and catechol O-methyltransferase

(COMT) Val108/158Met polymorphism on emotion regulation in velo-cardio-facial syndrome (22q11.2 deletion syndrome): An fMRI study. *Neuroimage* 2010;**53**:1043-1050.

^12^ Antshel KM, Peebles J, AbdulSabur N, Higgins AM, Roizen N, Shprintzen R et al. Associations between performance on the

Rey-Osterrieth Complex Figure and regional brain volumes in children with and without velocardiofacial syndrome. *Dev Neuropsychol* 2008;**33**:601-622.

^13^ Kates WR, Krauss BR, Abdulsabur N, Colgan D, Antshel KM, Higgins AM et al. The neural correlates of non-spatial working memory in

velocardiofacial syndrome (22q11.2 deletion syndrome). *Neuropsychologia* 2007;**45**:2863-2873.

^14^ Kates WR, Antshel KM, Abdulsabur N, Colgan D, Funke B, Fremont W et al. A gender-moderated effect of a functional COMT polymorphism on prefrontal brain morphology and function in velo-cardio-facial syndrome (22q11.2 deletion syndrome). *Am J Med Genet B Neuropsychiatr Genet* 2006;**141B**:274-280.

^15^ DeBoer T, Wu Z, Lee A, Simon TJ. Hippocampal volume reduction in children with chromosome 22q11.2 deletion syndrome is associated with cognitive impairment. *Behav Brain Funct* 2007; **3:** 54.

^16^ Simon TJ, Wu Z, Avants B, Zhang H, Gee JC, Stebbins GT. Atypical cortical connectivity and visuospatial cognitive impairments are related in children with chromosome 22q11.2 deletion syndrome. *Behav Brain Funct* 2008; **4:** 25.

^17^ Beaton EA, Qin Y, Nguyen V, Johnson J, Pinter JD, Simon TJ. Increased incidence and size of cavum septum pellicidum in children with chromosome 22q11.2 deletion syndrome. *Psychiatry Res* 2010; **181:** 108–113.

^18^ Srivastava S, Buonocore MH, Simon TJ. Atypical developmental trajectory of functionally significant cortical areas in children with chromosome 22q11.2 deletion syndrome. *Hum Brain Mapp* 2012; **33:** 213–223.

^19^ Villalon J, Jahanshad N, Beaton E, Toga AW, Thompson PM, Simon TJ. White matter microstructural abnormalities in girls with chromosome 22q11.2 deletion syndrome, Fragile X or Turner syndrome as evidenced by diffusion tensor imaging. *Neuroimage* 2013;**81:** 441-454.

^20^ Deng Y, Goodrich-Hunsaker NJ, Cabaral M, Amaral DG, Buonocore MH, Harvey D et al. Disrupted fornix integrity in children with chromosome 22q11.2 deletion syndrome. *Psychiatry Res* 2015;**232**:106-114.

^21^ Scott JA, Goodrich-Hunsaker N, Kalish K, Lee A, Hunsaker MR, Schumann CM et al. The hippocampi of children with chromosome 22q11.2 deletion syndrome have localized anterior alterations that predict severity of anxiety. *J Psychiatry Neurosci* 2016;**41**:203-213.

^22^ Schmitt, JE, Vandekar S, Yi J, Calkins ME, Ruparel K, Roalf DR, Gur RE. Aberrant Cortical Morphometry in the 22q11.2 Deletion Syndrome. *Biological Psychiatry* 2015; **78**(2)**:** 135–143.

^23^Schmitt JE, Yi J, Calkins ME, Ruparel K, Roalf DR, Cassidy A, et al. Disrupted anatomic networks in the 22q11.2 deletion syndrome. *Neuroimage Clin*. 2016;**12**:420-428.

^24^ Bakker G, Caan MWA, Vingerhoets WAM, da Silva-Alves F, de Koning M, Boot E, *et al.* Cortical morphology differences in subjects at increased vulnerability for developing a psychotic disorder: A comparison between subjects with ultra-high risk and 22q11.2 deletion syndrome. *PLoS ONE* 2016;**11:** e0159928.

^25^ Da Silva-Alves F, Schmitz N, Bloemen O, can der Meer J, Meijer J, Boot E et al. White matter abnormalities in adults with 22q11 deletion syndrome with and without schizophrenia. *Schizophr Res*  2011;**132**: 75-83.

^26^ Butcher NJ, Marras C, Pondal M, Rusjan P, Boot E, Christopher L et al. Neuroimaging and clinical features in adults with a 22q11.2 deletion at risk of Parkinson’s disease. *Brain* 2017; **140**: 1371-1383.

^27^ Chow EWC, Ho A, Wei C, Voormolen EHJ, Crawley AP,Bassett AS. Association of schizophrenia in 22q11.2 deletion syndrome and gray matter volumetric deficits in the superior temporal gyrus.*Am J Psychiatry* 2011; **168**: 522–52
