# Supplementary material for: Large-scale mapping of cortical alterations in 22q11.2 deletion syndrome: Convergence with idiopathic psychosis and effects of deletion size
Source: Mol Psychiatry. 2018 Jun 13;25(8):1822–34. doi: 10.1038/s41380-018-0078-5 (PMC6292748; doi:10.1038/s41380-018-0078-5)
Supplement: Supplementary file 3 — Supplement 3 [file 41380_2018_78_MOESM3_ESM.docx]

Supplementary Tables - Table of Contents

[Table S3a. Quadratic Age Effects on CT: 22q11DS Cases vs Controls 3](#_Toc507597097)

[Table S3b. Quadratic Age Effects on SA: 22q11DS Cases vs Controls 5](#_Toc507597098)

[Table S4a. Group x Sex Interactions on CT: 22q11DS Cases vs Controls 7](#_Toc507597099)

[Table S4b. Group x Sex Interactions on SA: 22q11DS Cases vs Controls 9](#_Toc507597100)

[Table S5a. Group x Age Interactions on CT: 22q11DS Cases vs Controls 11](#_Toc507597101)

[Table S5b. Group x Age Interactions on SA: 22q11DS Cases vs Controls 13](#_Toc507597102)

[Table S6a. Linear vs Quadratic Age Effects on Cortical Thickness: 22q11DS- Psychosis vs. No Psychosis 15](#_Toc507597103)

[Table S6b. Linear vs. Quadratic Age Effects on Surface Area: 22q11DS-Psychosis vs. No Psychosis 17](#_Toc507597104)

[Table S7a. Linear vs. Quadratic Age Effects on Cortical Thickness: A-D and A-B Deletion and Controls 19](#_Toc507597105)

[Table S7B. Linear vs. Quadratic Age Effects on Surface Area: A-D and A-B Deletion and Controls 21](#_Toc507597106)

[Table S8. Use of Psychotropic Medications in 22q11DS subjects. 23](#_Toc507597107)

[Table s9. Effect sizes (cohen's *d*) for global brain metrics across sites 24](#_Toc507597108)

[Table S10a: Group Differences in CT, 22q11DS Cases vs Controls. 25](#_Toc507597109)

[Table S10b: Group Differences in SA, 22q11DS Cases vs Controls. 27](#_Toc507597110)

[Table S11a: Group Differences in CT, 22q11DS Cases vs Controls, Mixed Effect Models. 29](#_Toc507597111)

[Table S11b: Group Differences in SA, 22q11DS Cases vs Controls, Mixed Effect Models. 31](#_Toc507597112)

[Table S12a. Classification Accuracy – 22q11DS Cases vs Controls 33](#_Toc507597113)

[Table S12b: Classification Analysis, GLMNet Weights for Cortical Structures 34](#_Toc507597114)

[Table S13a. Demographics of Matched 22q11DS+Psychosis (N=60) and 22q11DS-No Psychosis Subjects (N=60). 38](#_Toc507597115)

[Table S13b. Psychotropic Medications, IQ and Deletion Type in 22q11DS subjects with and without Psychosis. 39](#_Toc507597116)

[Table S14a. Differences in CT, 22q11DS Psychosis vs. 22q11DS-No Psychosis. 40](#_Toc507597117)

[Table S14b. Differences in SA, 22q11DS Psychosis vs. 22q11DS-No Psychosis. 42](#_Toc507597118)

[Table S15a. Demographics of Matched A-B and A-D Deletion and Control Subjects 44](#_Toc507597119)

[Table S15b. Psychotropic Medications, IQ and Psychosis in 22q11DS Subjects with A-B vs. A-D Deletions. 45](#_Toc507597120)

[Table S16a. Comparison of Cortical Thickness Differences among Subjects with A-D deletions, A-B Deletions and Controls. 46](#_Toc507597121)

[Table S16b. Comparison of Surface Area Differences between Subject with A-D deletions, A-B Deletions and Controls. 49](#_Toc507597122)

[Table S17a. Effects of Psychotropic Medications on CT. 51](#_Toc507597123)

[Table S17b. Effects of Psychotropic Medications on SA. 54](#_Toc507597124)

[Table S18a. Effects of Antipsychotic Medications on Cortical Thickness in 22q11DS-Subjects with Psychotic Disorder. 57](#_Toc507597125)

[Table S18b. Effects of Antipsychotic Medications on Surface Area in 22q11DS Subjects with Psychotic Disorder. 59](#_Toc507597126)

[Table S19a. Effects of covarying for handedness on CT (Effect sizes and p-values) 61](#_Toc507597127)

[Table S19b. Effects of covarying for handedness on SA (Effect sizes and p-values) 64](#_Toc507597128)

**Table S3a. Quadratic Age Effects on CT: 22q11DS Cases vs Controls**

| **Region** | **F** | **p-value** | **FDR p-value** |
| --- | --- | --- | --- |
| **L precuneus** | 53.83 | 6.18E-13 | 2.89E-11 |
| **L cuneus** | 53.22 | 8.26E-13 | 2.89E-11 |
| **L hemisphere** | 48.26 | 8.67E-12 | 1.54E-10 |
| **R lingual gyrus** | 47.89 | 1.04E-11 | 1.54E-10 |
| **L lateral orbitofrontal cortex** | 47.77 | 1.10E-11 | 1.54E-10 |
| **L postcentral gyrus** | 46.79 | 1.76E-11 | 2.05E-10 |
| **R hemisphere** | 46.08 | 2.45E-11 | 2.37E-10 |
| **R precuneus** | 45.87 | 2.71E-11 | 2.37E-10 |
| **R cuneus** | 45.47 | 3.29E-11 | 2.56E-10 |
| **R superior frontal gyrus** | 39.64 | 5.46E-10 | 3.82E-09 |
| **L inferior parietal cortex** | 37.57 | 1.49E-09 | 9.47E-09 |
| **L lingual gyrus** | 37.12 | 1.85E-09 | 1.08E-08 |
| **R rostral middle frontal gyrus** | 36.91 | 2.05E-09 | 1.10E-08 |
| **R superior parietal cortex** | 36.26 | 2.81E-09 | 1.41E-08 |
| **R medial orbitofrontal cortex** | 35.70 | 3.71E-09 | 1.73E-08 |
| **R lateral orbitofrontal cortex** | 35.13 | 4.88E-09 | 2.13E-08 |
| **L superior parietal cortex** | 34.71 | 5.99E-09 | 2.34E-08 |
| **L medial orbitofrontal cortex** | 34.70 | 6.02E-09 | 2.34E-08 |
| **L rostral middle frontal gyrus** | 34.19 | 7.72E-09 | 2.84E-08 |
| **L supramarginal gyrus** | 31.99 | 2.28E-08 | 7.97E-08 |
| **R inferior parietal cortex** | 31.26 | 3.26E-08 | 1.09E-07 |
| **L lateral occipital cortex** | 29.89 | 6.39E-08 | 2.03E-07 |
| **R paracentral lobule** | 26.77 | 3.01E-07 | 9.15E-07 |
| **R postcentral gyrus** | 25.82 | 4.84E-07 | 1.41E-06 |
| **L superior frontal gyrus** | 24.98 | 7.36E-07 | 2.06E-06 |
| **L paracentral lobule** | 24.07 | 1.16E-06 | 3.12E-06 |
| **L fusiform gyrus** | 23.66 | 1.42E-06 | 3.69E-06 |
| **L insula** | 22.71 | 2.30E-06 | 5.76E-06 |
| **L pars orbitalis** | 22.07 | 3.18E-06 | 7.67E-06 |
| **L pericalcarine cortex** | 21.07 | 5.27E-06 | 1.23E-05 |
| **R fusiform gyrus** | 20.48 | 7.09E-06 | 1.57E-05 |
| **L posterior cingulate** | 20.46 | 7.17E-06 | 1.57E-05 |
| **R lateral occipital cortex** | 19.87 | 9.67E-06 | 2.05E-05 |
| **L pars triangularis** | 19.78 | 1.02E-05 | 2.09E-05 |
| **R supramarginal gyrus** | 19.11 | 1.42E-05 | 2.85E-05 |
| **L banks of STS** | 18.41 | 2.04E-05 | 3.96E-05 |
| **R middle temporal gyrus** | 17.39 | 3.44E-05 | 6.51E-05 |
| **L transverse temporal gyrus** | 17.31 | 3.58E-05 | 6.59E-05 |
| **R pars triangularis** | 16.58 | 5.21E-05 | 9.35E-05 |
| **R STG** | 16.40 | 5.71E-05 | 9.99E-05 |
| **R pericalcarine cortex** | 15.98 | 7.09E-05 | 1.21E-04 |
| **L inferior temporal gyrus** | 15.89 | 7.42E-05 | 1.24E-04 |
| **R banks of STS** | 15.73 | 8.05E-05 | 1.31E-04 |
| **R inferior temporal gyrus** | 15.53 | 8.96E-05 | 1.42E-04 |
| **R insula** | 15.17 | 1.08E-04 | 1.68E-04 |
| **R pars orbitalis** | 15.02 | 1.17E-04 | 1.78E-04 |
| **L isthmus cingulate** | 12.64 | 4.03E-04 | 6.00E-04 |
| **R posterior cingulate** | 11.71 | 6.59E-04 | 9.60E-04 |
| **R rostral anterior cingulate** | 11.54 | 7.19E-04 | 1.03E-03 |
| **R caudal middle frontal gyrus** | 10.77 | 1.08E-03 | 1.51E-03 |
| **R transverse temporal gyrus** | 9.97 | 1.66E-03 | 2.28E-03 |
| **L pars opercularis** | 9.86 | 1.76E-03 | 2.37E-03 |
| **R frontal pole** | 9.82 | 1.80E-03 | 2.37E-03 |
| **R caudal anterior cingulate** | 9.57 | 2.06E-03 | 2.67E-03 |
| **R pars opercularis** | 9.50 | 2.13E-03 | 2.72E-03 |
| **L caudal middle frontal gyrus** | 8.88 | 2.98E-03 | 3.73E-03 |
| **L STG** | 8.53 | 3.61E-03 | 4.44E-03 |
| **L middle temporal gyrus** | 7.96 | 4.93E-03 | 5.95E-03 |
| **L rostral anterior cingulate** | 7.05 | 8.10E-03 | 9.61E-03 |
| **L caudal anterior cingulate** | 6.26 | 1.25E-02 | 1.46E-02 |
| **R isthmus cingulate** | 5.90 | 1.54E-02 | 1.77E-02 |
| **L frontal pole** | 5.64 | 1.78E-02 | 2.01E-02 |
| **L entorhinal cortex** | 1.76 | 1.86E-01 | 2.06E-01 |
| **R entorhinal cortex** | 1.69 | 1.94E-01 | 2.12E-01 |
| **L precentral gyrus** | 0.90 | 3.42E-01 | 3.68E-01 |
| **R precentral gyrus** | 0.63 | 4.28E-01 | 4.54E-01 |
| **L parahippocampal gyrus** | 0.58 | 4.45E-01 | 4.65E-01 |
| **R temporal pole** | 0.36 | 5.46E-01 | 5.62E-01 |
| **R parahippocampal gyrus** | 0.09 | 7.63E-01 | 7.74E-01 |
| **L temporal pole** | 0.01 | 9.07E-01 | 9.07E-01 |

**Table S3a.** Quadratic Age Effects on Cortical Thickness: 22q11DS Cases vs Controls. The following two models 1) CT ~ Group + Site + Sex + Age, and 2) CT ~ Group + Site + Sex + Age + Age^2^, were compared using F-tests. Results showed that for the majority of regions, a significantly increased proportion of variance can be accounted for by including the quadratic term of age in the models.

**Table S3b. Quadratic Age Effects on SA: 22q11DS Cases vs Controls**

| **Region** | **F** | **p-value** | **FDR p-value** |
| --- | --- | --- | --- |
| **R inferior temporal gyrus** | 12.45 | 0.0004 | 0.0210 |
| **L lateral orbitofrontal cortex** | 10.97 | 0.0010 | 0.0210 |
| **R pericalcarine cortex** | 10.75 | 0.0011 | 0.0210 |
| **L pericalcarine cortex** | 10.57 | 0.0012 | 0.0210 |
| **L cuneus** | 9.62 | 0.0020 | 0.0246 |
| **L pars orbitalis** | 9.52 | 0.0021 | 0.0246 |
| **L inferior temporal gyrus** | 8.66 | 0.0034 | 0.0336 |
| **R lateral orbitofrontal cortex** | 7.48 | 0.0064 | 0.0531 |
| **R lingual gyrus** | 7.36 | 0.0068 | 0.0531 |
| **R middle temporal gyrus** | 5.97 | 0.0148 | 0.0930 |
| **R pars opercularis** | 5.96 | 0.0149 | 0.0930 |
| **R cuneus** | 5.84 | 0.0159 | 0.0930 |
| **L lingual gyrus** | 5.60 | 0.0183 | 0.0984 |
| **L pars triangularis** | 5.19 | 0.0231 | 0.1062 |
| **R rostral anterior cingulate** | 5.14 | 0.0237 | 0.1062 |
| **R parahippocampal gyrus** | 5.10 | 0.0243 | 0.1062 |
| **L rostral anterior cingulate** | 4.38 | 0.0368 | 0.1455 |
| **L middle temporal gyrus** | 4.35 | 0.0374 | 0.1455 |
| **R hemisphere** | 4.24 | 0.0399 | 0.1470 |
| **L medial orbitofrontal cortex** | 4.10 | 0.0434 | 0.1518 |
| **L hemisphere** | 4.00 | 0.0458 | 0.1527 |
| **R pars triangularis** | 3.68 | 0.0555 | 0.1723 |
| **R medial orbitofrontal cortex** | 3.65 | 0.0566 | 0.1723 |
| **L supramarginal gyrus** | 3.45 | 0.0639 | 0.1863 |
| **R fusiform gyrus** | 3.18 | 0.0750 | 0.2058 |
| **L fusiform gyrus** | 3.06 | 0.0808 | 0.2058 |
| **L frontal pole** | 2.99 | 0.0844 | 0.2058 |
| **L parahippocampal gyrus** | 2.97 | 0.0851 | 0.2058 |
| **L rostral middle frontal gyrus** | 2.97 | 0.0853 | 0.2058 |
| **R temporal pole** | 2.80 | 0.0948 | 0.2164 |
| **L superior frontal gyrus** | 2.77 | 0.0967 | 0.2164 |
| **R superior frontal gyrus** | 2.71 | 0.1001 | 0.2164 |
| **R caudal anterior cingulate** | 2.68 | 0.1020 | 0.2164 |
| **R insula** | 2.62 | 0.1057 | 0.2176 |
| **R STG** | 2.43 | 0.1192 | 0.2385 |
| **L temporal pole** | 2.33 | 0.1272 | 0.2474 |
| **L insula** | 2.14 | 0.1442 | 0.2729 |
| **L precentral gyrus** | 1.87 | 0.1723 | 0.3174 |
| **L lateral occipital cortex** | 1.72 | 0.1900 | 0.3409 |
| **R rostral middle frontal gyrus** | 1.68 | 0.1955 | 0.3421 |
| **R lateral occipital cortex** | 1.62 | 0.2031 | 0.3446 |
| **L pars opercularis** | 1.60 | 0.2067 | 0.3446 |
| **R transverse temporal gyrus** | 1.52 | 0.2182 | 0.3552 |
| **R caudal middle frontal gyrus** | 1.46 | 0.2281 | 0.3630 |
| **L entorhinal cortex** | 1.20 | 0.2728 | 0.4121 |
| **L postcentral gyrus** | 1.19 | 0.2757 | 0.4121 |
| **R frontal pole** | 1.19 | 0.2767 | 0.4121 |
| **R banks of STS** | 1.00 | 0.3181 | 0.4639 |
| **L STG** | 0.88 | 0.3487 | 0.4982 |
| **L precuneus** | 0.83 | 0.3628 | 0.5079 |
| **L banks of STS** | 0.60 | 0.4392 | 0.6028 |
| **R supramarginal gyrus** | 0.54 | 0.4630 | 0.6233 |
| **R superior parietal cortex** | 0.48 | 0.4866 | 0.6427 |
| **R precentral gyrus** | 0.45 | 0.5005 | 0.6488 |
| **L transverse temporal gyrus** | 0.37 | 0.5409 | 0.6695 |
| **R postcentral gyrus** | 0.37 | 0.5448 | 0.6695 |
| **L caudal middle frontal gyrus** | 0.37 | 0.5452 | 0.6695 |
| **L inferior parietal cortex** | 0.34 | 0.5603 | 0.6762 |
| **R pars orbitalis** | 0.28 | 0.5945 | 0.7043 |
| **R entorhinal cortex** | 0.27 | 0.6037 | 0.7043 |
| **L superior parietal cortex** | 0.25 | 0.6185 | 0.7062 |
| **L isthmus cingulate** | 0.23 | 0.6298 | 0.7062 |
| **L posterior cingulate** | 0.22 | 0.6356 | 0.7062 |
| **R isthmus cingulate** | 0.20 | 0.6520 | 0.7131 |
| **R precuneus** | 0.13 | 0.7151 | 0.7701 |
| **R inferior parietal cortex** | 0.11 | 0.7372 | 0.7819 |
| **L paracentral lobule** | 0.05 | 0.8266 | 0.8509 |
| **L caudal anterior cingulate** | 0.05 | 0.8266 | 0.8509 |
| **R paracentral lobule** | 0.04 | 0.8435 | 0.8557 |
| **R posterior cingulate** | 0.00 | 0.9888 | 0.9888 |

**Table S3b**. Quadratic Age Effects on Surface Area: 22q11DS Cases vs Controls. The following two models 1) SA ~ Group + Site + Sex + Age, and 2) SA ~ Group + Site + Sex + Age + Age^2^, were compared using F-tests. Results showed that for the majority of regions, no further proportion of variance is accounted for by including the quadratic term of age in the models.

**Table S4a. Group x Sex Interactions on CT: 22q11DS Cases vs Controls**

| **Region** | **F** | **p-value** | **FDR p-value** |
| --- | --- | --- | --- |
| **L parahippocampal gyrus** | 6.72 | 0.01 | 0.68 |
| **R postcentral gyrus** | 4.34 | 0.04 | 0.87 |
| **R frontal pole** | 4.00 | 0.05 | 0.87 |
| **L pericalcarine cortex** | 3.49 | 0.06 | 0.87 |
| **R lateral orbitofrontal cortex** | 2.62 | 0.11 | 0.87 |
| **R temporal pole** | 2.30 | 0.13 | 0.87 |
| **R parahippocampal gyrus** | 2.27 | 0.13 | 0.87 |
| **R STG** | 2.20 | 0.14 | 0.87 |
| **L entorhinal cortex** | 1.80 | 0.18 | 0.87 |
| **R banks of STS** | 1.75 | 0.19 | 0.87 |
| **R entorhinal cortex** | 1.70 | 0.19 | 0.87 |
| **R lingual gyrus** | 1.62 | 0.20 | 0.87 |
| **R caudal middle frontal gyrus** | 1.43 | 0.23 | 0.87 |
| **R caudal anterior cingulate** | 1.29 | 0.26 | 0.87 |
| **L supramarginal gyrus** | 1.16 | 0.28 | 0.87 |
| **R lateral occipital cortex** | 1.04 | 0.31 | 0.87 |
| **L pars opercularis** | 1.00 | 0.32 | 0.87 |
| **L posterior cingulate** | 0.94 | 0.33 | 0.87 |
| **L paracentral lobule** | 0.89 | 0.35 | 0.87 |
| **L inferior parietal cortex** | 0.84 | 0.36 | 0.87 |
| **L lateral orbitofrontal cortex** | 0.84 | 0.36 | 0.87 |
| **R pericalcarine cortex** | 0.76 | 0.38 | 0.87 |
| **L lingual gyrus** | 0.75 | 0.39 | 0.87 |
| **L isthmus cingulate** | 0.72 | 0.39 | 0.87 |
| **L pars triangularis** | 0.72 | 0.40 | 0.87 |
| **R rostral middle frontal gyrus** | 0.67 | 0.41 | 0.87 |
| **L postcentral gyrus** | 0.64 | 0.42 | 0.87 |
| **L STG** | 0.63 | 0.43 | 0.87 |
| **R superior parietal cortex** | 0.59 | 0.44 | 0.87 |
| **R isthmus cingulate** | 0.59 | 0.44 | 0.87 |
| **L caudal anterior cingulate** | 0.57 | 0.45 | 0.87 |
| **L banks of STS** | 0.57 | 0.45 | 0.87 |
| **L superior frontal gyrus** | 0.55 | 0.46 | 0.87 |
| **R paracentral lobule** | 0.55 | 0.46 | 0.87 |
| **R precentral gyrus** | 0.54 | 0.46 | 0.87 |
| **R inferior parietal cortex** | 0.47 | 0.49 | 0.87 |
| **R middle temporal gyrus** | 0.45 | 0.50 | 0.87 |
| **L pars orbitalis** | 0.43 | 0.51 | 0.87 |
| **R transverse temporal gyrus** | 0.42 | 0.52 | 0.87 |
| **R pars triangularis** | 0.41 | 0.52 | 0.87 |
| **R inferior temporal gyrus** | 0.39 | 0.53 | 0.87 |
| **R pars orbitalis** | 0.36 | 0.55 | 0.87 |
| **R cuneus** | 0.30 | 0.58 | 0.87 |
| **L rostral anterior cingulate** | 0.30 | 0.58 | 0.87 |
| **L cuneus** | 0.30 | 0.59 | 0.87 |
| **R rostral anterior cingulate** | 0.29 | 0.59 | 0.87 |
| **L frontal pole** | 0.29 | 0.59 | 0.87 |
| **R insula** | 0.28 | 0.60 | 0.87 |
| **R supramarginal gyrus** | 0.19 | 0.66 | 0.92 |
| **L superior parietal cortex** | 0.19 | 0.66 | 0.92 |
| **L transverse temporal gyrus** | 0.17 | 0.68 | 0.92 |
| **R superior frontal gyrus** | 0.13 | 0.72 | 0.92 |
| **R medial orbitofrontal cortex** | 0.12 | 0.73 | 0.92 |
| **L temporal pole** | 0.11 | 0.74 | 0.92 |
| **L lateral occipital cortex** | 0.11 | 0.74 | 0.92 |
| **L fusiform gyrus** | 0.09 | 0.76 | 0.92 |
| **L medial orbitofrontal cortex** | 0.08 | 0.78 | 0.92 |
| **L rostral middle frontal gyrus** | 0.08 | 0.78 | 0.92 |
| **R pars opercularis** | 0.06 | 0.81 | 0.92 |
| **L precuneus** | 0.05 | 0.82 | 0.92 |
| **L middle temporal gyrus** | 0.05 | 0.82 | 0.92 |
| **L insula** | 0.05 | 0.82 | 0.92 |
| **R fusiform gyrus** | 0.05 | 0.83 | 0.92 |
| **R posterior cingulate** | 0.04 | 0.84 | 0.92 |
| **L precentral gyrus** | 0.03 | 0.85 | 0.92 |
| **L caudal middle frontal gyrus** | 0.01 | 0.91 | 0.96 |
| **R precuneus** | 0.01 | 0.94 | 0.97 |
| **L inferior temporal gyrus** | 0.00 | 0.96 | 0.97 |
| **L hemisphere** | 0.00 | 0.96 | 0.97 |
| **R hemisphere** | 0.00 | 0.97 | 0.97 |

**Table S4a**. Group x Sex Interactions on Cortical Thickness: 22q11DS Cases vs Controls. The following two models 1) CT ~ Group + Site + Sex + Age, and 2) CT ~ Group + Site + Sex + Age + Group * Sex, were compared using F-tests. Results showed that for the majority of regions, no further proportion of variance is accounted for by including the interaction term in the models.

**Table S4b. Group x Sex Interactions on SA: 22q11DS Cases vs Controls**

| **Region** | **F** | **p-value** | **FDR p-value** |
| --- | --- | --- | --- |
| **L cuneus** | 16.17 | 0.0001 | 0.0023 |
| **R cuneus** | 16.17 | 0.0001 | 0.0023 |
| **R lingual gyrus** | 13.66 | 0.0002 | 0.0055 |
| **R pericalcarine cortex** | 9.87 | 0.0018 | 0.0307 |
| **L precuneus** | 8.31 | 0.0041 | 0.0483 |
| **L lateral occipital cortex** | 8.28 | 0.0041 | 0.0483 |
| **R superior parietal cortex** | 7.77 | 0.0055 | 0.0547 |
| **R entorhinal cortex** | 5.80 | 0.0163 | 0.1423 |
| **R precuneus** | 5.43 | 0.0201 | 0.1566 |
| **R lateral occipital cortex** | 4.73 | 0.0300 | 0.1870 |
| **R postcentral gyrus** | 4.65 | 0.0313 | 0.1870 |
| **L inferior parietal cortex** | 4.52 | 0.0339 | 0.1870 |
| **L postcentral gyrus** | 4.48 | 0.0347 | 0.1870 |
| **R hemisphere** | 4.28 | 0.0389 | 0.1946 |
| **R caudal anterior cingulate** | 3.82 | 0.0510 | 0.2379 |
| **L pericalcarine cortex** | 3.29 | 0.0703 | 0.2897 |
| **L hemisphere** | 3.29 | 0.0703 | 0.2897 |
| **L fusiform gyrus** | 3.12 | 0.0780 | 0.3033 |
| **L pars orbitalis** | 2.71 | 0.1000 | 0.3573 |
| **R fusiform gyrus** | 2.64 | 0.1048 | 0.3573 |
| **R inferior parietal cortex** | 2.59 | 0.1078 | 0.3573 |
| **L lingual gyrus** | 2.53 | 0.1123 | 0.3573 |
| **L entorhinal cortex** | 2.39 | 0.1224 | 0.3724 |
| **L lateral orbitofrontal cortex** | 2.09 | 0.1485 | 0.3985 |
| **L temporal pole** | 2.09 | 0.1486 | 0.3985 |
| **R inferior temporal gyrus** | 2.06 | 0.1518 | 0.3985 |
| **R rostral anterior cingulate** | 2.04 | 0.1537 | 0.3985 |
| **R parahippocampal gyrus** | 1.93 | 0.1656 | 0.4126 |
| **R pars orbitalis** | 1.86 | 0.1734 | 0.4126 |
| **L supramarginal gyrus** | 1.83 | 0.1768 | 0.4126 |
| **R caudal middle frontal gyrus** | 1.74 | 0.1871 | 0.4137 |
| **L caudal anterior cingulate** | 1.73 | 0.1891 | 0.4137 |
| **R paracentral lobule** | 1.45 | 0.2282 | 0.4605 |
| **L pars triangularis** | 1.37 | 0.2429 | 0.4605 |
| **L insula** | 1.32 | 0.2510 | 0.4605 |
| **R middle temporal gyrus** | 1.31 | 0.2535 | 0.4605 |
| **L precentral gyrus** | 1.30 | 0.2538 | 0.4605 |
| **L pars opercularis** | 1.30 | 0.2550 | 0.4605 |
| **L inferior temporal gyrus** | 1.29 | 0.2566 | 0.4605 |
| **L rostral anterior cingulate** | 1.05 | 0.3054 | 0.5344 |
| **R medial orbitofrontal cortex** | 0.96 | 0.3276 | 0.5516 |
| **R supramarginal gyrus** | 0.95 | 0.3309 | 0.5516 |
| **R pars opercularis** | 0.91 | 0.3415 | 0.5559 |
| **R precentral gyrus** | 0.82 | 0.3656 | 0.5817 |
| **L superior parietal cortex** | 0.75 | 0.3882 | 0.6032 |
| **L middle temporal gyrus** | 0.72 | 0.3964 | 0.6032 |
| **L paracentral lobule** | 0.63 | 0.4267 | 0.6349 |
| **R temporal pole** | 0.61 | 0.4353 | 0.6349 |
| **L superior frontal gyrus** | 0.56 | 0.4545 | 0.6493 |
| **R pars triangularis** | 0.53 | 0.4664 | 0.6529 |
| **L posterior cingulate** | 0.51 | 0.4774 | 0.6553 |
| **R transverse temporal gyrus** | 0.45 | 0.5035 | 0.6630 |
| **R frontal pole** | 0.44 | 0.5051 | 0.6630 |
| **R superior frontal gyrus** | 0.43 | 0.5115 | 0.6630 |
| **L parahippocampal gyrus** | 0.40 | 0.5281 | 0.6673 |
| **R posterior cingulate** | 0.39 | 0.5338 | 0.6673 |
| **R banks of STS** | 0.35 | 0.5546 | 0.6810 |
| **L isthmus cingulate** | 0.31 | 0.5798 | 0.6998 |
| **R lateral orbitofrontal cortex** | 0.28 | 0.5965 | 0.7077 |
| **L rostral middle frontal gyrus** | 0.25 | 0.6149 | 0.7174 |
| **L frontal pole** | 0.23 | 0.6293 | 0.7198 |
| **L medial orbitofrontal cortex** | 0.22 | 0.6419 | 0.7198 |
| **L caudal middle frontal gyrus** | 0.21 | 0.6478 | 0.7198 |
| **R isthmus cingulate** | 0.16 | 0.6925 | 0.7575 |
| **R STG** | 0.12 | 0.7269 | 0.7703 |
| **R insula** | 0.12 | 0.7321 | 0.7703 |
| **R rostral middle frontal gyrus** | 0.11 | 0.7373 | 0.7703 |
| **L banks of STS** | 0.06 | 0.8094 | 0.8332 |
| **L transverse temporal gyrus** | 0.01 | 0.9336 | 0.9471 |
| **L STG** | 0.00 | 0.9836 | 0.9836 |

**Table S4b.** Group x Sex Interactions on Surface Area: 22q11DS Cases vs Controls. The following two models 1) SA ~ Group + Site + Sex + Age, and 2) SA ~ Group + Site + Sex + Age + Diagnosis * Sex, were compared using F-tests. Results showed that for the majority of regions, no further proportion of variance is accounted for by including the interaction term in the models.

**Table S5a. Group x Age Interactions on CT: 22q11DS Cases vs Controls**

| **Region** | **F** | **p-value** | **FDR p-value** |
| --- | --- | --- | --- |
| **L cuneus** | 14.23 | 0.0002 | 0.0123 |
| **L superior parietal cortex** | 8.44 | 0.0038 | 0.1325 |
| **R postcentral gyrus** | 7.13 | 0.0078 | 0.1629 |
| **L inferior parietal cortex** | 6.80 | 0.0093 | 0.1629 |
| **R cuneus** | 6.21 | 0.0129 | 0.1811 |
| **L postcentral gyrus** | 5.71 | 0.0171 | 0.1998 |
| **L lateral occipital cortex** | 5.18 | 0.0232 | 0.2284 |
| **L precuneus** | 4.97 | 0.0261 | 0.2284 |
| **R pars opercularis** | 4.13 | 0.0424 | 0.3189 |
| **R isthmus cingulate** | 3.87 | 0.0496 | 0.3189 |
| **R pericalcarine cortex** | 3.85 | 0.0501 | 0.3189 |
| **R superior parietal cortex** | 3.18 | 0.0748 | 0.4299 |
| **L pericalcarine cortex** | 3.06 | 0.0805 | 0.4299 |
| **R paracentral lobule** | 2.96 | 0.0860 | 0.4299 |
| **R insula** | 2.78 | 0.0959 | 0.4474 |
| **L insula** | 2.34 | 0.1267 | 0.5484 |
| **R precuneus** | 2.26 | 0.1332 | 0.5484 |
| **R temporal pole** | 2.17 | 0.1416 | 0.5507 |
| **R STG** | 1.82 | 0.1772 | 0.6530 |
| **R lateral occipital cortex** | 1.61 | 0.2047 | 0.6936 |
| **L hemisphere** | 1.55 | 0.2131 | 0.6936 |
| **L paracentral lobule** | 1.40 | 0.2367 | 0.6936 |
| **R lateral orbitofrontal cortex** | 1.35 | 0.2461 | 0.6936 |
| **R lingual gyrus** | 1.34 | 0.2478 | 0.6936 |
| **R supramarginal gyrus** | 1.32 | 0.2508 | 0.6936 |
| **L isthmus cingulate** | 1.21 | 0.2715 | 0.6936 |
| **L supramarginal gyrus** | 1.19 | 0.2763 | 0.6936 |
| **L inferior temporal gyrus** | 1.14 | 0.2859 | 0.6936 |
| **L STG** | 1.06 | 0.3025 | 0.6936 |
| **L medial orbitofrontal cortex** | 1.05 | 0.3066 | 0.6936 |
| **L superior frontal gyrus** | 1.04 | 0.3072 | 0.6936 |
| **R posterior cingulate** | 0.63 | 0.4289 | 0.9030 |
| **R rostral middle frontal gyrus** | 0.55 | 0.4592 | 0.9030 |
| **R precentral gyrus** | 0.51 | 0.4761 | 0.9030 |
| **L banks of STS** | 0.50 | 0.4777 | 0.9030 |
| **R rostral anterior cingulate** | 0.49 | 0.4844 | 0.9030 |
| **R hemisphere** | 0.46 | 0.4991 | 0.9030 |
| **L pars opercularis** | 0.44 | 0.5082 | 0.9030 |
| **R caudal anterior cingulate** | 0.40 | 0.5262 | 0.9030 |
| **L lingual gyrus** | 0.40 | 0.5269 | 0.9030 |
| **L fusiform gyrus** | 0.35 | 0.5518 | 0.9030 |
| **L precentral gyrus** | 0.29 | 0.5890 | 0.9030 |
| **L frontal pole** | 0.28 | 0.5967 | 0.9030 |
| **L lateral orbitofrontal cortex** | 0.27 | 0.6021 | 0.9030 |
| **L temporal pole** | 0.26 | 0.6098 | 0.9030 |
| **R superior frontal gyrus** | 0.26 | 0.6101 | 0.9030 |
| **R caudal middle frontal gyrus** | 0.26 | 0.6104 | 0.9030 |
| **R inferior parietal cortex** | 0.25 | 0.6192 | 0.9030 |
| **R transverse temporal gyrus** | 0.22 | 0.6392 | 0.9131 |
| **L caudal anterior cingulate** | 0.20 | 0.6542 | 0.9159 |
| **L pars orbitalis** | 0.16 | 0.6872 | 0.9283 |
| **R inferior temporal gyrus** | 0.13 | 0.7183 | 0.9283 |
| **R pars triangularis** | 0.13 | 0.7231 | 0.9283 |
| **R entorhinal cortex** | 0.13 | 0.7232 | 0.9283 |
| **L pars triangularis** | 0.10 | 0.7556 | 0.9283 |
| **R fusiform gyrus** | 0.09 | 0.7619 | 0.9283 |
| **L transverse temporal gyrus** | 0.09 | 0.7644 | 0.9283 |
| **R banks of STS** | 0.08 | 0.7811 | 0.9283 |
| **L rostral anterior cingulate** | 0.07 | 0.7944 | 0.9283 |
| **L caudal middle frontal gyrus** | 0.07 | 0.7957 | 0.9283 |
| **R parahippocampal gyrus** | 0.04 | 0.8413 | 0.9654 |
| **R medial orbitofrontal cortex** | 0.01 | 0.9050 | 0.9825 |
| **L posterior cingulate** | 0.01 | 0.9139 | 0.9825 |
| **L rostral middle frontal gyrus** | 0.01 | 0.9228 | 0.9825 |
| **L middle temporal gyrus** | 0.01 | 0.9228 | 0.9825 |
| **L entorhinal cortex** | 0.01 | 0.9263 | 0.9825 |
| **R pars orbitalis** | 0.01 | 0.9434 | 0.9856 |
| **R frontal pole** | 0.00 | 0.9715 | 0.9905 |
| **R middle temporal gyrus** | 0.00 | 0.9763 | 0.9905 |
| **L parahippocampal gyrus** | 0.00 | 0.9929 | 0.9929 |

**Table S5a.** Group x Age Interactions on Cortical Thickness: 22q11DS Cases vs Controls. The following two models 1) CT ~ Group + Site + Sex + Age + Age^2^, and 2) CT ~ Group + Site + Sex + Age + Age^2^ + Group * Age, were compared using F-tests. Results showed that for the majority of regions, no further proportion of variance is accounted for by including the interaction term in the models.

**Table S5b. Group x Age Interactions on SA: 22q11DS Cases vs Controls**

| **Region** | **F** | **p-value** | **FDR p-value** |
| --- | --- | --- | --- |
| **L precuneus** | 4.06 | 0.04 | 0.89 |
| **R cuneus** | 4.01 | 0.05 | 0.89 |
| **L inferior parietal cortex** | 3.90 | 0.05 | 0.89 |
| **R parahippocampal gyrus** | 3.57 | 0.06 | 0.89 |
| **L postcentral gyrus** | 3.37 | 0.07 | 0.89 |
| **L superior parietal cortex** | 2.81 | 0.09 | 0.89 |
| **L STG** | 2.64 | 0.10 | 0.89 |
| **R caudal middle frontal gyrus** | 2.41 | 0.12 | 0.89 |
| **L insula** | 2.25 | 0.13 | 0.89 |
| **L lingual gyrus** | 2.23 | 0.14 | 0.89 |
| **L hemisphere** | 1.94 | 0.16 | 0.89 |
| **L parahippocampal gyrus** | 1.76 | 0.19 | 0.89 |
| **R insula** | 1.61 | 0.21 | 0.89 |
| **L cuneus** | 1.59 | 0.21 | 0.89 |
| **L rostral middle frontal gyrus** | 1.51 | 0.22 | 0.89 |
| **R precuneus** | 1.30 | 0.25 | 0.89 |
| **L precentral gyrus** | 1.27 | 0.26 | 0.89 |
| **L banks of STS** | 1.21 | 0.27 | 0.89 |
| **R inferior parietal cortex** | 1.07 | 0.30 | 0.89 |
| **L pericalcarine cortex** | 1.04 | 0.31 | 0.89 |
| **R pericalcarine cortex** | 1.03 | 0.31 | 0.89 |
| **R paracentral lobule** | 0.97 | 0.33 | 0.89 |
| **R postcentral gyrus** | 0.94 | 0.33 | 0.89 |
| **L entorhinal cortex** | 0.90 | 0.34 | 0.89 |
| **R rostral middle frontal gyrus** | 0.90 | 0.34 | 0.89 |
| **L supramarginal gyrus** | 0.88 | 0.35 | 0.89 |
| **L medial orbitofrontal cortex** | 0.87 | 0.35 | 0.89 |
| **R lateral occipital cortex** | 0.80 | 0.37 | 0.89 |
| **L inferior temporal gyrus** | 0.77 | 0.38 | 0.89 |
| **R medial orbitofrontal cortex** | 0.71 | 0.40 | 0.89 |
| **R fusiform gyrus** | 0.69 | 0.41 | 0.89 |
| **R frontal pole** | 0.68 | 0.41 | 0.89 |
| **R hemisphere** | 0.51 | 0.48 | 0.95 |
| **R caudal anterior cingulate** | 0.48 | 0.49 | 0.95 |
| **R supramarginal gyrus** | 0.47 | 0.49 | 0.95 |
| **R pars orbitalis** | 0.40 | 0.53 | 0.95 |
| **R lingual gyrus** | 0.37 | 0.54 | 0.95 |
| **L lateral occipital cortex** | 0.36 | 0.55 | 0.95 |
| **L caudal middle frontal gyrus** | 0.33 | 0.57 | 0.95 |
| **R precentral gyrus** | 0.32 | 0.57 | 0.95 |
| **R temporal pole** | 0.27 | 0.60 | 0.95 |
| **R isthmus cingulate** | 0.27 | 0.61 | 0.95 |
| **L posterior cingulate** | 0.25 | 0.62 | 0.95 |
| **L pars triangularis** | 0.22 | 0.64 | 0.95 |
| **R pars triangularis** | 0.21 | 0.65 | 0.95 |
| **R superior parietal cortex** | 0.21 | 0.65 | 0.95 |
| **L middle temporal gyrus** | 0.21 | 0.65 | 0.95 |
| **R entorhinal cortex** | 0.20 | 0.65 | 0.95 |
| **R rostral anterior cingulate** | 0.18 | 0.67 | 0.95 |
| **L fusiform gyrus** | 0.15 | 0.70 | 0.98 |
| **L rostral anterior cingulate** | 0.13 | 0.72 | 0.98 |
| **L caudal anterior cingulate** | 0.12 | 0.73 | 0.99 |
| **R transverse temporal gyrus** | 0.10 | 0.75 | 0.99 |
| **L superior frontal gyrus** | 0.07 | 0.79 | 0.99 |
| **R pars opercularis** | 0.06 | 0.81 | 0.99 |
| **L lateral orbitofrontal cortex** | 0.04 | 0.84 | 0.99 |
| **L transverse temporal gyrus** | 0.04 | 0.85 | 0.99 |
| **L pars orbitalis** | 0.03 | 0.85 | 0.99 |
| **R lateral orbitofrontal cortex** | 0.03 | 0.87 | 0.99 |
| **L temporal pole** | 0.02 | 0.89 | 0.99 |
| **R middle temporal gyrus** | 0.01 | 0.91 | 0.99 |
| **L frontal pole** | 0.01 | 0.94 | 0.99 |
| **R inferior temporal gyrus** | 0.00 | 0.94 | 0.99 |
| **L paracentral lobule** | 0.00 | 0.95 | 0.99 |
| **R STG** | 0.00 | 0.96 | 0.99 |
| **R superior frontal gyrus** | 0.00 | 0.97 | 0.99 |
| **R posterior cingulate** | 0.00 | 0.97 | 0.99 |
| **L isthmus cingulate** | 0.00 | 0.99 | 0.99 |
| **L pars opercularis** | 0.00 | 0.99 | 0.99 |
| **R banks of STS** | 0.00 | 0.99 | 0.99 |

**Table S5b.** Group x Age Interactions on Surface Area: 22q11DS Cases vs Controls. The following two models 1) SA ~ Group + Site + Sex + Age + ICV, and 2) CT ~ Group + Site + Sex + Age + ICV + Group * Age, were compared using F-tests. Results showed that for the majority of regions, no further proportion of variance is accounted for by including the interaction term in the models.

# **Table S6a. Linear vs Quadratic Age Effects on Cortical Thickness: 22q11DS- Psychosis vs. No Psychosis**

| Region | p-values | FDR p-values |
| --- | --- | --- |
| L cuneus | 0.0065 | 0.4136 |
| R medial orbitofrontal cortex | 0.0212 | 0.4136 |
| R posterior cingulate | 0.0152 | 0.4136 |
| R rostral middle frontal gyrus | 0.0236 | 0.4136 |
| L posterior cingulate | 0.0672 | 0.5229 |
| L temporal pole | 0.0479 | 0.5229 |
| R caudal anterior cingulate | 0.0653 | 0.5229 |
| R rostral anterior cingulate | 0.0475 | 0.5229 |
| R temporal pole | 0.0544 | 0.5229 |
| L supramarginal gyrus | 0.0849 | 0.5944 |
| L insula | 0.0983 | 0.6113 |
| R caudal middle frontal gyrus | 0.1048 | 0.6113 |
| L pars triangularis | 0.1308 | 0.6304 |
| R lingual gyrus | 0.1351 | 0.6304 |
| R precentral gyrus | 0.1210 | 0.6304 |
| L parahippocampal gyrus | 0.1564 | 0.6440 |
| R superior frontal gyrus | 0.1501 | 0.6440 |
| L rostral middle frontal gyrus | 0.1760 | 0.6844 |
| L frontal pole | 0.2125 | 0.7437 |
| R isthmus cingulate | 0.2088 | 0.7437 |
| L inferior parietal cortex | 0.2261 | 0.7537 |
| L lateral occipital cortex | 0.3630 | 0.7709 |
| L lateral orbitofrontal cortex | 0.2803 | 0.7709 |
| L paracentral lobule | 0.2959 | 0.7709 |
| L pars orbitalis | 0.3854 | 0.7709 |
| L precentral gyrus | 0.2976 | 0.7709 |
| L precuneus | 0.3227 | 0.7709 |
| L transverse temporal gyrus | 0.2692 | 0.7709 |
| R parahippocampal gyrus | 0.2725 | 0.7709 |
| R pars opercularis | 0.3777 | 0.7709 |
| R pericalcarine cortex | 0.2465 | 0.7709 |
| R postcentral gyrus | 0.3699 | 0.7709 |
| R precuneus | 0.3222 | 0.7709 |
| R transverse temporal gyrus | 0.3737 | 0.7709 |
| R hemisphere | 0.3592 | 0.7709 |
| L inferior temporal gyrus | 0.4413 | 0.7906 |
| R cuneus | 0.4518 | 0.7906 |
| R inferior temporal gyrus | 0.4476 | 0.7906 |
| R supramarginal gyrus | 0.4265 | 0.7906 |
| L hemisphere | 0.4178 | 0.7906 |
| R lateral orbitofrontal cortex | 0.4791 | 0.8180 |
| L banks of STS | 0.4947 | 0.8244 |
| L postcentral gyrus | 0.5381 | 0.8760 |
| L pars opercularis | 0.5811 | 0.8842 |
| R lateral occipital cortex | 0.5773 | 0.8842 |
| R superior parietal cortex | 0.5759 | 0.8842 |
| L medial orbitofrontal cortex | 0.6395 | 0.9136 |
| R pars orbitalis | 0.6298 | 0.9136 |
| R pars triangularis | 0.6156 | 0.9136 |
| R paracentral lobule | 0.6801 | 0.9521 |
| L caudal anterior cingulate | 0.7126 | 0.9778 |
| L entorhinal cortex | 0.7264 | 0.9778 |
| L lingual gyrus | 0.7562 | 0.9802 |
| L superior frontal gyrus | 0.7480 | 0.9802 |
| L caudal middle frontal gyrus | 0.7922 | 0.9849 |
| L fusiform gyrus | 0.8809 | 0.9849 |
| L isthmus cingulate | 0.8569 | 0.9849 |
| L middle temporal gyrus | 0.9415 | 0.9849 |
| L rostral anterior cingulate | 0.9568 | 0.9849 |
| L superior parietal cortex | 0.8323 | 0.9849 |
| L STG | 0.9203 | 0.9849 |
| R entorhinal cortex | 0.9081 | 0.9849 |
| R fusiform gyrus | 0.9302 | 0.9849 |
| R inferior parietal cortex | 0.9493 | 0.9849 |
| R middle temporal gyrus | 0.8007 | 0.9849 |
| R STG | 0.8805 | 0.9849 |
| R frontal pole | 0.8730 | 0.9849 |
| R insula | 0.8249 | 0.9849 |
| L pericalcarine cortex | 0.9984 | 0.9984 |
| R banks of STS | 0.9920 | 0.9984 |

**Table S6a.** Linear vs Quadratic Age Effects on Cortical Thickness: 22q11DS- Psychosis vs. No Psychosis. The following two models 1) CT ~ Group + Site + Sex + Age, and 2) CT ~ Group + Site + Sex + Age + Age^2^, were compared using F-tests. Results showed that for all regions, no further proportion of variance is accounted for by including the quadratic term of age in the models.

# **Table S6b. Linear vs. Quadratic Age Effects on Surface Area: 22q11DS-Psychosis vs. No Psychosis**

| Region | p-values | FDR p-values |
| --- | --- | --- |
| L banks of STS | 0.96 | 0.98 |
| L caudal anterior cingulate | 0.73 | 0.95 |
| L caudal middle frontal gyrus | 0.91 | 0.98 |
| L cuneus | 0.08 | 0.85 |
| L entorhinal cortex | 0.25 | 0.85 |
| L fusiform gyrus | 0.79 | 0.95 |
| L inferior parietal cortex | 0.75 | 0.95 |
| L inferior temporal gyrus | 0.34 | 0.85 |
| L isthmus cingulate | 0.18 | 0.85 |
| L lateral occipital cortex | 0.61 | 0.88 |
| L lateral orbitofrontal cortex | 0.46 | 0.86 |
| L lingual gyrus | 0.06 | 0.85 |
| L medial orbitofrontal cortex | 0.19 | 0.85 |
| L middle temporal gyrus | 0.33 | 0.85 |
| L parahippocampal gyrus | 0.26 | 0.85 |
| L paracentral lobule | 0.06 | 0.85 |
| L pars opercularis | 0.06 | 0.85 |
| L pars orbitalis | 0.49 | 0.86 |
| L pars triangularis | 0.47 | 0.86 |
| L pericalcarine cortex | 0.41 | 0.85 |
| L postcentral gyrus | 0.39 | 0.85 |
| L posterior cingulate | 0.60 | 0.88 |
| L precentral gyrus | 0.58 | 0.88 |
| L precuneus | 0.61 | 0.88 |
| L rostral anterior cingulate | 0.80 | 0.95 |
| L rostral middle frontal gyrus | 0.30 | 0.85 |
| L superior frontal gyrus | 0.93 | 0.98 |
| L superior parietal cortex | 0.26 | 0.85 |
| L STG | 0.23 | 0.85 |
| L supramarginal gyrus | 0.91 | 0.98 |
| L frontal pole | 0.24 | 0.85 |
| L temporal pole | 0.85 | 0.97 |
| L transverse temporal gyrus | 0.27 | 0.85 |
| L insula | 0.21 | 0.85 |
| R banks of STS | 0.36 | 0.85 |
| R caudal anterior cingulate | 0.81 | 0.95 |
| R caudal middle frontal gyrus | 0.46 | 0.86 |
| R cuneus | 0.33 | 0.85 |
| R entorhinal cortex | 0.97 | 0.98 |
| R fusiform gyrus | 0.31 | 0.85 |
| R inferior parietal cortex | 0.18 | 0.85 |
| R inferior temporal gyrus | 0.79 | 0.95 |
| R isthmus cingulate | 0.15 | 0.85 |
| R lateral occipital cortex | 0.57 | 0.88 |
| R lateral orbitofrontal cortex | 0.31 | 0.85 |
| R lingual gyrus | 0.20 | 0.85 |
| R medial orbitofrontal cortex | 0.32 | 0.85 |
| R middle temporal gyrus | 0.55 | 0.88 |
| R parahippocampal gyrus | 0.11 | 0.85 |
| R paracentral lobule | 0.80 | 0.95 |
| R pars opercularis | 0.09 | 0.85 |
| R pars orbitalis | 0.81 | 0.95 |
| R pars triangularis | 0.94 | 0.98 |
| R pericalcarine cortex | 0.38 | 0.85 |
| R postcentral gyrus | 0.99 | 0.99 |
| R posterior cingulate | 0.41 | 0.85 |
| R precentral gyrus | 0.89 | 0.98 |
| R precuneus | 0.86 | 0.97 |
| R rostral anterior cingulate | 0.41 | 0.85 |
| R rostral middle frontal gyrus | 0.55 | 0.88 |
| R superior frontal gyrus | 0.76 | 0.95 |
| R superior parietal cortex | 0.53 | 0.88 |
| R STG | 0.24 | 0.85 |
| R supramarginal gyrus | 0.69 | 0.95 |
| R frontal pole | 0.25 | 0.85 |
| R temporal pole | 0.81 | 0.95 |
| R transverse temporal gyrus | 0.46 | 0.86 |
| R insula | 0.27 | 0.85 |
| L hemisphere | 0.48 | 0.86 |
| R hemisphere | 0.61 | 0.88 |

**Table S6b**. Linear vs. Quadratic Age Effects on Surface Area: 22q11DS-Psychosis vs. No Psychosis. The following two models 1) SA ~ Group + Site + Sex + ICV + Age, and 2) SA ~ Group + Site + Sex + ICV + Age + Age^2^, were compared using F-tests. Results showed that for all regions, no further proportion of variance is accounted for by including the quadratic term of age in the models.

# **Table S7a. Linear vs. Quadratic Age Effects on Cortical Thickness: A-D and A-B Deletion and Controls**

| Region | p-values | FDR p-values |
| --- | --- | --- |
| L paracentral lobule | 3.32E-06 | 2.90E-05 |
| L postcentral gyrus | 2.32E-06 | 2.90E-05 |
| L precuneus | 1.88E-06 | 2.90E-05 |
| R lingual gyrus | 1.45E-06 | 2.90E-05 |
| R pars triangularis | 3.10E-06 | 2.90E-05 |
| R postcentral gyrus | 3.08E-06 | 2.90E-05 |
| L hemisphere | 2.43E-06 | 2.90E-05 |
| R hemisphere | 2.58E-06 | 2.90E-05 |
| L lateral orbitofrontal cortex | 4.66E-06 | 3.63E-05 |
| L rostral middle frontal gyrus | 5.45E-06 | 3.81E-05 |
| R precuneus | 1.02E-05 | 6.50E-05 |
| R rostral middle frontal gyrus | 1.16E-05 | 6.77E-05 |
| L pars orbitalis | 1.34E-05 | 7.23E-05 |
| L cuneus | 1.99E-05 | 9.55E-05 |
| L pars triangularis | 2.36E-05 | 9.55E-05 |
| L supramarginal gyrus | 2.23E-05 | 9.55E-05 |
| R cuneus | 2.37E-05 | 9.55E-05 |
| R inferior parietal cortex | 2.46E-05 | 9.55E-05 |
| R transverse temporal gyrus | 4.13E-05 | 1.52E-04 |
| L lateral occipital cortex | 4.85E-05 | 1.54E-04 |
| L superior parietal cortex | 4.92E-05 | 1.54E-04 |
| R caudal middle frontal gyrus | 5.07E-05 | 1.54E-04 |
| R superior parietal cortex | 4.48E-05 | 1.54E-04 |
| L inferior parietal cortex | 6.34E-05 | 1.78E-04 |
| L lingual gyrus | 6.27E-05 | 1.78E-04 |
| L frontal pole | 1.04E-04 | 2.81E-04 |
| L medial orbitofrontal cortex | 1.79E-04 | 4.65E-04 |
| R supramarginal gyrus | 2.55E-04 | 6.38E-04 |
| R superior frontal gyrus | 3.35E-04 | 8.08E-04 |
| L superior frontal gyrus | 3.82E-04 | 8.91E-04 |
| L posterior cingulate | 4.92E-04 | 1.11E-03 |
| R lateral orbitofrontal cortex | 5.67E-04 | 1.24E-03 |
| R posterior cingulate | 6.57E-04 | 1.39E-03 |
| R medial orbitofrontal cortex | 6.81E-04 | 1.40E-03 |
| L caudal middle frontal gyrus | 1.05E-03 | 2.09E-03 |
| R fusiform gyrus | 1.27E-03 | 2.47E-03 |
| R middle temporal gyrus | 1.38E-03 | 2.61E-03 |
| L pars opercularis | 2.48E-03 | 4.57E-03 |
| R banks of STS | 2.82E-03 | 5.06E-03 |
| R lateral occipital cortex | 3.34E-03 | 5.85E-03 |
| R inferior temporal gyrus | 3.90E-03 | 6.65E-03 |
| R paracentral lobule | 5.05E-03 | 8.41E-03 |
| R pars orbitalis | 5.26E-03 | 8.56E-03 |
| R pericalcarine cortex | 5.54E-03 | 8.81E-03 |
| L insula | 5.70E-03 | 8.86E-03 |
| R pars opercularis | 1.40E-02 | 2.13E-02 |
| R rostral anterior cingulate | 1.64E-02 | 2.45E-02 |
| L transverse temporal gyrus | 2.30E-02 | 3.30E-02 |
| R STG | 2.31E-02 | 3.30E-02 |
| L precentral gyrus | 2.70E-02 | 3.78E-02 |
| L fusiform gyrus | 3.07E-02 | 4.22E-02 |
| L pericalcarine cortex | 4.81E-02 | 6.48E-02 |
| R precentral gyrus | 5.27E-02 | 6.96E-02 |
| R frontal pole | 6.27E-02 | 8.12E-02 |
| L STG | 6.49E-02 | 8.26E-02 |
| R isthmus cingulate | 8.73E-02 | 1.09E-01 |
| L banks of STS | 1.26E-01 | 1.55E-01 |
| L inferior temporal gyrus | 1.54E-01 | 1.84E-01 |
| L rostral anterior cingulate | 1.55E-01 | 1.84E-01 |
| L isthmus cingulate | 2.00E-01 | 2.33E-01 |
| L parahippocampal gyrus | 3.60E-01 | 4.13E-01 |
| R insula | 3.70E-01 | 4.18E-01 |
| L temporal pole | 4.17E-01 | 4.63E-01 |
| L middle temporal gyrus | 4.94E-01 | 5.40E-01 |
| R parahippocampal gyrus | 6.73E-01 | 7.25E-01 |
| R entorhinal cortex | 6.88E-01 | 7.29E-01 |
| R temporal pole | 7.26E-01 | 7.59E-01 |
| L caudal anterior cingulate | 7.78E-01 | 7.99E-01 |
| L entorhinal cortex | 7.87E-01 | 7.99E-01 |
| R caudal anterior cingulate | 9.40E-01 | 9.40E-01 |

**Table S7a**. Linear vs. Quadratic Age Effects on Cortical Thickness: A-D and A-B Deletion and Controls. The following two models 1) CT ~ Group + Site + Sex + Age, and 2) CT ~ Group + Site + Sex + Age + Age^2^, were compared using F-tests. Results showed that for the majority of regions, a significantly increased proportion of variance can be accounted for by including the quadratic term of age in the models.

**Table S7B. Linear vs. Quadratic Age Effects on Surface Area: A-D and A-B Deletion and Controls**

| Region | p-values | FDR p-values |
| --- | --- | --- |
| R pericalcarine cortex | 0.0042 | 0.2924 |
| L pericalcarine cortex | 0.0230 | 0.8051 |
| L banks of STS | 0.2109 | 0.8989 |
| L caudal middle frontal gyrus | 0.3473 | 0.8989 |
| L cuneus | 0.2255 | 0.8989 |
| L inferior temporal gyrus | 0.2597 | 0.8989 |
| L isthmus cingulate | 0.1657 | 0.8989 |
| L lateral orbitofrontal cortex | 0.3873 | 0.8989 |
| L lingual gyrus | 0.3999 | 0.8989 |
| L medial orbitofrontal cortex | 0.4359 | 0.8989 |
| L middle temporal gyrus | 0.3427 | 0.8989 |
| L parahippocampal gyrus | 0.2313 | 0.8989 |
| L paracentral lobule | 0.5117 | 0.8989 |
| L pars opercularis | 0.4791 | 0.8989 |
| L pars orbitalis | 0.6704 | 0.8989 |
| L pars triangularis | 0.6934 | 0.8989 |
| L postcentral gyrus | 0.1661 | 0.8989 |
| L posterior cingulate | 0.6172 | 0.8989 |
| L precuneus | 0.4573 | 0.8989 |
| L rostral anterior cingulate | 0.1729 | 0.8989 |
| L superior frontal gyrus | 0.5451 | 0.8989 |
| L superior parietal cortex | 0.4032 | 0.8989 |
| L STG | 0.4908 | 0.8989 |
| L frontal pole | 0.6472 | 0.8989 |
| L temporal pole | 0.6834 | 0.8989 |
| L transverse temporal gyrus | 0.6216 | 0.8989 |
| L insula | 0.6065 | 0.8989 |
| R banks of STS | 0.3005 | 0.8989 |
| R caudal anterior cingulate | 0.6840 | 0.8989 |
| R caudal middle frontal gyrus | 0.0862 | 0.8989 |
| R cuneus | 0.0970 | 0.8989 |
| R fusiform gyrus | 0.3343 | 0.8989 |
| R inferior parietal cortex | 0.4182 | 0.8989 |
| R lateral occipital cortex | 0.5782 | 0.8989 |
| R lateral orbitofrontal cortex | 0.2123 | 0.8989 |
| R lingual gyrus | 0.6700 | 0.8989 |
| R medial orbitofrontal cortex | 0.5131 | 0.8989 |
| R middle temporal gyrus | 0.6764 | 0.8989 |
| R parahippocampal gyrus | 0.2949 | 0.8989 |
| R paracentral lobule | 0.4003 | 0.8989 |
| R pars opercularis | 0.5669 | 0.8989 |
| R pars orbitalis | 0.2888 | 0.8989 |
| R pars triangularis | 0.2373 | 0.8989 |
| R postcentral gyrus | 0.1879 | 0.8989 |
| R posterior cingulate | 0.1555 | 0.8989 |
| R precentral gyrus | 0.3624 | 0.8989 |
| R precuneus | 0.2509 | 0.8989 |
| R rostral anterior cingulate | 0.3525 | 0.8989 |
| R superior parietal cortex | 0.6906 | 0.8989 |
| R supramarginal gyrus | 0.4985 | 0.8989 |
| R frontal pole | 0.4873 | 0.8989 |
| R temporal pole | 0.0601 | 0.8989 |
| R insula | 0.4423 | 0.8989 |
| R hemisphere | 0.6127 | 0.8989 |
| L fusiform gyrus | 0.7222 | 0.9191 |
| L rostral middle frontal gyrus | 0.7366 | 0.9208 |
| L hemisphere | 0.7596 | 0.9329 |
| R STG | 0.7784 | 0.9394 |
| L caudal anterior cingulate | 0.9248 | 0.9428 |
| L entorhinal cortex | 0.8391 | 0.9428 |
| L inferior parietal cortex | 0.8511 | 0.9428 |
| L lateral occipital cortex | 0.9216 | 0.9428 |
| L precentral gyrus | 0.9293 | 0.9428 |
| L supramarginal gyrus | 0.8202 | 0.9428 |
| R entorhinal cortex | 0.9036 | 0.9428 |
| R inferior temporal gyrus | 0.8626 | 0.9428 |
| R isthmus cingulate | 0.7983 | 0.9428 |
| R rostral middle frontal gyrus | 0.8969 | 0.9428 |
| R superior frontal gyrus | 0.8720 | 0.9428 |
| R transverse temporal gyrus | 0.9445 | 0.9445 |

**Table S7b**. Linear vs. Quadratic Age Effects on Surface Area: A-D and A-B Deletion and Controls. The following two models 1) SA ~ Group + Site + Sex + ICV + Age, and 2) SA ~ Group + Site + Sex + ICV + Age + Age^2^, were compared using F-tests. Results showed that for all regions, no further proportion of variance is accounted for by including the quadratic term of age in the models.

**Table S8. Use of Psychotropic Medications in 22q11DS subjects.**

| Site | Current Typical Antipsychotics | Current Atypical Antipsychotics | Current Anticonvulsants | Current Antidepressants |
| --- | --- | --- | --- | --- |
| Cardiff | N/A | N/A | N/A | N/A |
| IoP | 0 | 5 | 0 | 3 |
| Maastricht | 0 | 11 | 1 | 5 |
| Newcastle | 0 | 3 | 2 | 3 |
| Penn | 2 | 7 | 2 | 18 |
| SUNY | 2 | 7 | 4 | 14 |
| Toronto1 | 6 | 5 | 2 | 10 |
| Toronto2 | 0 | 10 | 3 | 4 |
| UCDavis1 | 0 | 4 | 1 | 3 |
| UCDavis2 | 0 | 4 | 0 | 2 |
| UCLA1 | 1 | 1 | 1 | 6 |
| UCLA1 | 3 | 1 | 4 | 13 |
| Utrecht | 1 | 5 | 2 | 2 |

# **Table S9. Effect Sizes (cohen's *d*) for Group Differences in Global Brain Metrics across Sites**

| Site | ICV | Brain Volume | Mean Thickness | Total Surface Area |
| --- | --- | --- | --- | --- |
| Cardiff | -0.8749 | -1.0554 | 1.5791 | -1.6729 |
| IoP | -0.7090 | -1.0799 | 0.4917 | -1.2944 |
| Maastricht | -1.4831 | -1.4334 | 0.5124 | -1.5296 |
| Newcastle | -1.1532 | -1.6015 | 0.7432 | -1.7173 |
| Penn | -0.6537 | -1.0159 | 0.4725 | -1.0291 |
| SUNY | -0.6126 | -0.8610 | 0.7164 | -1.1673 |
| Toronto1 | -0.4671 | -1.0663 | 0.2692 | -1.3974 |
| UCDavis1 | -0.8574 | -1.0807 | 0.8059 | -1.2976 |
| UCDavis2 | -0.7355 | -1.2234 | 0.5875 | -1.4447 |
| UCLA1 | -0.3584 | -0.9178 | 0.6109 | -1.1193 |
| UCLA2 | -0.5026 | -1.1587 | 0.7793 | -1.3946 |

**Table S9.** Effect Sizes (Cohen's *D*) For Global Brain Metrics Across Sites. Differences in 4 global brain measures (ICV, total brain volume, mean cortical thickness, and total cortical surface area) between 22q11DS cases and controls were compared for each dataset/site separately. Consistent patterns of group differences can be observed across sites.

**Table S10a: Group Differences in CT, 22q11DS Cases vs Controls.**

| **Region** | **Cohen d** | **Std. Err.** | **95% CI** | **%Diff** | **t-value** | **p-value** | **p-FDR** |
| --- | --- | --- | --- | --- | --- | --- | --- |
| **L insula** | 0.87 | 0.08 | 0.71, 1.03 | 3.64 | 11.34 | 1.91E-27 | 1.34E-25 |
| **R rostral middle frontal gyrus** | 0.80 | 0.08 | 0.64, 0.95 | 4.01 | 10.41 | 1.13E-23 | 3.97E-22 |
| **L pericalcarine cortex** | 0.72 | 0.08 | 0.57, 0.87 | 5.59 | 9.39 | 8.68E-20 | 2.03E-18 |
| **R supramarginal gyrus** | 0.71 | 0.08 | 0.55, 0.86 | 3.28 | 9.21 | 3.79E-19 | 6.62E-18 |
| **L rostral middle frontal gyrus** | 0.70 | 0.08 | 0.54, 0.85 | 3.51 | 9.09 | 1.08E-18 | 1.52E-17 |
| **R pericalcarine cortex** | 0.68 | 0.08 | 0.52, 0.83 | 5.21 | 8.81 | 1.02E-17 | 1.19E-16 |
| **R hemisphere** | 0.65 | 0.08 | 0.49, 0.8 | 2.26 | 8.41 | 2.41E-16 | 2.41E-15 |
| **R caudal middle frontal gyrus** | 0.63 | 0.08 | 0.48, 0.78 | 3.43 | 8.23 | 9.38E-16 | 8.21E-15 |
| **R insula** | 0.63 | 0.08 | 0.48, 0.78 | 2.92 | 8.21 | 1.13E-15 | 8.75E-15 |
| **L postcentral gyrus** | 0.61 | 0.08 | 0.46, 0.77 | 3.30 | 8.01 | 4.90E-15 | 3.43E-14 |
| **L hemisphere** | 0.61 | 0.08 | 0.46, 0.76 | 2.11 | 7.95 | 7.49E-15 | 4.77E-14 |
| **R medial orbitofrontal cortex** | 0.61 | 0.08 | 0.45, 0.76 | 3.56 | 7.91 | 1.06E-14 | 6.16E-14 |
| **L supramarginal gyrus** | 0.60 | 0.08 | 0.45, 0.75 | 2.88 | 7.81 | 2.21E-14 | 1.19E-13 |
| **R cuneus** | 0.58 | 0.08 | 0.43, 0.73 | 3.39 | 7.56 | 1.32E-13 | 6.16E-13 |
| **L pars opercularis** | 0.57 | 0.08 | 0.42, 0.72 | 2.80 | 7.46 | 2.63E-13 | 1.15E-12 |
| **R paracentral lobule** | 0.56 | 0.08 | 0.41, 0.71 | 2.96 | 7.33 | 6.65E-13 | 2.74E-12 |
| **L medial orbitofrontal cortex** | 0.56 | 0.08 | 0.41, 0.71 | 3.26 | 7.30 | 7.79E-13 | 3.03E-12 |
| **L paracentral lobule** | 0.55 | 0.08 | 0.4, 0.7 | 3.06 | 7.12 | 2.65E-12 | 9.47E-12 |
| **R lingual gyrus** | 0.55 | 0.08 | 0.4, 0.7 | 2.88 | 7.12 | 2.70E-12 | 9.47E-12 |
| **R superior frontal gyrus** | 0.53 | 0.08 | 0.38, 0.68 | 2.30 | 6.89 | 1.25E-11 | 4.16E-11 |
| **L cuneus** | 0.52 | 0.08 | 0.37, 0.67 | 3.27 | 6.78 | 2.55E-11 | 8.12E-11 |
| **L caudal middle frontal gyrus** | 0.51 | 0.08 | 0.36, 0.66 | 2.57 | 6.63 | 6.79E-11 | 2.07E-10 |
| **L pars triangularis** | 0.50 | 0.08 | 0.35, 0.66 | 2.88 | 6.57 | 1.00E-10 | 2.92E-10 |
| **L superior frontal gyrus** | 0.49 | 0.08 | 0.34, 0.65 | 2.17 | 6.44 | 2.18E-10 | 5.87E-10 |
| **R precuneus** | 0.49 | 0.08 | 0.34, 0.64 | 2.15 | 6.43 | 2.42E-10 | 6.26E-10 |
| **L lateral orbitofrontal cortex** | 0.47 | 0.08 | 0.32, 0.62 | 2.44 | 6.11 | 1.71E-09 | 4.27E-09 |
| **R pars triangularis** | 0.44 | 0.08 | 0.29, 0.59 | 2.43 | 5.77 | 1.19E-08 | 2.86E-08 |
| **L pars orbitalis** | 0.44 | 0.08 | 0.29, 0.59 | 3.09 | 5.71 | 1.71E-08 | 3.99E-08 |
| **R postcentral gyrus** | 0.43 | 0.08 | 0.28, 0.58 | 2.53 | 5.60 | 3.17E-08 | 7.15E-08 |
| **R middle temporal gyrus** | 0.42 | 0.08 | 0.27, 0.57 | 1.99 | 5.52 | 4.74E-08 | 1.04E-07 |
| **R lateral orbitofrontal cortex** | 0.42 | 0.08 | 0.27, 0.57 | 2.21 | 5.46 | 6.54E-08 | 1.39E-07 |
| **L precentral gyrus** | 0.42 | 0.08 | 0.27, 0.57 | 2.12 | 5.46 | 6.74E-08 | 1.39E-07 |
| **R precentral gyrus** | 0.42 | 0.08 | 0.27, 0.57 | 2.21 | 5.45 | 7.18E-08 | 1.44E-07 |
| **L lingual gyrus** | 0.41 | 0.08 | 0.26, 0.56 | 2.06 | 5.36 | 1.12E-07 | 2.18E-07 |
| **L precuneus** | 0.37 | 0.08 | 0.22, 0.52 | 1.63 | 4.88 | 1.35E-06 | 2.55E-06 |
| **L middle temporal gyrus** | 0.37 | 0.08 | 0.22, 0.52 | 1.77 | 4.83 | 1.67E-06 | 3.07E-06 |
| **R pars orbitalis** | 0.36 | 0.08 | 0.21, 0.51 | 2.41 | 4.75 | 2.50E-06 | 4.48E-06 |
| **R pars opercularis** | 0.34 | 0.08 | 0.19, 0.49 | 1.81 | 4.46 | 9.77E-06 | 1.71E-05 |
| **R inferior temporal gyrus** | 0.34 | 0.08 | 0.19, 0.49 | 1.68 | 4.43 | 1.08E-05 | 1.85E-05 |
| **R superior parietal cortex** | 0.30 | 0.08 | 0.15, 0.45 | 1.54 | 3.90 | 1.05E-04 | 1.71E-04 |
| **L isthmus cingulate** | 0.30 | 0.08 | 0.15, 0.45 | 1.74 | 3.86 | 1.23E-04 | 1.95E-04 |
| **R fusiform gyrus** | 0.29 | 0.08 | 0.14, 0.44 | 1.27 | 3.81 | 1.52E-04 | 2.31E-04 |
| **L inferior temporal gyrus** | 0.28 | 0.08 | 0.13, 0.43 | 1.41 | 3.62 | 3.18E-04 | 4.74E-04 |
| **L superior parietal cortex** | 0.27 | 0.08 | 0.12, 0.42 | 1.38 | 3.50 | 4.98E-04 | 7.27E-04 |
| **L inferior parietal cortex** | 0.25 | 0.08 | 0.1, 0.4 | 1.15 | 3.30 | 1.01E-03 | 1.44E-03 |
| **L lateral occipital cortex** | 0.22 | 0.08 | 0.07, 0.37 | 1.09 | 2.86 | 4.40E-03 | 6.16E-03 |
| **R inferior parietal cortex** | 0.22 | 0.08 | 0.07, 0.37 | 0.96 | 2.84 | 4.69E-03 | 6.44E-03 |
| **L rostral anterior cingulate** | 0.22 | 0.08 | 0.07, 0.36 | 1.60 | 2.81 | 5.14E-03 | 6.79E-03 |
| **L fusiform gyrus** | 0.19 | 0.08 | 0.04, 0.34 | 0.84 | 2.53 | 1.17E-02 | 1.49E-02 |
| **R lateral occipital cortex** | 0.18 | 0.08 | 0.03, 0.33 | 0.91 | 2.39 | 1.72E-02 | 2.15E-02 |
| **R rostral anterior cingulate** | 0.16 | 0.08 | 0.01, 0.31 | 1.13 | 2.05 | 4.09E-02 | 4.95E-02 |
| **L entorhinal cortex** | 0.16 | 0.08 | 0.01, 0.31 | 1.75 | 2.05 | 4.10E-02 | 4.95E-02 |
| **R frontal pole** | 0.14 | 0.08 | -0.01, 0.29 | 1.35 | 1.87 | 6.12E-02 | 7.27E-02 |
| **L frontal pole** | 0.10 | 0.08 | -0.05, 0.25 | 1.02 | 1.35 | 1.78E-01 | 2.01E-01 |
| **R transverse temporal gyrus** | 0.06 | 0.08 | -0.09, 0.21 | 0.48 | 0.78 | 4.36E-01 | 4.70E-01 |
| **R temporal pole** | 0.01 | 0.08 | -0.14, 0.16 | 0.10 | 0.12 | 9.01E-01 | 9.28E-01 |
| **R entorhinal cortex** | -0.01 | 0.08 | -0.15, 0.14 | -0.07 | -0.07 | 9.42E-01 | 9.42E-01 |
| **R banks of STS** | -0.01 | 0.08 | -0.16, 0.14 | -0.04 | -0.09 | 9.32E-01 | 9.42E-01 |
| **R caudal anterior cingulate** | -0.03 | 0.08 | -0.18, 0.12 | -0.19 | -0.35 | 7.28E-01 | 7.61E-01 |
| **L banks of STS** | -0.03 | 0.08 | -0.18, 0.12 | -0.19 | -0.43 | 6.70E-01 | 7.11E-01 |
| **L temporal pole** | -0.07 | 0.08 | -0.22, 0.08 | -0.73 | -0.87 | 3.83E-01 | 4.19E-01 |
| **R isthmus cingulate** | -0.09 | 0.08 | -0.24, 0.06 | -0.49 | -1.15 | 2.52E-01 | 2.81E-01 |
| **L posterior cingulate** | -0.13 | 0.08 | -0.28, 0.02 | -0.62 | -1.69 | 9.17E-02 | 1.05E-01 |
| **R posterior cingulate** | -0.13 | 0.08 | -0.28, 0.02 | -0.60 | -1.73 | 8.36E-02 | 9.75E-02 |
| **L caudal anterior cingulate** | -0.21 | 0.08 | -0.36, -0.06 | -1.44 | -2.77 | 5.68E-03 | 7.36E-03 |
| **L transverse temporal gyrus** | -0.22 | 0.08 | -0.37, -0.07 | -1.62 | -2.81 | 5.02E-03 | 6.76E-03 |
| **R parahippocampal gyrus** | -0.30 | 0.08 | -0.45, -0.15 | -2.75 | -3.85 | 1.30E-04 | 2.02E-04 |
| **R STG** | -0.32 | 0.08 | -0.47, -0.17 | -1.57 | -4.14 | 3.88E-05 | 6.47E-05 |
| **L STG** | -0.50 | 0.08 | -0.65, -0.35 | -2.49 | -6.48 | 1.77E-10 | 4.95E-10 |
| **L parahippocampal gyrus** | -0.59 | 0.08 | -0.74, -0.44 | -5.65 | -7.65 | 6.80E-14 | 3.40E-13 |

**Table S10a:** Group Differences in Cortical Thickness, 22q11DS Cases vs Controls: Effect size (Cohen’s *d*), 95% Confidence Interval (CI), Percent Difference, and P-Value. The following linear model was used for the group comparison: CT ~ Group + Site + Sex + Age + Age^2^. Degrees of freedom (df) =686 for all comparisons.

# **Table S10b: Group Differences in SA, 22q11DS Cases vs Controls.**

| **Region** | **Cohen d** | **Std. Err.** | **95% CI** | **% Diff** | **T-value** | **P-value** | **P-FDR** |
| --- | --- | --- | --- | --- | --- | --- | --- |
| **L cuneus** | -1.53 | 0.09 | -1.7, -1.36 | -39.57 | -19.96 | 3.18E-70 | 2.23E-68 |
| **R cuneus** | -1.47 | 0.09 | -1.63, -1.3 | -39.50 | -19.11 | 1.37E-65 | 4.78E-64 |
| **R lingual gyrus** | -1.43 | 0.09 | -1.6, -1.26 | -31.45 | -18.62 | 6.03E-63 | 1.41E-61 |
| **L lingual gyrus** | -1.36 | 0.08 | -1.53, -1.2 | -36.19 | -17.76 | 2.50E-58 | 4.38E-57 |
| **L precuneus** | -1.32 | 0.08 | -1.48, -1.16 | -33.06 | -17.19 | 2.39E-55 | 3.34E-54 |
| **R precuneus** | -1.28 | 0.08 | -1.45, -1.12 | -38.62 | -16.72 | 6.65E-53 | 7.76E-52 |
| **R pericalcarine cortex** | -1.22 | 0.08 | -1.38, -1.06 | -36.58 | -15.88 | 1.30E-48 | 1.30E-47 |
| **L pericalcarine cortex** | -1.18 | 0.08 | -1.34, -1.02 | -35.74 | -15.37 | 4.88E-46 | 4.27E-45 |
| **L caudal anterior cingulate** | -1.10 | 0.08 | -1.26, -0.94 | -48.34 | -14.37 | 4.04E-41 | 3.14E-40 |
| **L superior parietal cortex** | -1.10 | 0.08 | -1.26, -0.94 | -26.25 | -14.30 | 9.02E-41 | 6.31E-40 |
| **R superior parietal cortex** | -1.06 | 0.08 | -1.22, -0.91 | -21.90 | -13.87 | 9.75E-39 | 6.21E-38 |
| **R fusiform gyrus** | -1.05 | 0.08 | -1.21, -0.89 | -35.37 | -13.68 | 7.55E-38 | 4.40E-37 |
| **R hemisphere** | -1.02 | 0.08 | -1.18, -0.87 | -19.15 | -13.34 | 3.05E-36 | 1.64E-35 |
| **L hemisphere** | -1.02 | 0.08 | -1.18, -0.86 | -19.33 | -13.26 | 7.21E-36 | 3.61E-35 |
| **L fusiform gyrus** | -1.01 | 0.08 | -1.17, -0.86 | -31.72 | -13.22 | 1.07E-35 | 4.98E-35 |
| **R rostral middle frontal gyrus** | -0.94 | 0.08 | -1.09, -0.78 | -29.14 | -12.22 | 3.44E-31 | 1.50E-30 |
| **L rostral middle frontal gyrus** | -0.86 | 0.08 | -1.01, -0.7 | -30.33 | -11.18 | 8.94E-27 | 3.68E-26 |
| **R caudal anterior cingulate** | -0.81 | 0.08 | -0.97, -0.66 | -43.71 | -10.61 | 1.93E-24 | 7.52E-24 |
| **L lateral occipital cortex** | -0.81 | 0.08 | -0.97, -0.66 | -23.70 | -10.57 | 2.58E-24 | 9.50E-24 |
| **R lateral occipital cortex** | -0.80 | 0.08 | -0.95, -0.65 | -24.78 | -10.42 | 1.06E-23 | 3.70E-23 |
| **R postcentral gyrus** | -0.77 | 0.08 | -0.93, -0.62 | -21.08 | -10.07 | 2.50E-22 | 8.32E-22 |
| **R inferior temporal gyrus** | -0.75 | 0.08 | -0.9, -0.6 | -64.87 | -9.77 | 3.51E-21 | 1.12E-20 |
| **L inferior temporal gyrus** | -0.70 | 0.08 | -0.86, -0.55 | -61.46 | -9.14 | 6.81E-19 | 2.07E-18 |
| **L rostral anterior cingulate** | -0.69 | 0.08 | -0.84, -0.54 | 105.83 | -9.01 | 2.04E-18 | 5.96E-18 |
| **L postcentral gyrus** | -0.68 | 0.08 | -0.84, -0.53 | -17.44 | -8.89 | 5.27E-18 | 1.47E-17 |
| **L temporal pole** | -0.61 | 0.08 | -0.76, -0.46 | -16.18 | -7.92 | 9.38E-15 | 2.53E-14 |
| **R pars triangularis** | -0.57 | 0.08 | -0.72, -0.41 | -21.50 | -7.36 | 5.16E-13 | 1.34E-12 |
| **R temporal pole** | -0.55 | 0.08 | -0.7, -0.4 | -14.79 | -7.18 | 1.83E-12 | 4.59E-12 |
| **L pars triangularis** | -0.53 | 0.08 | -0.68, -0.38 | -15.61 | -6.95 | 8.78E-12 | 2.12E-11 |
| **R paracentral lobule** | -0.50 | 0.08 | -0.66, -0.35 | -21.41 | -6.57 | 1.00E-10 | 2.34E-10 |
| **L middle temporal gyrus** | -0.50 | 0.08 | -0.65, -0.35 | -24.92 | -6.47 | 1.89E-10 | 4.14E-10 |
| **R entorhinal cortex** | -0.49 | 0.08 | -0.64, -0.34 | -26.31 | -6.41 | 2.71E-10 | 5.75E-10 |
| **R rostral anterior cingulate** | -0.49 | 0.08 | -0.64, -0.33 | -1191.05 | -6.33 | 4.53E-10 | 9.32E-10 |
| **R supramarginal gyrus** | -0.47 | 0.08 | -0.62, -0.32 | -17.69 | -6.10 | 1.74E-09 | 3.49E-09 |
| **L posterior cingulate** | -0.42 | 0.08 | -0.57, -0.27 | -13.99 | -5.45 | 7.19E-08 | 1.40E-07 |
| **R middle temporal gyrus** | -0.40 | 0.08 | -0.55, -0.25 | -19.98 | -5.15 | 3.35E-07 | 6.16E-07 |
| **L STG** | -0.39 | 0.08 | -0.55, -0.24 | -11.21 | -5.14 | 3.51E-07 | 6.31E-07 |
| **L entorhinal cortex** | -0.39 | 0.08 | -0.54, -0.24 | -34.50 | -5.13 | 3.70E-07 | 6.47E-07 |
| **L transverse temporal gyrus** | -0.39 | 0.08 | -0.54, -0.24 | -16.05 | -5.03 | 6.24E-07 | 1.06E-06 |
| **R STG** | -0.37 | 0.08 | -0.52, -0.22 | -8.52 | -4.84 | 1.60E-06 | 2.67E-06 |
| **R transverse temporal gyrus** | -0.37 | 0.08 | -0.52, -0.22 | -17.28 | -4.81 | 1.86E-06 | 3.03E-06 |
| **L banks of STS** | -0.35 | 0.08 | -0.5, -0.2 | -20.80 | -4.51 | 7.74E-06 | 1.23E-05 |
| **L superior frontal gyrus** | -0.34 | 0.08 | -0.49, -0.19 | -10.74 | -4.49 | 8.33E-06 | 1.30E-05 |
| **R posterior cingulate** | -0.33 | 0.08 | -0.48, -0.18 | -21.40 | -4.29 | 2.06E-05 | 3.13E-05 |
| **R medial orbitofrontal cortex** | -0.31 | 0.08 | -0.46, -0.16 | -17.40 | -4.08 | 4.94E-05 | 7.20E-05 |
| **L paracentral lobule** | -0.29 | 0.08 | -0.44, -0.14 | -13.36 | -3.75 | 1.91E-04 | 2.67E-04 |
| **L pars orbitalis** | -0.26 | 0.08 | -0.41, -0.11 | -9.06 | -3.37 | 8.05E-04 | 1.11E-03 |
| **R banks of STS** | -0.24 | 0.08 | -0.39, -0.09 | -11.05 | -3.15 | 1.70E-03 | 2.29E-03 |
| **R parahippocampal gyrus** | -0.21 | 0.08 | -0.36, -0.07 | -7.80 | -2.80 | 5.24E-03 | 6.92E-03 |
| **R pars orbitalis** | -0.21 | 0.08 | -0.36, -0.06 | -5.68 | -2.73 | 6.43E-03 | 8.34E-03 |
| **L lateral orbitofrontal cortex** | -0.21 | 0.08 | -0.35, -0.06 | -12.17 | -2.67 | 7.70E-03 | 9.79E-03 |
| **L pars opercularis** | -0.19 | 0.08 | -0.34, -0.04 | -7.96 | -2.50 | 1.28E-02 | 1.60E-02 |
| **L frontal pole** | -0.17 | 0.08 | -0.32, -0.03 | -4.64 | -2.28 | 2.30E-02 | 2.83E-02 |
| **L medial orbitofrontal cortex** | -0.16 | 0.08 | -0.3, -0.01 | -11.75 | -2.02 | 4.37E-02 | 5.28E-02 |
| **R inferior parietal cortex** | -0.15 | 0.08 | -0.3, 0 | -7.13 | -2.00 | 4.60E-02 | 5.45E-02 |
| **L supramarginal gyrus** | -0.15 | 0.08 | -0.3, 0 | -7.46 | -1.98 | 4.85E-02 | 5.66E-02 |
| **R lateral orbitofrontal cortex** | -0.14 | 0.08 | -0.29, 0.01 | -7.15 | -1.83 | 6.75E-02 | 7.74E-02 |
| **L isthmus cingulate** | -0.13 | 0.08 | -0.28, 0.02 | -12.99 | -1.72 | 8.50E-02 | 9.60E-02 |
| **R superior frontal gyrus** | -0.13 | 0.08 | -0.28, 0.02 | -4.87 | -1.65 | 1.00E-01 | 1.11E-01 |
| **L inferior parietal cortex** | -0.10 | 0.08 | -0.25, 0.05 | -4.14 | -1.30 | 1.94E-01 | 2.12E-01 |
| **R caudal middle frontal gyrus** | -0.07 | 0.08 | -0.21, 0.08 | -4.84 | -0.85 | 3.93E-01 | 4.17E-01 |
| **L caudal middle frontal gyrus** | -0.04 | 0.08 | -0.19, 0.11 | -3.51 | -0.49 | 6.22E-01 | 6.50E-01 |
| **R pars opercularis** | -0.01 | 0.08 | -0.16, 0.14 | -0.48 | -0.12 | 9.05E-01 | 9.05E-01 |
| **R isthmus cingulate** | 0.03 | 0.08 | -0.12, 0.17 | 3.27 | 0.34 | 7.34E-01 | 7.45E-01 |
| **R frontal pole** | 0.03 | 0.08 | -0.12, 0.18 | 1.07 | 0.44 | 6.62E-01 | 6.82E-01 |
| **L parahippocampal gyrus** | 0.07 | 0.08 | -0.08, 0.22 | 2.25 | 0.94 | 3.47E-01 | 3.74E-01 |
| **L insula** | 0.31 | 0.08 | 0.16, 0.46 | 18.57 | 4.00 | 7.10E-05 | 1.01E-04 |
| **R precentral gyrus** | 0.32 | 0.08 | 0.17, 0.47 | 8.62 | 4.20 | 3.07E-05 | 4.57E-05 |
| **L precentral gyrus** | 0.41 | 0.08 | 0.26, 0.56 | 11.64 | 5.30 | 1.58E-07 | 2.99E-07 |
| **R insula** | 0.50 | 0.08 | 0.35, 0.65 | 47.41 | 6.47 | 1.89E-10 | 4.14E-10 |

**Table S10b:** Group Differences in Surface Area, 22q11DS Cases vs Controls: Effect size (Cohen’s d), 95% Confidence Interval (CI), Percent Difference, and P-Value. The following linear model was used for the group comparison: SA ~ Group + Site + Sex + Age + ICV. Df= 686 for all comparisons.

# **Table S11a: Group Differences in CT, 22q11DS Cases vs Controls, Mixed Effect Models.**

| **Region** | **Cohen d** | **Std. Err.** | **95% CI** | **%Diff** | **T-value** | **P-value** | **P-FDR** |
| --- | --- | --- | --- | --- | --- | --- | --- |
| **L insula** | 0.87 | 0.08 | 0.72, 1.03 | 3.88 | 11.36 | 1.65E-27 | 1.15E-25 |
| **R rostral middle frontal gyrus** | 0.79 | 0.08 | 0.64, 0.95 | 4.11 | 10.35 | 1.97E-23 | 6.90E-22 |
| **L pericalcarine cortex** | 0.73 | 0.08 | 0.57, 0.88 | 5.88 | 9.49 | 3.72E-20 | 8.69E-19 |
| **R supramarginal gyrus** | 0.71 | 0.08 | 0.56, 0.86 | 3.35 | 9.23 | 3.31E-19 | 5.79E-18 |
| **L rostral middle frontal gyrus** | 0.69 | 0.08 | 0.54, 0.84 | 3.47 | 8.99 | 2.40E-18 | 3.36E-17 |
| **R pericalcarine cortex** | 0.68 | 0.08 | 0.53, 0.84 | 5.43 | 8.91 | 4.42E-18 | 5.16E-17 |
| **R hemisphere** | 0.64 | 0.08 | 0.49, 0.8 | 2.34 | 8.40 | 2.66E-16 | 2.66E-15 |
| **R insula** | 0.63 | 0.08 | 0.48, 0.78 | 3.10 | 8.24 | 9.00E-16 | 7.88E-15 |
| **R caudal middle frontal gyrus** | 0.62 | 0.08 | 0.47, 0.77 | 3.36 | 8.10 | 2.55E-15 | 1.98E-14 |
| **L postcentral gyrus** | 0.62 | 0.08 | 0.47, 0.77 | 3.39 | 8.08 | 2.88E-15 | 2.01E-14 |
| **L hemisphere** | 0.61 | 0.08 | 0.46, 0.76 | 2.18 | 7.97 | 6.55E-15 | 4.17E-14 |
| **R medial orbitofrontal cortex** | 0.60 | 0.08 | 0.45, 0.76 | 3.72 | 7.86 | 1.48E-14 | 8.66E-14 |
| **L supramarginal gyrus** | 0.59 | 0.08 | 0.44, 0.75 | 2.93 | 7.74 | 3.51E-14 | 1.89E-13 |
| **R cuneus** | 0.58 | 0.08 | 0.43, 0.74 | 3.65 | 7.61 | 8.91E-14 | 4.45E-13 |
| **R paracentral lobule** | 0.57 | 0.08 | 0.41, 0.72 | 3.04 | 7.37 | 5.05E-13 | 2.17E-12 |
| **L pars opercularis** | 0.56 | 0.08 | 0.41, 0.72 | 2.75 | 7.36 | 5.27E-13 | 2.17E-12 |
| **L medial orbitofrontal cortex** | 0.56 | 0.08 | 0.4, 0.71 | 3.41 | 7.25 | 1.12E-12 | 4.34E-12 |
| **R lingual gyrus** | 0.55 | 0.08 | 0.4, 0.7 | 3.07 | 7.17 | 1.93E-12 | 7.09E-12 |
| **L paracentral lobule** | 0.55 | 0.08 | 0.39, 0.7 | 3.09 | 7.12 | 2.78E-12 | 9.73E-12 |
| **L cuneus** | 0.53 | 0.08 | 0.38, 0.68 | 3.51 | 6.87 | 1.42E-11 | 4.72E-11 |
| **R superior frontal gyrus** | 0.52 | 0.08 | 0.37, 0.67 | 2.32 | 6.81 | 2.18E-11 | 6.92E-11 |
| **L caudal middle frontal gyrus** | 0.51 | 0.08 | 0.36, 0.66 | 2.49 | 6.60 | 8.45E-11 | 2.57E-10 |
| **R precuneus** | 0.50 | 0.08 | 0.34, 0.65 | 2.29 | 6.46 | 2.02E-10 | 5.89E-10 |
| **L pars triangularis** | 0.49 | 0.08 | 0.34, 0.64 | 2.83 | 6.42 | 2.58E-10 | 7.23E-10 |
| **L superior frontal gyrus** | 0.49 | 0.08 | 0.34, 0.64 | 2.17 | 6.34 | 4.11E-10 | 1.06E-09 |
| **L lateral orbitofrontal cortex** | 0.47 | 0.08 | 0.32, 0.62 | 2.48 | 6.07 | 2.10E-09 | 5.26E-09 |
| **R postcentral gyrus** | 0.44 | 0.08 | 0.29, 0.59 | 2.60 | 5.77 | 1.21E-08 | 2.92E-08 |
| **R pars triangularis** | 0.44 | 0.08 | 0.29, 0.59 | 2.53 | 5.70 | 1.76E-08 | 4.11E-08 |
| **L pars orbitalis** | 0.43 | 0.08 | 0.28, 0.58 | 3.04 | 5.61 | 2.87E-08 | 6.49E-08 |
| **L precentral gyrus** | 0.42 | 0.08 | 0.27, 0.57 | 2.14 | 5.53 | 4.61E-08 | 1.01E-07 |
| **R middle temporal gyrus** | 0.42 | 0.08 | 0.27, 0.57 | 2.10 | 5.52 | 4.77E-08 | 1.01E-07 |
| **R lateral orbitofrontal cortex** | 0.42 | 0.08 | 0.27, 0.57 | 2.36 | 5.42 | 8.07E-08 | 1.66E-07 |
| **L lingual gyrus** | 0.41 | 0.08 | 0.26, 0.57 | 2.24 | 5.41 | 8.87E-08 | 1.77E-07 |
| **R precentral gyrus** | 0.41 | 0.08 | 0.26, 0.56 | 2.19 | 5.37 | 1.06E-07 | 2.07E-07 |
| **L precuneus** | 0.37 | 0.08 | 0.22, 0.52 | 1.68 | 4.86 | 1.43E-06 | 2.71E-06 |
| **L middle temporal gyrus** | 0.37 | 0.08 | 0.22, 0.52 | 1.89 | 4.84 | 1.62E-06 | 2.98E-06 |
| **R pars orbitalis** | 0.36 | 0.08 | 0.21, 0.51 | 2.52 | 4.70 | 3.20E-06 | 5.74E-06 |
| **R inferior temporal gyrus** | 0.34 | 0.08 | 0.19, 0.49 | 1.78 | 4.48 | 8.93E-06 | 1.56E-05 |
| **R pars opercularis** | 0.33 | 0.08 | 0.18, 0.48 | 1.79 | 4.33 | 1.72E-05 | 2.94E-05 |
| **R superior parietal cortex** | 0.31 | 0.08 | 0.16, 0.46 | 1.60 | 4.02 | 6.35E-05 | 1.03E-04 |
| **L isthmus cingulate** | 0.30 | 0.08 | 0.15, 0.45 | 1.93 | 3.87 | 1.19E-04 | 1.89E-04 |
| **R fusiform gyrus** | 0.30 | 0.08 | 0.15, 0.45 | 1.35 | 3.86 | 1.24E-04 | 1.92E-04 |
| **L superior parietal cortex** | 0.28 | 0.08 | 0.13, 0.43 | 1.43 | 3.69 | 2.45E-04 | 3.65E-04 |
| **L inferior temporal gyrus** | 0.28 | 0.08 | 0.13, 0.43 | 1.50 | 3.63 | 3.09E-04 | 4.51E-04 |
| **L inferior parietal cortex** | 0.25 | 0.08 | 0.1, 0.4 | 1.19 | 3.27 | 1.11E-03 | 1.59E-03 |
| **L lateral occipital cortex** | 0.22 | 0.08 | 0.07, 0.37 | 1.17 | 2.89 | 4.02E-03 | 5.62E-03 |
| **R inferior parietal cortex** | 0.22 | 0.08 | 0.07, 0.37 | 1.00 | 2.87 | 4.18E-03 | 5.73E-03 |
| **L rostral anterior cingulate** | 0.21 | 0.08 | 0.06, 0.36 | 1.63 | 2.77 | 5.74E-03 | 7.58E-03 |
| **L fusiform gyrus** | 0.20 | 0.08 | 0.05, 0.35 | 0.90 | 2.57 | 1.03E-02 | 1.32E-02 |
| **R lateral occipital cortex** | 0.19 | 0.08 | 0.04, 0.34 | 1.00 | 2.43 | 1.55E-02 | 1.94E-02 |
| **L entorhinal cortex** | 0.17 | 0.08 | 0.02, 0.32 | 1.91 | 2.20 | 2.83E-02 | 3.48E-02 |
| **R rostral anterior cingulate** | 0.15 | 0.08 | 0, 0.3 | 1.13 | 1.97 | 4.88E-02 | 5.89E-02 |
| **R frontal pole** | 0.14 | 0.08 | -0.01, 0.29 | 1.41 | 1.82 | 6.86E-02 | 8.13E-02 |
| **L frontal pole** | 0.10 | 0.08 | -0.05, 0.25 | 1.00 | 1.27 | 2.04E-01 | 2.31E-01 |
| **R transverse temporal gyrus** | 0.07 | 0.08 | -0.08, 0.22 | 0.57 | 0.89 | 3.76E-01 | 4.11E-01 |
| **R temporal pole** | 0.02 | 0.08 | -0.13, 0.17 | 0.21 | 0.26 | 7.92E-01 | 8.15E-01 |
| **R entorhinal cortex** | 0.00 | 0.08 | -0.15, 0.15 | 0.03 | 0.03 | 9.74E-01 | 9.88E-01 |
| **R banks of STS** | 0.00 | 0.08 | -0.15, 0.15 | 0.01 | 0.01 | 9.91E-01 | 9.91E-01 |
| **R caudal anterior cingulate** | -0.03 | 0.08 | -0.18, 0.12 | -0.23 | -0.38 | 7.06E-01 | 7.38E-01 |
| **L banks of STS** | -0.03 | 0.08 | -0.18, 0.12 | -0.20 | -0.42 | 6.74E-01 | 7.15E-01 |
| **L temporal pole** | -0.06 | 0.08 | -0.21, 0.09 | -0.62 | -0.76 | 4.50E-01 | 4.85E-01 |
| **R isthmus cingulate** | -0.09 | 0.08 | -0.24, 0.06 | -0.55 | -1.15 | 2.52E-01 | 2.80E-01 |
| **L posterior cingulate** | -0.13 | 0.08 | -0.28, 0.02 | -0.68 | -1.70 | 8.95E-02 | 1.03E-01 |
| **R posterior cingulate** | -0.13 | 0.08 | -0.28, 0.02 | -0.69 | -1.74 | 8.24E-02 | 9.61E-02 |
| **L transverse temporal gyrus** | -0.21 | 0.08 | -0.36, -0.06 | -1.65 | -2.73 | 6.44E-03 | 8.35E-03 |
| **L caudal anterior cingulate** | -0.21 | 0.08 | -0.36, -0.06 | -1.65 | -2.78 | 5.52E-03 | 7.43E-03 |
| **R parahippocampal gyrus** | -0.29 | 0.08 | -0.44, -0.14 | -2.64 | -3.74 | 2.01E-04 | 3.06E-04 |
| **R STG** | -0.32 | 0.08 | -0.47, -0.17 | -1.59 | -4.18 | 3.31E-05 | 5.51E-05 |
| **L STG** | -0.49 | 0.08 | -0.64, -0.34 | -2.49 | -6.36 | 3.59E-10 | 9.67E-10 |
| **L parahippocampal gyrus** | -0.58 | 0.08 | -0.73, -0.42 | -5.53 | -7.51 | 1.88E-13 | 8.79E-13 |

**Table S11a:** Group Differences in Cortical Thickness, 22q11DS Cases vs Controls, Mixed Effect Models: Effect size (Cohen’s d), 95% Confidence Interval (CI), Percent Difference, and P-Value. The following mixed effect linear model was used for the group comparison: CT ~ Group + Sex + Age + Age^2^, random = ~ 1 | Site. Df=686 for all comparisons.

# **Table S11b: Group Differences in SA, 22q11DS Cases vs Controls, Mixed Effect Models.**

| **Region** | **Cohen d** | **Std. Err.** | **95% CI** | **% Diff** | **T-value** | **P-value** | **P-FDR** |
| --- | --- | --- | --- | --- | --- | --- | --- |
| **L cuneus** | -1.54 | 0.086 | -1.71, -1.37 | -34.12 | -20.09 | 5.98E-71 | 4.18E-69 |
| **R cuneus** | -1.48 | 0.086 | -1.65, -1.31 | -33.67 | -19.30 | 1.20E-66 | 4.20E-65 |
| **R lingual gyrus** | -1.44 | 0.085 | -1.61, -1.28 | -28.76 | -18.80 | 6.22E-64 | 1.45E-62 |
| **L lingual gyrus** | -1.38 | 0.084 | -1.54, -1.21 | -31.11 | -17.96 | 2.15E-59 | 3.77E-58 |
| **L precuneus** | -1.33 | 0.084 | -1.49, -1.16 | -26.86 | -17.30 | 6.62E-56 | 9.26E-55 |
| **R precuneus** | -1.29 | 0.083 | -1.46, -1.13 | -30.09 | -16.85 | 1.39E-53 | 1.62E-52 |
| **R pericalcarine cortex** | -1.23 | 0.083 | -1.39, -1.06 | -29.65 | -15.96 | 5.12E-49 | 5.12E-48 |
| **L pericalcarine cortex** | -1.19 | 0.082 | -1.35, -1.03 | -30.56 | -15.46 | 1.85E-46 | 1.62E-45 |
| **L caudal anterior cingulate** | -1.12 | 0.082 | -1.28, -0.96 | -37.72 | -14.62 | 2.50E-42 | 1.94E-41 |
| **L superior parietal cortex** | -1.12 | 0.082 | -1.28, -0.96 | -22.69 | -14.53 | 6.70E-42 | 4.69E-41 |
| **R superior parietal cortex** | -1.08 | 0.081 | -1.24, -0.92 | -19.49 | -14.02 | 1.87E-39 | 1.19E-38 |
| **R fusiform gyrus** | -1.05 | 0.081 | -1.21, -0.9 | -24.52 | -13.74 | 3.99E-38 | 2.33E-37 |
| **R hemisphere** | -1.03 | 0.081 | -1.19, -0.87 | -14.93 | -13.40 | 1.47E-36 | 7.93E-36 |
| **L hemisphere** | -1.02 | 0.081 | -1.18, -0.86 | -15.12 | -13.32 | 3.45E-36 | 1.73E-35 |
| **L fusiform gyrus** | -1.02 | 0.081 | -1.18, -0.86 | -24.74 | -13.28 | 5.37E-36 | 2.51E-35 |
| **R rostral middle frontal gyrus** | -0.95 | 0.080 | -1.1, -0.79 | -23.29 | -12.33 | 1.08E-31 | 4.71E-31 |
| **L rostral middle frontal gyrus** | -0.86 | 0.079 | -1.02, -0.71 | -23.42 | -11.24 | 5.28E-27 | 2.18E-26 |
| **L lateral occipital cortex** | -0.83 | 0.079 | -0.99, -0.68 | -19.39 | -10.87 | 1.71E-25 | 6.66E-25 |
| **R lateral occipital cortex** | -0.82 | 0.079 | -0.97, -0.66 | -19.72 | -10.67 | 1.11E-24 | 4.09E-24 |
| **R caudal anterior cingulate** | -0.81 | 0.079 | -0.97, -0.66 | -35.01 | -10.60 | 2.03E-24 | 7.10E-24 |
| **R postcentral gyrus** | -0.78 | 0.079 | -0.94, -0.63 | -17.60 | -10.21 | 6.98E-23 | 2.33E-22 |
| **R inferior temporal gyrus** | -0.75 | 0.079 | -0.91, -0.6 | -29.49 | -9.80 | 2.50E-21 | 7.96E-21 |
| **L inferior temporal gyrus** | -0.71 | 0.078 | -0.86, -0.55 | -31.70 | -9.20 | 4.20E-19 | 1.28E-18 |
| **L rostral anterior cingulate** | -0.70 | 0.078 | -0.85, -0.55 | -160.36 | -9.14 | 7.24E-19 | 2.11E-18 |
| **L postcentral gyrus** | -0.69 | 0.078 | -0.84, -0.54 | -15.04 | -9.00 | 2.12E-18 | 5.93E-18 |
| **L temporal pole** | -0.62 | 0.078 | -0.77, -0.47 | -12.78 | -8.08 | 2.91E-15 | 7.84E-15 |
| **R pars triangularis** | -0.58 | 0.078 | -0.73, -0.43 | -18.09 | -7.56 | 1.27E-13 | 3.28E-13 |
| **L pars triangularis** | -0.58 | 0.077 | -0.73, -0.43 | -14.43 | -7.55 | 1.42E-13 | 3.56E-13 |
| **R temporal pole** | -0.56 | 0.077 | -0.72, -0.41 | -12.45 | -7.34 | 6.09E-13 | 1.47E-12 |
| **R paracentral lobule** | -0.52 | 0.077 | -0.68, -0.37 | -16.87 | -6.84 | 1.76E-11 | 4.10E-11 |
| **R entorhinal cortex** | -0.50 | 0.077 | -0.65, -0.35 | -23.82 | -6.55 | 1.15E-10 | 2.59E-10 |
| **L middle temporal gyrus** | -0.50 | 0.077 | -0.65, -0.35 | -16.64 | -6.50 | 1.53E-10 | 3.35E-10 |
| **R rostral anterior cingulate** | -0.49 | 0.077 | -0.64, -0.33 | -43.75 | -6.32 | 4.60E-10 | 9.48E-10 |
| **R supramarginal gyrus** | -0.48 | 0.077 | -0.63, -0.33 | -14.07 | -6.22 | 8.62E-10 | 1.72E-09 |
| **L posterior cingulate** | -0.42 | 0.077 | -0.57, -0.27 | -11.53 | -5.49 | 5.68E-08 | 1.10E-07 |
| **L entorhinal cortex** | -0.41 | 0.077 | -0.56, -0.26 | -21.11 | -5.32 | 1.40E-07 | 2.57E-07 |
| **L transverse temporal gyrus** | -0.40 | 0.077 | -0.55, -0.25 | -13.26 | -5.23 | 2.27E-07 | 4.07E-07 |
| **L STG** | -0.40 | 0.077 | -0.55, -0.25 | -8.83 | -5.19 | 2.72E-07 | 4.75E-07 |
| **R middle temporal gyrus** | -0.40 | 0.077 | -0.55, -0.25 | -12.12 | -5.18 | 2.86E-07 | 4.88E-07 |
| **R transverse temporal gyrus** | -0.39 | 0.077 | -0.54, -0.24 | -14.36 | -5.09 | 4.66E-07 | 7.77E-07 |
| **R STG** | -0.38 | 0.077 | -0.53, -0.23 | -7.24 | -4.92 | 1.08E-06 | 1.76E-06 |
| **L superior frontal gyrus** | -0.35 | 0.077 | -0.5, -0.2 | -8.09 | -4.57 | 5.80E-06 | 9.23E-06 |
| **L banks of STS** | -0.35 | 0.077 | -0.5, -0.2 | -13.77 | -4.55 | 6.29E-06 | 9.78E-06 |
| **R posterior cingulate** | -0.34 | 0.076 | -0.49, -0.19 | -13.28 | -4.39 | 1.31E-05 | 1.99E-05 |
| **R medial orbitofrontal cortex** | -0.32 | 0.076 | -0.47, -0.17 | -8.16 | -4.15 | 3.70E-05 | 5.39E-05 |
| **L paracentral lobule** | -0.30 | 0.076 | -0.45, -0.15 | -10.33 | -3.92 | 9.60E-05 | 1.34E-04 |
| **L pars orbitalis** | -0.26 | 0.076 | -0.41, -0.11 | -7.09 | -3.44 | 6.27E-04 | 8.61E-04 |
| **R banks of STS** | -0.25 | 0.076 | -0.4, -0.1 | -7.61 | -3.22 | 1.32E-03 | 1.78E-03 |
| **R parahippocampal gyrus** | -0.22 | 0.076 | -0.37, -0.07 | -7.08 | -2.87 | 4.24E-03 | 5.60E-03 |
| **L pars opercularis** | -0.22 | 0.076 | -0.37, -0.07 | -7.07 | -2.85 | 4.49E-03 | 5.83E-03 |
| **R pars orbitalis** | -0.21 | 0.076 | -0.36, -0.06 | -4.98 | -2.77 | 5.83E-03 | 7.42E-03 |
| **L lateral orbitofrontal cortex** | -0.21 | 0.076 | -0.36, -0.06 | -5.86 | -2.70 | 7.04E-03 | 8.79E-03 |
| **L frontal pole** | -0.18 | 0.076 | -0.33, -0.04 | -4.44 | -2.40 | 1.67E-02 | 2.05E-02 |
| **L medial orbitofrontal cortex** | -0.16 | 0.076 | -0.31, -0.01 | -6.02 | -2.13 | 3.37E-02 | 4.06E-02 |
| **R inferior parietal cortex** | -0.16 | 0.076 | -0.31, -0.01 | -5.17 | -2.10 | 3.63E-02 | 4.31E-02 |
| **L supramarginal gyrus** | -0.16 | 0.076 | -0.31, -0.01 | -5.45 | -2.08 | 3.75E-02 | 4.38E-02 |
| **R lateral orbitofrontal cortex** | -0.14 | 0.076 | -0.29, 0 | -4.24 | -1.88 | 6.01E-02 | 6.89E-02 |
| **L isthmus cingulate** | -0.14 | 0.076 | -0.29, 0.01 | -7.33 | -1.83 | 6.82E-02 | 7.70E-02 |
| **R superior frontal gyrus** | -0.13 | 0.076 | -0.28, 0.01 | -3.91 | -1.75 | 7.98E-02 | 8.86E-02 |
| **L inferior parietal cortex** | -0.11 | 0.076 | -0.26, 0.04 | -3.32 | -1.43 | 1.54E-01 | 1.69E-01 |
| **R caudal middle frontal gyrus** | -0.08 | 0.076 | -0.23, 0.07 | -3.88 | -0.99 | 3.22E-01 | 3.46E-01 |
| **L caudal middle frontal gyrus** | -0.05 | 0.076 | -0.2, 0.09 | -3.43 | -0.70 | 4.82E-01 | 5.03E-01 |
| **R pars opercularis** | -0.02 | 0.076 | -0.17, 0.13 | -0.74 | -0.23 | 8.20E-01 | 8.32E-01 |
| **R frontal pole** | 0.01 | 0.076 | -0.14, 0.15 | 0.17 | 0.08 | 9.36E-01 | 9.36E-01 |
| **R isthmus cingulate** | 0.02 | 0.076 | -0.13, 0.17 | 1.17 | 0.25 | 8.03E-01 | 8.27E-01 |
| **L parahippocampal gyrus** | 0.06 | 0.076 | -0.09, 0.21 | 1.63 | 0.82 | 4.15E-01 | 4.40E-01 |
| **L insula** | 0.30 | 0.076 | 0.15, 0.45 | 11.18 | 3.93 | 9.19E-05 | 1.31E-04 |
| **R precentral gyrus** | 0.32 | 0.076 | 0.17, 0.47 | 7.56 | 4.20 | 2.96E-05 | 4.40E-05 |
| **L precentral gyrus** | 0.41 | 0.077 | 0.26, 0.56 | 10.61 | 5.37 | 1.07E-07 | 2.02E-07 |
| **R insula** | 0.49 | 0.077 | 0.34, 0.64 | 24.37 | 6.39 | 3.00E-10 | 6.37E-10 |

**Table S11b:** Group Differences in Surface Area, 22q11DS Cases vs Controls, Mixed Effect Models: Effect size (Cohen’s d), 95% Confidence Interval (CI), Percent Difference, and P-Value. The following mixed effect linear model was used for the group comparison: SA ~ Group + Sex + Age + ICV, random = ~ 1 | Site. Df = 686 for all comparisons.

# **Table S12a. Classification Accuracy – 22q11DS Cases vs Controls**

**Table S12a:** Classification Analysis: Accuracy, Sensitivity and Specificity. Random divisions were applied to the whole dataset to obtain 20 training sets and 20 corresponding testing sets at a ratio of 3:1 in numbers of subjects. For each division, the glmnet algorithm was used to calculate the sensitivity, specificity, and accuracy, as well as the significance level, of group prediction. These above values were averaged over the 20 divisions.

**Table S12b:** Classification Analysis, GLMNet Weights for Cortical Structures

| Region | Weight |
| --- | --- |
| lh_caudalanteriorcingulate_area | 0.3499 |
| lh_insula_thickness | 0.3148 |
| lh_precentral_area | 0.3033 |
| rh_cuneus_area | 0.2729 |
| lh_cuneus_area | 0.2575 |
| rh_lingual_area | 0.2505 |
| lh_precuneus_area | 0.2418 |
| lh_insula_area | 0.2415 |
| lh_lingual_area | 0.2263 |
| rh_precentral_area | 0.2247 |
| rh_insula_area | 0.2226 |
| rh_precuneus_area | 0.2125 |
| rh_parstriangularis_area | 0.2039 |
| lh_caudalanteriorcingulate_thickness | 0.2020 |
| lh_superiortemporal_thickness | 0.1934 |
| lh_superiorparietal_area | 0.1904 |
| rh_paracentral_area | 0.1839 |
| rh_rostralmiddlefrontal_area | 0.1790 |
| rh_pericalcarine_thickness | 0.1681 |
| rh_superiortemporal_thickness | 0.1667 |
| lh_parahippocampal_thickness | 0.1651 |
| rh_caudalanteriorcingulate_area | 0.1647 |
| rh_insula_thickness | 0.1548 |
| lh_supramarginal_area | 0.1498 |
| rh_temporalpole_area | 0.1470 |
| rh_fusiform_area | 0.1406 |
| lh_transversetemporal_thickness | 0.1370 |
| rh_superiorparietal_area | 0.1359 |
| lh_inferiorparietal_area | 0.1324 |
| lh_pericalcarine_thickness | 0.1316 |
| lh_temporalpole_area | 0.1272 |
| lh_fusiform_area | 0.1192 |
| rh_superiorfrontal_area | 0.1187 |
| rh_isthmuscingulate_area | 0.1152 |
| rh_supramarginal_thickness | 0.1144 |
| rh_caudalmiddlefrontal_thickness | 0.1068 |
| lh_pericalcarine_area | 0.1007 |
| rh_pericalcarine_area | 0.0933 |
| lh_paracentral_thickness | 0.0923 |
| rh_postcentral_area | 0.0909 |
| rh_lateralorbitofrontal_area | 0.0872 |
| rh_isthmuscingulate_thickness | 0.0846 |
| rh_caudalanteriorcingulate_thickness | 0.0844 |
| lh_entorhinal_thickness | 0.0783 |
| lh_parsopercularis_thickness | 0.0740 |
| rh_parahippocampal_thickness | 0.0725 |
| lh_lateraloccipital_area | 0.0687 |
| lh_isthmuscingulate_area | 0.0686 |
| rh_posteriorcingulate_thickness | 0.0667 |
| rh_inferiorparietal_area | 0.0637 |
| rh_lingual_thickness | 0.0617 |
| lh_rostralmiddlefrontal_area | 0.0593 |
| lh_postcentral_thickness | 0.0540 |
| lh_cuneus_thickness | 0.0526 |
| lh_bankssts_thickness | 0.0510 |
| rh_parahippocampal_area | 0.0496 |
| lh_posteriorcingulate_thickness | 0.0476 |
| lh_parstriangularis_thickness | 0.0467 |
| lh_rostralanteriorcingulate_area | 0.0427 |
| lh_paracentral_area | 0.0420 |
| lh_parahippocampal_area | 0.0408 |
| rh_frontalpole_area | 0.0392 |
| rh_transversetemporal_thickness | 0.0388 |
| rh_parsopercularis_area | 0.0386 |
| lh_rostralmiddlefrontal_thickness | 0.0369 |
| rh_paracentral_thickness | 0.0365 |
| lh_supramarginal_thickness | 0.0362 |
| lh_lateraloccipital_thickness | 0.0338 |
| rh_lateraloccipital_thickness | 0.0302 |
| rh_inferiortemporal_area | 0.0293 |
| rh_bankssts_area | 0.0283 |
| rh_parsorbitalis_thickness | 0.0251 |
| rh_entorhinal_area | 0.0215 |
| rh_parsorbitalis_area | 0.0192 |
| lh_superiorfrontal_area | 0.0183 |
| lh_caudalmiddlefrontal_area | 0.0180 |
| rh_lateraloccipital_area | 0.0167 |
| lh_lateralorbitofrontal_area | 0.0166 |
| rh_rostralanteriorcingulate_thickness | 0.0165 |
| rh_fusiform_thickness | 0.0156 |
| lh_frontalpole_thickness | 0.0151 |
| lh_parstriangularis_area | 0.0149 |
| lh_isthmuscingulate_thickness | 0.0148 |
| lh_caudalmiddlefrontal_thickness | 0.0140 |
| lh_bankssts_area | 0.0134 |
| rh_rostralmiddlefrontal_thickness | 0.0126 |
| lh_postcentral_area | 0.0103 |
| rh_middletemporal_area | 0.0087 |
| rh_medialorbitofrontal_area | 0.0065 |
| rh_entorhinal_thickness | 0.0026 |
| rh_bankssts_thickness | 0.0024 |
| lh_transversetemporal_area | 0.0006 |
| rh_cuneus_thickness | 0.0003 |
| rh_inferiorparietal_thickness | 0.0002 |
| lh_posteriorcingulate_area | 0.0002 |
| rh_temporalpole_thickness | 0.0000 |
| lh_entorhinal_area | 0.0000 |
| lh_inferiortemporal_area | 0.0000 |
| lh_medialorbitofrontal_area | 0.0000 |
| lh_middletemporal_area | 0.0000 |
| lh_parsopercularis_area | 0.0000 |
| lh_parsorbitalis_area | 0.0000 |
| lh_superiortemporal_area | 0.0000 |
| lh_frontalpole_area | 0.0000 |
| rh_caudalmiddlefrontal_area | 0.0000 |
| rh_posteriorcingulate_area | 0.0000 |
| rh_rostralanteriorcingulate_area | 0.0000 |
| rh_superiortemporal_area | 0.0000 |
| rh_supramarginal_area | 0.0000 |
| rh_transversetemporal_area | 0.0000 |
| lh_fusiform_thickness | 0.0000 |
| lh_inferiorparietal_thickness | 0.0000 |
| lh_inferiortemporal_thickness | 0.0000 |
| lh_lateralorbitofrontal_thickness | 0.0000 |
| lh_lingual_thickness | 0.0000 |
| lh_medialorbitofrontal_thickness | 0.0000 |
| lh_middletemporal_thickness | 0.0000 |
| lh_parsorbitalis_thickness | 0.0000 |
| lh_precentral_thickness | 0.0000 |
| lh_precuneus_thickness | 0.0000 |
| lh_rostralanteriorcingulate_thickness | 0.0000 |
| lh_superiorfrontal_thickness | 0.0000 |
| lh_superiorparietal_thickness | 0.0000 |
| lh_temporalpole_thickness | 0.0000 |
| rh_inferiortemporal_thickness | 0.0000 |
| rh_lateralorbitofrontal_thickness | 0.0000 |
| rh_medialorbitofrontal_thickness | 0.0000 |
| rh_middletemporal_thickness | 0.0000 |
| rh_parsopercularis_thickness | 0.0000 |
| rh_parstriangularis_thickness | 0.0000 |
| rh_postcentral_thickness | 0.0000 |
| rh_precentral_thickness | 0.0000 |
| rh_precuneus_thickness | 0.0000 |
| rh_superiorfrontal_thickness | 0.0000 |
| rh_superiorparietal_thickness | 0.0000 |
| rh_frontalpole_thickness | 0.0000 |

**Table S12b:** Classification Analysis, GLMNet Weights for Cortical Structures. The glmnet algorithm was applied to the full dataset to obtain the weights of cortical measures, which indicate the contribution of each measure in predicting the group label of a subject. Note that since glmnet performs an intrinsic feature selection with L1-norm regularization, some cortical measures had a weight of zero and therefore did not contribute in the prediction models.

**Table S13a. Demographics of Matched 22q11DS+Psychosis (N=60) and 22q11DS-No Psychosis Subjects (N=60).**

|  | **22q11DS+Psychosis** | | | | | **22q11DS-No Psychosis** | | | | |
| --- | --- | --- | --- | --- | --- | --- | --- | --- | --- | --- |
| **Site** | **N** | **N Female** | **% Female^a^** | **Mean**  **Age^b^** | **Age SD** | **N** | **N Female** | **% Female** | **Mean Age** | **Age SD** |
| **IoP** | 2 | 1 | 50 | 25.5 | 4.9 | 2 | 1 | 50 | 27.5 | 7.8 |
| **Maastricht** | 11 | 4 | 36.4 | 29.9 | 6.7 | 11 | 4 | 36.4 | 27.5 | 5.9 |
| **Newcastle** | 1 | 1 | 100 | 19 | NA | 1 | 1 | 100 | 19 | NA |
| **Penn** | 5 | 3 | 60 | 18.4 | 4.5 | 5 | 3 | 60 | 18.6 | 4.1 |
| **SUNY** | 5 | 3 | 60 | 19.4 | 1.7 | 5 | 3 | 60 | 19.4 | 1.7 |
| **Toronto1** | 6 | 3 | 50 | 39.5 | 7.6 | 6 | 3 | 50 | 42.3 | 8.8 |
| **Toronto2** | 15 | 10 | 66.7 | 29.9 | 12.1 | 15 | 10 | 66.7 | 27.3 | 9.3 |
| **UCLA1** | 2 | 1 | 50 | 18.5 | 4.9 | 2 | 1 | 50 | 19 | 7.1 |
| **UCLA2** | 3 | 2 | 66.7 | 17 | 8 | 3 | 2 | 66.7 | 16.3 | 6.8 |
| **Utrecht** | 10 | 7 | 70 | 22.7 | 5.1 | 10 | 7 | 70 | 19.5 | 3.1 |
| **Total** | 60 | 35 | 58.3 | 26.5 | 9.9 | 60 | 35 | 58.3 | 25.1 | 9.4 |

**^a .^**  Chi-square/p-value/Cramer’s V for group difference=.00/ 1.0/0

**^b.^** F/p-value/Cohen’s d for group difference= .07/0.46/.14

**Table S13a.** Demographics of Matched 22q11DS+Psychosis (N=60) and 22q11DS-No Psychosis Subjects (N=60).

**Table S13b. Psychotropic Medications, IQ and Deletion Type in 22q11DS subjects with and without Psychosis.**

| Group | 22q11DS+Psychosis | 22q11DS-No Psychosis | Chi-Square/F  (p-value) |
| --- | --- | --- | --- |
| Current Typical Antipsychotics | 11 (18.3%) | 0(0%) | 13.8 (.001) |
| Current Atypical Antipsychotics | 40 (66.7%) | 2 (3%) | 53.4 (<.001) |
| Current Anticonvulsants | 10 (16.7%) | 1 (1.7%) | 9.8 (.007) |
| Current Antidepressants | 18 (30%) | 12 (20%) | 6.5 (.37) |
| IQ (SD) | 68.02 (9.65) | 77.02 (11.64) | 20.5 (<.001) |
| A-D/A-B deletion type (%) | 90%/4.7% | 87.5%/10.4% | 5.69(.22) |

**Table S14a. Differences in CT, 22q11DS Psychosis vs. 22q11DS-No Psychosis**.

| **Region** | **Cohen d** | **Std. Err.** | **95% CI** | **%Diff** | **T-value** | **P-value** | **P-FDR** |
| --- | --- | --- | --- | --- | --- | --- | --- |
| **R pars opercularis** | -0.70 | 0.173 | -1.04, -0.36 | -3.81 | -3.99 | 1.10E-04 | 7.70E-03 |
| **R hemisphere** | -0.63 | 0.172 | -0.97, -0.29 | -2.02 | -3.61 | 4.32E-04 | 1.20E-02 |
| **R caudal middle frontal gyrus** | -0.62 | 0.172 | -0.95, -0.28 | -3.32 | -3.52 | 5.94E-04 | 1.20E-02 |
| **R postcentral gyrus** | -0.61 | 0.172 | -0.95, -0.27 | -4.05 | -3.48 | 6.88E-04 | 1.20E-02 |
| **L hemisphere** | -0.58 | 0.172 | -0.92, -0.25 | -1.89 | -3.33 | 1.14E-03 | 1.60E-02 |
| **L STG** | -0.57 | 0.172 | -0.9, -0.23 | -2.94 | -3.23 | 1.55E-03 | 1.62E-02 |
| **L pars opercularis** | -0.56 | 0.171 | -0.89, -0.22 | -3.09 | -3.18 | 1.84E-03 | 1.62E-02 |
| **R superior frontal gyrus** | -0.56 | 0.171 | -0.89, -0.22 | -2.56 | -3.18 | 1.85E-03 | 1.62E-02 |
| **R STG** | -0.53 | 0.171 | -0.87, -0.2 | -2.62 | -3.03 | 2.94E-03 | 2.28E-02 |
| **R precentral gyrus** | -0.52 | 0.171 | -0.86, -0.19 | -2.44 | -3.00 | 3.26E-03 | 2.28E-02 |
| **R rostral middle frontal gyrus** | -0.52 | 0.171 | -0.85, -0.18 | -2.69 | -2.95 | 3.71E-03 | 2.34E-02 |
| **L superior frontal gyrus** | -0.51 | 0.171 | -0.85, -0.18 | -2.36 | -2.93 | 4.01E-03 | 2.34E-02 |
| **R supramarginal gyrus** | -0.49 | 0.171 | -0.83, -0.16 | -2.30 | -2.83 | 5.42E-03 | 2.92E-02 |
| **R transverse temporal gyrus** | -0.49 | 0.171 | -0.82, -0.15 | -4.30 | -2.79 | 6.05E-03 | 3.03E-02 |
| **L precentral gyrus** | -0.48 | 0.171 | -0.82, -0.15 | -2.27 | -2.76 | 6.54E-03 | 3.05E-02 |
| **L rostral middle frontal gyrus** | -0.48 | 0.171 | -0.81, -0.15 | -2.25 | -2.74 | 6.99E-03 | 3.06E-02 |
| **L inferior temporal gyrus** | -0.47 | 0.171 | -0.81, -0.14 | -2.59 | -2.71 | 7.70E-03 | 3.17E-02 |
| **R inferior temporal gyrus** | -0.45 | 0.170 | -0.78, -0.12 | -2.50 | -2.57 | 1.13E-02 | 4.40E-02 |
| **L postcentral gyrus** | -0.45 | 0.170 | -0.78, -0.11 | -2.41 | -2.54 | 1.21E-02 | 4.47E-02 |
| **L entorhinal cortex** | -0.39 | 0.170 | -0.72, -0.06 | -3.92 | -2.22 | 2.79E-02 | 9.67E-02 |
| **L transverse temporal gyrus** | -0.39 | 0.170 | -0.72, -0.05 | -3.64 | -2.21 | 2.90E-02 | 9.67E-02 |
| **R inferior parietal cortex** | -0.38 | 0.170 | -0.71, -0.05 | -1.58 | -2.16 | 3.25E-02 | 9.92E-02 |
| **L supramarginal gyrus** | -0.38 | 0.170 | -0.71, -0.05 | -1.70 | -2.16 | 3.26E-02 | 9.92E-02 |
| **R superior parietal cortex** | -0.37 | 0.170 | -0.7, -0.03 | -1.67 | -2.09 | 3.81E-02 | 1.09E-01 |
| **L insula** | -0.36 | 0.170 | -0.7, -0.03 | -1.74 | -2.08 | 3.98E-02 | 1.09E-01 |
| **L middle temporal gyrus** | -0.36 | 0.170 | -0.69, -0.03 | -1.80 | -2.07 | 4.05E-02 | 1.09E-01 |
| **R pars triangularis** | -0.35 | 0.169 | -0.68, -0.02 | -2.07 | -1.99 | 4.89E-02 | 1.20E-01 |
| **R pars orbitalis** | -0.35 | 0.169 | -0.68, -0.01 | -2.29 | -1.98 | 4.96E-02 | 1.20E-01 |
| **R lateral occipital cortex** | -0.35 | 0.169 | -0.68, -0.01 | -1.80 | -1.97 | 5.07E-02 | 1.20E-01 |
| **R paracentral lobule** | -0.34 | 0.169 | -0.68, -0.01 | -1.89 | -1.97 | 5.13E-02 | 1.20E-01 |
| **R middle temporal gyrus** | -0.34 | 0.169 | -0.67, -0.01 | -1.97 | -1.95 | 5.36E-02 | 1.21E-01 |
| **L inferior parietal cortex** | -0.34 | 0.169 | -0.67, 0 | -1.48 | -1.92 | 5.67E-02 | 1.24E-01 |
| **L banks of STS** | -0.33 | 0.169 | -0.66, 0 | -2.13 | -1.87 | 6.36E-02 | 1.35E-01 |
| **L precuneus** | -0.31 | 0.169 | -0.64, 0.02 | -1.50 | -1.78 | 7.78E-02 | 1.60E-01 |
| **L caudal middle frontal gyrus** | -0.31 | 0.169 | -0.64, 0.03 | -1.57 | -1.75 | 8.31E-02 | 1.66E-01 |
| **L pars orbitalis** | -0.30 | 0.169 | -0.63, 0.03 | -2.08 | -1.71 | 8.92E-02 | 1.73E-01 |
| **L paracentral lobule** | -0.29 | 0.169 | -0.62, 0.04 | -1.62 | -1.66 | 1.00E-01 | 1.81E-01 |
| **R fusiform gyrus** | -0.29 | 0.169 | -0.62, 0.04 | -1.51 | -1.65 | 1.00E-01 | 1.81E-01 |
| **L frontal pole** | -0.29 | 0.169 | -0.62, 0.04 | -3.33 | -1.65 | 1.01E-01 | 1.81E-01 |
| **R insula** | -0.28 | 0.169 | -0.61, 0.05 | -1.48 | -1.58 | 1.17E-01 | 2.04E-01 |
| **L pars triangularis** | -0.27 | 0.169 | -0.6, 0.06 | -1.52 | -1.56 | 1.22E-01 | 2.08E-01 |
| **L lateral occipital cortex** | -0.26 | 0.169 | -0.59, 0.08 | -1.41 | -1.46 | 1.47E-01 | 2.39E-01 |
| **L fusiform gyrus** | -0.25 | 0.169 | -0.58, 0.08 | -1.33 | -1.45 | 1.50E-01 | 2.39E-01 |
| **L lateral orbitofrontal cortex** | -0.25 | 0.169 | -0.58, 0.08 | -1.50 | -1.45 | 1.51E-01 | 2.39E-01 |
| **R lateral orbitofrontal cortex** | -0.23 | 0.169 | -0.56, 0.1 | -1.31 | -1.31 | 1.92E-01 | 2.99E-01 |
| **R medial orbitofrontal cortex** | -0.22 | 0.169 | -0.55, 0.11 | -1.45 | -1.28 | 2.04E-01 | 3.10E-01 |
| **L medial orbitofrontal cortex** | -0.21 | 0.169 | -0.54, 0.12 | -1.32 | -1.20 | 2.31E-01 | 3.44E-01 |
| **R caudal anterior cingulate** | -0.21 | 0.169 | -0.54, 0.12 | -2.00 | -1.19 | 2.36E-01 | 3.44E-01 |
| **L rostral anterior cingulate** | -0.20 | 0.169 | -0.53, 0.13 | -1.33 | -1.17 | 2.46E-01 | 3.51E-01 |
| **R rostral anterior cingulate** | -0.19 | 0.169 | -0.52, 0.14 | -1.40 | -1.11 | 2.68E-01 | 3.76E-01 |
| **L superior parietal cortex** | -0.19 | 0.169 | -0.52, 0.14 | -0.87 | -1.06 | 2.89E-01 | 3.97E-01 |
| **L temporal pole** | -0.14 | 0.168 | -0.47, 0.19 | -1.78 | -0.82 | 4.16E-01 | 5.53E-01 |
| **R banks of STS** | -0.14 | 0.168 | -0.47, 0.19 | -0.92 | -0.80 | 4.27E-01 | 5.53E-01 |
| **L isthmus cingulate** | -0.14 | 0.168 | -0.47, 0.19 | -1.01 | -0.80 | 4.27E-01 | 5.53E-01 |
| **R entorhinal cortex** | -0.13 | 0.168 | -0.46, 0.2 | -1.48 | -0.73 | 4.65E-01 | 5.92E-01 |
| **L posterior cingulate** | -0.12 | 0.168 | -0.45, 0.21 | -0.65 | -0.68 | 4.98E-01 | 6.04E-01 |
| **R posterior cingulate** | -0.12 | 0.168 | -0.45, 0.21 | -0.66 | -0.68 | 5.00E-01 | 6.04E-01 |
| **R parahippocampal gyrus** | -0.12 | 0.168 | -0.45, 0.21 | -1.05 | -0.67 | 5.02E-01 | 6.04E-01 |
| **R frontal pole** | -0.08 | 0.168 | -0.41, 0.25 | -0.88 | -0.44 | 6.64E-01 | 7.38E-01 |
| **R pericalcarine cortex** | -0.06 | 0.168 | -0.39, 0.27 | -0.45 | -0.34 | 7.35E-01 | 8.04E-01 |
| **R temporal pole** | -0.05 | 0.168 | -0.38, 0.28 | -0.52 | -0.26 | 7.92E-01 | 8.53E-01 |
| **L caudal anterior cingulate** | -0.04 | 0.168 | -0.37, 0.29 | -0.38 | -0.24 | 8.11E-01 | 8.60E-01 |
| **L cuneus** | -0.03 | 0.168 | -0.36, 0.3 | -0.20 | -0.16 | 8.73E-01 | 8.99E-01 |
| **R cuneus** | 0.00 | 0.168 | -0.33, 0.33 | -0.02 | -0.02 | 9.87E-01 | 9.87E-01 |
| **R precuneus** | 0.01 | 0.168 | -0.32, 0.34 | 0.06 | 0.08 | 9.35E-01 | 9.48E-01 |
| **L pericalcarine cortex** | 0.03 | 0.168 | -0.3, 0.36 | 0.23 | 0.17 | 8.69E-01 | 8.99E-01 |
| **L parahippocampal gyrus** | 0.08 | 0.168 | -0.25, 0.41 | 0.75 | 0.45 | 6.50E-01 | 7.34E-01 |
| **R lingual gyrus** | 0.11 | 0.168 | -0.22, 0.44 | 0.61 | 0.62 | 5.39E-01 | 6.19E-01 |
| **L lingual gyrus** | 0.11 | 0.168 | -0.22, 0.44 | 0.57 | 0.64 | 5.24E-01 | 6.12E-01 |
| **R isthmus cingulate** | 0.12 | 0.168 | -0.21, 0.45 | 0.70 | 0.66 | 5.09E-01 | 6.04E-01 |

**Table S14a**. Differences in Cortical Thickness, 22q11DS Psychosis vs. 22q11DS-No Psychosis: Effect size (Cohen’s d), 95% Confidence Interval (CI), Percent Difference, and P-Value. The following linear model was used for the group comparison: CT ~ Group + Site + Sex + Age. Df=107 for all comparisons.

**Table S14b. Differences in SA, 22q11DS Psychosis vs. 22q11DS-No Psychosis.**

| **Region** | **Cohen d** | **Std. Err.** | **95% CI** | **% Diff** | **T-value** | **P-value** |
| --- | --- | --- | --- | --- | --- | --- |
| **L middle temporal gyrus** | -0.355 | 0.184 | -0.72, 0.01 | -8.485 | -1.826 | 0.071 |
| **R parahippocampal gyrus** | -0.328 | 0.184 | -0.69, 0.03 | -6.036 | -1.687 | 0.094 |
| **L entorhinal cortex** | -0.286 | 0.184 | -0.65, 0.07 | -7.033 | -1.470 | 0.145 |
| **R middle temporal gyrus** | -0.284 | 0.184 | -0.64, 0.08 | -5.097 | -1.461 | 0.147 |
| **R rostral anterior cingulate** | -0.263 | 0.183 | -0.62, 0.1 | -12.168 | -1.355 | 0.178 |
| **R inferior parietal cortex** | -0.251 | 0.183 | -0.61, 0.11 | -7.934 | -1.292 | 0.199 |
| **R inferior temporal gyrus** | -0.244 | 0.183 | -0.6, 0.12 | -4.983 | -1.256 | 0.212 |
| **L caudal anterior cingulate** | -0.213 | 0.183 | -0.57, 0.15 | -6.023 | -1.096 | 0.276 |
| **R caudal anterior cingulate** | -0.208 | 0.183 | -0.57, 0.15 | -7.167 | -1.070 | 0.287 |
| **R medial orbitofrontal cortex** | -0.185 | 0.183 | -0.54, 0.17 | -4.617 | -0.951 | 0.344 |
| **R postcentral gyrus** | -0.184 | 0.183 | -0.54, 0.17 | -3.331 | -0.948 | 0.345 |
| **R pars orbitalis** | -0.173 | 0.183 | -0.53, 0.19 | -3.138 | -0.892 | 0.374 |
| **L inferior temporal gyrus** | -0.172 | 0.183 | -0.53, 0.19 | -4.802 | -0.887 | 0.377 |
| **L medial orbitofrontal cortex** | -0.160 | 0.183 | -0.52, 0.2 | -2.963 | -0.822 | 0.413 |
| **R fusiform gyrus** | -0.142 | 0.183 | -0.5, 0.22 | -2.975 | -0.731 | 0.466 |
| **L rostral anterior cingulate** | -0.127 | 0.183 | -0.49, 0.23 | -20.143 | -0.656 | 0.513 |
| **R posterior cingulate** | -0.120 | 0.183 | -0.48, 0.24 | -5.603 | -0.617 | 0.538 |
| **L parahippocampal gyrus** | -0.105 | 0.183 | -0.46, 0.25 | -1.828 | -0.539 | 0.591 |
| **L posterior cingulate** | -0.094 | 0.183 | -0.45, 0.26 | -2.588 | -0.483 | 0.630 |
| **L inferior parietal cortex** | -0.078 | 0.183 | -0.44, 0.28 | -1.899 | -0.401 | 0.689 |
| **R lateral orbitofrontal cortex** | -0.076 | 0.183 | -0.43, 0.28 | -1.790 | -0.390 | 0.697 |
| **L precentral gyrus** | -0.069 | 0.183 | -0.43, 0.29 | -1.187 | -0.355 | 0.723 |
| **R banks of STS** | -0.060 | 0.183 | -0.42, 0.3 | -2.376 | -0.307 | 0.759 |
| **L frontal pole** | -0.059 | 0.183 | -0.42, 0.3 | -1.105 | -0.304 | 0.762 |
| **R rostral middle frontal gyrus** | -0.050 | 0.183 | -0.41, 0.31 | -0.882 | -0.256 | 0.799 |
| **L banks of STS** | -0.039 | 0.183 | -0.4, 0.32 | -1.020 | -0.202 | 0.840 |
| **L pars orbitalis** | -0.039 | 0.183 | -0.4, 0.32 | -0.657 | -0.202 | 0.841 |
| **L lateral orbitofrontal cortex** | -0.037 | 0.183 | -0.39, 0.32 | -0.896 | -0.190 | 0.850 |
| **R paracentral lobule** | -0.013 | 0.183 | -0.37, 0.34 | -0.321 | -0.066 | 0.947 |
| **L supramarginal gyrus** | 0.004 | 0.183 | -0.35, 0.36 | 0.165 | 0.022 | 0.982 |
| **R caudal middle frontal gyrus** | 0.016 | 0.183 | -0.34, 0.37 | 0.760 | 0.084 | 0.933 |
| **L cuneus** | 0.039 | 0.183 | -0.32, 0.4 | 0.880 | 0.200 | 0.842 |
| **R hemisphere** | 0.043 | 0.183 | -0.31, 0.4 | 0.609 | 0.224 | 0.823 |
| **L caudal middle frontal gyrus** | 0.046 | 0.183 | -0.31, 0.4 | 1.461 | 0.238 | 0.813 |
| **L STG** | 0.068 | 0.183 | -0.29, 0.43 | 1.377 | 0.348 | 0.728 |
| **R supramarginal gyrus** | 0.070 | 0.183 | -0.29, 0.43 | 1.745 | 0.359 | 0.720 |
| **R isthmus cingulate** | 0.073 | 0.183 | -0.29, 0.43 | 3.217 | 0.373 | 0.710 |
| **R superior frontal gyrus** | 0.084 | 0.183 | -0.27, 0.44 | 2.177 | 0.433 | 0.666 |
| **R precuneus** | 0.085 | 0.183 | -0.27, 0.44 | 1.891 | 0.437 | 0.663 |
| **L fusiform gyrus** | 0.090 | 0.183 | -0.27, 0.45 | 2.064 | 0.463 | 0.644 |
| **R entorhinal cortex** | 0.095 | 0.183 | -0.26, 0.45 | 7.713 | 0.487 | 0.627 |
| **L transverse temporal gyrus** | 0.104 | 0.183 | -0.25, 0.46 | 3.231 | 0.536 | 0.593 |
| **R frontal pole** | 0.109 | 0.183 | -0.25, 0.47 | 2.219 | 0.563 | 0.574 |
| **R insula** | 0.120 | 0.183 | -0.24, 0.48 | 3.191 | 0.618 | 0.538 |
| **L postcentral gyrus** | 0.126 | 0.183 | -0.23, 0.48 | 2.856 | 0.646 | 0.520 |
| **L hemisphere** | 0.126 | 0.183 | -0.23, 0.48 | 1.735 | 0.646 | 0.519 |
| **R pericalcarine cortex** | 0.134 | 0.183 | -0.22, 0.49 | 3.007 | 0.687 | 0.493 |
| **L pars opercularis** | 0.144 | 0.183 | -0.21, 0.5 | 4.686 | 0.744 | 0.459 |
| **L lingual gyrus** | 0.159 | 0.183 | -0.2, 0.52 | 2.649 | 0.818 | 0.415 |
| **L isthmus cingulate** | 0.166 | 0.183 | -0.19, 0.52 | 6.239 | 0.856 | 0.394 |
| **L pericalcarine cortex** | 0.168 | 0.183 | -0.19, 0.53 | 4.156 | 0.865 | 0.389 |
| **L temporal pole** | 0.172 | 0.183 | -0.19, 0.53 | 3.654 | 0.883 | 0.379 |
| **L superior frontal gyrus** | 0.181 | 0.183 | -0.18, 0.54 | 4.115 | 0.932 | 0.353 |
| **R pars opercularis** | 0.184 | 0.183 | -0.17, 0.54 | 6.715 | 0.950 | 0.344 |
| **R precentral gyrus** | 0.185 | 0.183 | -0.17, 0.54 | 3.174 | 0.951 | 0.344 |
| **R STG** | 0.186 | 0.183 | -0.17, 0.54 | 4.414 | 0.958 | 0.340 |
| **L lateral occipital cortex** | 0.201 | 0.183 | -0.16, 0.56 | 4.260 | 1.034 | 0.304 |
| **R pars triangularis** | 0.233 | 0.183 | -0.13, 0.59 | 5.901 | 1.201 | 0.233 |
| **L paracentral lobule** | 0.240 | 0.183 | -0.12, 0.6 | 13.024 | 1.233 | 0.220 |
| **L precuneus** | 0.240 | 0.183 | -0.12, 0.6 | 4.414 | 1.235 | 0.220 |
| **R lingual gyrus** | 0.244 | 0.183 | -0.12, 0.6 | 5.075 | 1.255 | 0.212 |
| **L superior parietal cortex** | 0.246 | 0.183 | -0.11, 0.61 | 5.369 | 1.265 | 0.209 |
| **R temporal pole** | 0.258 | 0.183 | -0.1, 0.62 | 7.201 | 1.326 | 0.188 |
| **R superior parietal cortex** | 0.304 | 0.184 | -0.06, 0.66 | 6.204 | 1.566 | 0.120 |
| **L rostral middle frontal gyrus** | 0.310 | 0.184 | -0.05, 0.67 | 5.685 | 1.594 | 0.114 |
| **R lateral occipital cortex** | 0.311 | 0.184 | -0.05, 0.67 | 6.608 | 1.600 | 0.112 |
| **L pars triangularis** | 0.347 | 0.184 | -0.01, 0.71 | 7.920 | 1.785 | 0.077 |
| **L insula** | 0.362 | 0.184 | 0, 0.72 | 17.038 | 1.864 | 0.065 |
| **R transverse temporal gyrus** | 0.444 | 0.185 | 0.08, 0.81 | 10.672 | 2.285 | 0.024 |
| **R cuneus** | 0.453 | 0.185 | 0.09, 0.82 | 11.595 | 2.333 | 0.022 |

**Table S14b.** Differences in Surface Area, 22q11DS Psychosis vs. 22q11DS-No Psychosis: Effect size (Cohen’s d), 95% Confidence Interval (CI), Percent Difference, and P-Value. The following linear model was used for the group comparison: SA ~ Group + Site + Sex + Age + ICV. Df= 106 for all comparisons.

# **Table S15a. Demographics of Matched A-B and A-D Deletion and Control Subjects**

|  | **A-B Deletion** | | | | **A-D Deletion** | | | | **Control** | | | |
| --- | --- | --- | --- | --- | --- | --- | --- | --- | --- | --- | --- | --- |
| **Site** | **N** | **N Female^a^** | **Mean Age^b^** | **Age SD** | **N** | **N Female** | **Mean Age** | **Age SD** | **N** | **N Female** | **Mean Age** | **Age SD** |
| **Maastricht** | 4 | 2 | 32.8 | 8.8 | 15 | 9 | 30.9 | 6.4 | 20 | 10 | 30.3 | 8.3 |
| **Penn** | 5 | 1 | 17.4 | 3.4 | 25 | 5 | 17.5 | 3 | 25 | 5 | 17.5 | 3 |
| **SUNY** | 1 | 0 | 22 | NA | 5 | 0 | 21.8 | 1.3 | 5 | 0 | 21.8 | 0.4 |
| **Toronto1** | 1 | 0 | 34 | NA | 5 | 0 | 40.2 | 3.9 | 5 | 0 | 40.8 | 3.8 |
| **Toronto2** | 3 | 3 | 27.7 | 7.6 | 15 | 15 | 28 | 8.5 | 0 | 0 | NA | NA |
| **UCDavis1** | 1 | 0 | 11 | NA | 5 | 0 | 10.8 | 0.8 | 5 | 0 | 11 | 1 |
| **UCDavis2** | 1 | 0 | 8 | NA | 5 | 0 | 8.4 | 0.5 | 5 | 0 | 8 | 0 |
| **UCLA1** | 3 | 1 | 8 | 3.5 | 15 | 5 | 12.1 | 4.7 | 12 | 5 | 11.3 | 5.2 |
| **UCLA2** | 2 | 0 | 9.5 | 4.9 | 10 | 0 | 9.6 | 3.9 | 10 | 0 | 11.8 | 2.2 |
| **Utrecht** | 2 | 0 | 15 | 0 | 8 | 0 | 17.9 | 2.4 | 0 | 0 | NA | NA |
| **Total** | 23 | 7 | 18.6 | 9.9 | 108 | 34 | 18.7 | 11.0 | 87 | 20 | 19.5 | 10.3 |

**^a^** Chi square/p-value/ Cramer’s V for group difference= 1.8/.41/.09

**^b^** F/p-value/Cohen’s d for group difference= .022/0.98/.00

# **Table S15b. Psychotropic Medications, IQ and Psychosis in 22q11DS Subjects with A-B vs. A-D Deletions.**

| Group | A-D | A-B | Chi-Square/F  (p-value) |
| --- | --- | --- | --- |
| Current Typical Antipsychotics | 3 (2.8%) | 2(8.7%) | 4.37 (0.11) |
| Current Atypical Antipsychotics | 18 (16.7%) | 2 (8.7%) | 2.99 (0.23) |
| Current Anticonvulsants | 6 (5.6%) | 2 (8.7%) | 2.8 (.25) |
| Current Antidepressants | 26 (24.0%) | 3 (13%) | 4.5 (.34) |
| IQ (SD) | 75.77 (12.4) | 82.14 (9.3) | 4.93 (.03) |
| N (%)Psychotic Disorder | 2 (8.7%) | 23 (21.3%) | 1.95(0.24) |

# **Table S16a.** Comparison of Cortical Thickness Differences among Subjects with A-D deletions, A-B Deletions and Controls.

| Region | p-value  3 groups | t-value  AD>AB | p-value  AD>AB | p-FDR  AD>AB | t-value  CL>AB | p-value  CL>AB | p-FDR  CL>AB | t-value  CL>AD | p-value  CL>AD | p-FDR  CL>AD |
| --- | --- | --- | --- | --- | --- | --- | --- | --- | --- | --- |
| R pericalcarine cortex | 9.88E-08 | -1.15 | 4.77E-01 | 1.00E+00 | -4.44 | 4.04E-05 | 2.83E-03 | -5.29 | 9.00E-07 | 3.15E-05 |
| L parahippocampal gyrus | 1.15E-06 | -1.86 | 1.49E-01 | 1.00E+00 | 1.62 | 2.33E-01 | 6.27E-01 | 5.39 | 5.05E-07 | 3.15E-05 |
| R rostral middle frontal gyrus | 3.20E-06 | 0.73 | 7.40E-01 | 1.00E+00 | -2.55 | 2.97E-02 | 1.66E-01 | -5.15 | 1.92E-06 | 4.29E-05 |
| L pericalcarine cortex | 5.10E-06 | -1.46 | 3.08E-01 | 1.00E+00 | -4.12 | 1.54E-04 | 5.37E-03 | -4.31 | 7.66E-05 | 5.96E-04 |
| R caudal middle frontal gyrus | 5.42E-06 | 1.55 | 2.63E-01 | 1.00E+00 | -1.71 | 1.97E-01 | 5.52E-01 | -5.07 | 2.45E-06 | 4.29E-05 |
| R lingual gyrus | 5.70E-06 | -0.45 | 8.94E-01 | 1.00E+00 | -3.42 | 2.05E-03 | 3.59E-02 | -4.74 | 1.12E-05 | 1.31E-04 |
| L STG | 1.43E-05 | -0.73 | 7.43E-01 | 1.00E+00 | 2.35 | 4.98E-02 | 1.94E-01 | 4.82 | 7.85E-06 | 1.10E-04 |
| R supramarginal gyrus | 3.44E-05 | 2.11 | 8.63E-02 | 1.00E+00 | -0.85 | 6.69E-01 | 8.95E-01 | -4.56 | 2.65E-05 | 2.65E-04 |
| L medial orbitofrontal cortex | 5.00E-05 | 1.98 | 1.15E-01 | 1.00E+00 | -0.93 | 6.16E-01 | 8.79E-01 | -4.49 | 3.56E-05 | 3.12E-04 |
| L rostral middle frontal gyrus | 1.59E-04 | -0.02 | 1.00E+00 | 1.00E+00 | -2.61 | 2.48E-02 | 1.58E-01 | -4.12 | 1.57E-04 | 1.00E-03 |
| L cuneus | 2.38E-04 | -1.52 | 2.78E-01 | 1.00E+00 | -3.57 | 1.24E-03 | 2.89E-02 | -3.34 | 2.72E-03 | 7.35E-03 |
| R hemisphere | 2.70E-04 | 0.80 | 7.00E-01 | 1.00E+00 | -1.84 | 1.54E-01 | 4.69E-01 | -4.13 | 1.53E-04 | 1.00E-03 |
| L pars opercularis | 2.79E-04 | -0.71 | 7.56E-01 | 1.00E+00 | -3.04 | 7.25E-03 | 8.46E-02 | -3.73 | 6.83E-04 | 2.40E-03 |
| R paracentral lobule | 4.97E-04 | -0.56 | 8.38E-01 | 1.00E+00 | -2.84 | 1.31E-02 | 1.15E-01 | -3.64 | 9.59E-04 | 3.20E-03 |
| L insula | 5.22E-04 | -0.12 | 9.92E-01 | 1.00E+00 | -2.51 | 3.32E-02 | 1.66E-01 | -3.79 | 5.71E-04 | 2.40E-03 |
| R insula | 5.31E-04 | 1.56 | 2.57E-01 | 1.00E+00 | -0.97 | 5.89E-01 | 8.59E-01 | -3.92 | 3.33E-04 | 1.95E-03 |
| R cuneus | 6.09E-04 | -0.16 | 9.85E-01 | 1.00E+00 | -2.51 | 3.25E-02 | 1.66E-01 | -3.73 | 6.85E-04 | 2.40E-03 |
| L caudal middle frontal gyrus | 7.11E-04 | -0.01 | 1.00E+00 | 1.00E+00 | -2.37 | 4.74E-02 | 1.94E-01 | -3.73 | 6.79E-04 | 2.40E-03 |
| R precentral gyrus | 7.37E-04 | 1.78 | 1.73E-01 | 1.00E+00 | -0.67 | 7.74E-01 | 8.98E-01 | -3.78 | 5.75E-04 | 2.40E-03 |
| L hemisphere | 7.58E-04 | 0.38 | 9.20E-01 | 1.00E+00 | -2.03 | 1.03E-01 | 3.45E-01 | -3.80 | 5.20E-04 | 2.40E-03 |
| L supramarginal gyrus | 9.41E-04 | 1.17 | 4.65E-01 | 1.00E+00 | -1.27 | 4.05E-01 | 7.46E-01 | -3.79 | 5.41E-04 | 2.40E-03 |
| L postcentral gyrus | 1.00E-03 | 0.47 | 8.81E-01 | 1.00E+00 | -1.91 | 1.35E-01 | 4.29E-01 | -3.74 | 6.60E-04 | 2.40E-03 |
| L pars triangularis | 1.26E-03 | -1.39 | 3.39E-01 | 1.00E+00 | -3.20 | 4.29E-03 | 6.00E-02 | -2.95 | 9.42E-03 | 2.00E-02 |
| R superior frontal gyrus | 1.60E-03 | 0.12 | 9.92E-01 | 1.00E+00 | -2.12 | 8.48E-02 | 3.06E-01 | -3.54 | 1.37E-03 | 4.35E-03 |
| L lingual gyrus | 1.78E-03 | -0.75 | 7.31E-01 | 1.00E+00 | -2.75 | 1.72E-02 | 1.34E-01 | -3.21 | 4.18E-03 | 9.93E-03 |
| R postcentral gyrus | 2.76E-03 | 1.26 | 4.11E-01 | 1.00E+00 | -0.98 | 5.86E-01 | 8.59E-01 | -3.47 | 1.79E-03 | 5.28E-03 |
| L lateral orbitofrontal cortex | 2.91E-03 | 1.14 | 4.85E-01 | 1.00E+00 | -1.09 | 5.13E-01 | 8.58E-01 | -3.46 | 1.81E-03 | 5.28E-03 |
| R precuneus | 2.97E-03 | 0.00 | 1.00E+00 | 1.00E+00 | -2.11 | 8.75E-02 | 3.06E-01 | -3.34 | 2.77E-03 | 7.35E-03 |
| R medial orbitofrontal cortex | 4.10E-03 | 1.32 | 3.77E-01 | 1.00E+00 | -0.83 | 6.77E-01 | 8.95E-01 | -3.33 | 2.84E-03 | 7.35E-03 |
| R superior parietal cortex | 4.52E-03 | -0.90 | 6.33E-01 | 1.00E+00 | -2.67 | 2.14E-02 | 1.50E-01 | -2.85 | 1.27E-02 | 2.55E-02 |
| R parahippocampal gyrus | 4.99E-03 | -1.08 | 5.18E-01 | 1.00E+00 | 1.04 | 5.47E-01 | 8.59E-01 | 3.29 | 3.21E-03 | 8.03E-03 |
| L paracentral lobule | 5.20E-03 | -0.50 | 8.68E-01 | 1.00E+00 | -2.37 | 4.65E-02 | 1.94E-01 | -3.00 | 8.18E-03 | 1.85E-02 |
| L precentral gyrus | 6.44E-03 | 0.60 | 8.17E-01 | 1.00E+00 | -1.45 | 3.11E-01 | 6.88E-01 | -3.21 | 4.25E-03 | 9.93E-03 |
| L superior frontal gyrus | 1.12E-02 | 0.30 | 9.50E-01 | 1.00E+00 | -1.60 | 2.43E-01 | 6.31E-01 | -2.99 | 8.46E-03 | 1.85E-02 |
| R middle temporal gyrus | 1.22E-02 | 1.87 | 1.45E-01 | 1.00E+00 | 0.06 | 9.98E-01 | 1.00E+00 | -2.76 | 1.66E-02 | 3.14E-02 |
| R pars triangularis | 1.56E-02 | 0.86 | 6.63E-01 | 1.00E+00 | -1.02 | 5.60E-01 | 8.59E-01 | -2.91 | 1.06E-02 | 2.18E-02 |
| R temporal pole | 1.84E-02 | 2.22 | 6.71E-02 | 1.00E+00 | 2.85 | 1.26E-02 | 1.15E-01 | 1.14 | 4.86E-01 | 5.86E-01 |
| L precuneus | 2.07E-02 | 1.05 | 5.42E-01 | 1.00E+00 | -0.76 | 7.23E-01 | 8.98E-01 | -2.79 | 1.50E-02 | 2.92E-02 |
| R STG | 3.24E-02 | -0.36 | 9.28E-01 | 1.00E+00 | 1.30 | 3.87E-01 | 7.46E-01 | 2.62 | 2.45E-02 | 4.51E-02 |
| L pars orbitalis | 3.66E-02 | 0.53 | 8.54E-01 | 1.00E+00 | -1.13 | 4.92E-01 | 8.58E-01 | -2.59 | 2.66E-02 | 4.78E-02 |
| L middle temporal gyrus | 5.15E-02 | 0.67 | 7.80E-01 | 1.00E+00 | -0.91 | 6.28E-01 | 8.80E-01 | -2.45 | 3.80E-02 | 6.65E-02 |
| L temporal pole | 5.77E-02 | 1.57 | 2.56E-01 | 1.00E+00 | 2.35 | 4.89E-02 | 1.94E-01 | 1.34 | 3.65E-01 | 4.74E-01 |
| L inferior temporal gyrus | 6.53E-02 | 2.13 | 8.39E-02 | 1.00E+00 | 1.07 | 5.24E-01 | 8.58E-01 | -1.53 | 2.70E-01 | 3.76E-01 |
| R lateral orbitofrontal cortex | 7.13E-02 | 0.69 | 7.66E-01 | 1.00E+00 | -0.80 | 6.98E-01 | 8.98E-01 | -2.31 | 5.42E-02 | 9.25E-02 |
| R pars opercularis | 7.19E-02 | -0.11 | 9.94E-01 | 1.00E+00 | -1.49 | 2.91E-01 | 6.79E-01 | -2.20 | 7.15E-02 | 1.19E-01 |
| L caudal anterior cingulate | 8.76E-02 | -1.32 | 3.80E-01 | 1.00E+00 | 0.04 | 9.99E-01 | 1.00E+00 | 2.07 | 9.45E-02 | 1.50E-01 |
| L superior parietal cortex | 8.91E-02 | -0.03 | 9.99E-01 | 1.00E+00 | -1.37 | 3.50E-01 | 7.42E-01 | -2.12 | 8.44E-02 | 1.37E-01 |
| L fusiform gyrus | 9.03E-02 | 1.93 | 1.29E-01 | 1.00E+00 | 0.88 | 6.48E-01 | 8.89E-01 | -1.54 | 2.66E-01 | 3.76E-01 |
| R frontal pole | 1.59E-01 | -1.93 | 1.30E-01 | 1.00E+00 | -1.49 | 2.91E-01 | 6.79E-01 | 0.57 | 8.31E-01 | 9.09E-01 |
| L isthmus cingulate | 1.62E-01 | 0.12 | 9.92E-01 | 1.00E+00 | -1.07 | 5.27E-01 | 8.58E-01 | -1.88 | 1.44E-01 | 2.18E-01 |
| R pars orbitalis | 1.73E-01 | 0.52 | 8.59E-01 | 1.00E+00 | -0.69 | 7.66E-01 | 8.98E-01 | -1.88 | 1.42E-01 | 2.18E-01 |
| R entorhinal cortex | 1.77E-01 | 0.98 | 5.86E-01 | 1.00E+00 | 1.74 | 1.86E-01 | 5.42E-01 | 1.28 | 4.03E-01 | 5.05E-01 |
| L posterior cingulate | 1.84E-01 | 0.28 | 9.57E-01 | 1.00E+00 | 1.33 | 3.71E-01 | 7.44E-01 | 1.69 | 2.07E-01 | 3.09E-01 |
| R lateral occipital cortex | 2.26E-01 | -0.38 | 9.21E-01 | 1.00E+00 | -1.33 | 3.72E-01 | 7.44E-01 | -1.53 | 2.74E-01 | 3.76E-01 |
| R caudal anterior cingulate | 2.77E-01 | -0.37 | 9.26E-01 | 1.00E+00 | 0.66 | 7.82E-01 | 8.98E-01 | 1.61 | 2.39E-01 | 3.48E-01 |
| L inferior parietal cortex | 2.90E-01 | -0.90 | 6.35E-01 | 1.00E+00 | -1.50 | 2.84E-01 | 6.79E-01 | -1.01 | 5.63E-01 | 6.56E-01 |
| L transverse temporal gyrus | 3.05E-01 | -0.97 | 5.88E-01 | 1.00E+00 | -0.04 | 9.99E-01 | 1.00E+00 | 1.42 | 3.28E-01 | 4.41E-01 |
| R isthmus cingulate | 3.36E-01 | 0.94 | 6.11E-01 | 1.00E+00 | 1.44 | 3.15E-01 | 6.88E-01 | 0.86 | 6.62E-01 | 7.48E-01 |
| R fusiform gyrus | 3.47E-01 | 1.32 | 3.80E-01 | 1.00E+00 | 0.66 | 7.81E-01 | 8.98E-01 | -0.96 | 5.99E-01 | 6.88E-01 |
| L lateral occipital cortex | 3.86E-01 | 0.07 | 9.97E-01 | 1.00E+00 | -0.78 | 7.08E-01 | 8.98E-01 | -1.35 | 3.61E-01 | 4.74E-01 |
| L entorhinal cortex | 4.40E-01 | 0.98 | 5.80E-01 | 1.00E+00 | 1.28 | 3.99E-01 | 7.46E-01 | 0.53 | 8.53E-01 | 9.14E-01 |
| R posterior cingulate | 4.45E-01 | -0.40 | 9.12E-01 | 1.00E+00 | 0.42 | 9.07E-01 | 9.92E-01 | 1.27 | 4.04E-01 | 5.05E-01 |
| L banks of STS | 4.57E-01 | -0.97 | 5.92E-01 | 1.00E+00 | -1.25 | 4.16E-01 | 7.47E-01 | -0.51 | 8.62E-01 | 9.14E-01 |
| R inferior temporal gyrus | 4.98E-01 | 1.13 | 4.92E-01 | 1.00E+00 | 0.67 | 7.76E-01 | 8.98E-01 | -0.65 | 7.87E-01 | 8.75E-01 |
| L frontal pole | 5.04E-01 | 0.29 | 9.53E-01 | 1.00E+00 | -0.46 | 8.88E-01 | 9.86E-01 | -1.17 | 4.64E-01 | 5.70E-01 |
| R inferior parietal cortex | 5.55E-01 | 0.65 | 7.90E-01 | 1.00E+00 | -0.02 | 1.00E+00 | 1.00E+00 | -1.01 | 5.62E-01 | 6.56E-01 |
| L rostral anterior cingulate | 6.06E-01 | -0.80 | 7.00E-01 | 1.00E+00 | -1.00 | 5.70E-01 | 8.59E-01 | -0.37 | 9.24E-01 | 9.66E-01 |
| R transverse temporal gyrus | 8.27E-01 | 0.56 | 8.35E-01 | 1.00E+00 | 0.60 | 8.18E-01 | 9.23E-01 | 0.09 | 9.96E-01 | 9.99E-01 |
| R banks of STS | 9.27E-01 | 0.16 | 9.86E-01 | 1.00E+00 | 0.34 | 9.37E-01 | 9.94E-01 | 0.30 | 9.50E-01 | 9.78E-01 |
| R rostral anterior cingulate | 9.41E-01 | 0.32 | 9.44E-01 | 1.00E+00 | 0.34 | 9.37E-01 | 9.94E-01 | 0.05 | 9.99E-01 | 9.99E-01 |

**Table S16a.** Comparison of Cortical Thickness Differences among Subjects with A-D deletions, A-B Deletions and Controls. The following linear model was used for the group comparison: CT ~ Group + Site + Sex + Age + Age^2^. Df=203 for all comparisons.

# **Table S16b.** Comparison of Surface Area Differences between Subject with A-D deletions, A-B Deletions and Controls.

| Region | p-value  3 groups | t-value  AD>AB | p-value  AD>AB | p-FDR  AD>AB | t-value  CL>AB | p-value  CL>AB | p-FDR  CL>AB | t-value  CL>AD | p-value  CL>AD | p-FDR  CL>AD |
| --- | --- | --- | --- | --- | --- | --- | --- | --- | --- | --- |
| L cuneus | 1.39E-26 | -1.62 | 2.33E-01 | 3.88E-01 | 6.80 | 6.97E-10 | 4.88E-08 | 12.57 | 0.00E+00 | 0.00E+00 |
| R lingual gyrus | 5.73E-24 | -2.77 | 1.64E-02 | 5.98E-02 | 5.23 | 1.21E-06 | 2.82E-05 | 11.86 | 0.00E+00 | 0.00E+00 |
| R cuneus | 6.79E-22 | -3.37 | 2.53E-03 | 1.18E-02 | 4.21 | 1.18E-04 | 8.27E-04 | 11.18 | 0.00E+00 | 0.00E+00 |
| R pericalcarine cortex | 1.01E-17 | -0.04 | 9.99E-01 | 9.99E-01 | 6.20 | 1.02E-08 | 3.58E-07 | 9.38 | 0.00E+00 | 0.00E+00 |
| L lingual gyrus | 3.54E-17 | -1.47 | 3.03E-01 | 4.72E-01 | 4.91 | 5.44E-06 | 8.11E-05 | 9.50 | 1.11E-16 | 1.30E-15 |
| L pericalcarine cortex | 1.50E-16 | -1.53 | 2.73E-01 | 4.35E-01 | 4.71 | 1.41E-05 | 1.24E-04 | 9.29 | 1.11E-16 | 1.30E-15 |
| L precuneus | 6.00E-16 | -1.24 | 4.23E-01 | 6.30E-01 | 4.82 | 8.00E-06 | 9.33E-05 | 9.05 | 7.77E-16 | 7.77E-15 |
| R precuneus | 6.48E-13 | -2.52 | 3.17E-02 | 1.01E-01 | 2.92 | 1.04E-02 | 4.04E-02 | 8.04 | 1.21E-13 | 1.06E-12 |
| R rostral middle frontal gyrus | 2.02E-12 | -3.37 | 2.51E-03 | 1.18E-02 | 1.93 | 1.30E-01 | 3.02E-01 | 7.76 | 1.55E-12 | 1.20E-11 |
| L lateral occipital cortex | 4.73E-12 | -4.22 | 1.07E-04 | 1.72E-03 | 0.87 | 6.57E-01 | 9.79E-01 | 7.38 | 8.50E-12 | 5.41E-11 |
| L superior parietal cortex | 7.76E-12 | -0.62 | 8.06E-01 | 9.95E-01 | 4.37 | 5.74E-05 | 4.47E-04 | 7.47 | 4.14E-12 | 2.90E-11 |
| R hemisphere | 2.20E-11 | -3.72 | 7.25E-04 | 4.70E-03 | 1.25 | 4.17E-01 | 7.26E-01 | 7.25 | 2.43E-11 | 1.42E-10 |
| L hemisphere | 3.76E-11 | -3.69 | 8.06E-04 | 4.70E-03 | 1.21 | 4.40E-01 | 7.30E-01 | 7.15 | 5.05E-11 | 2.72E-10 |
| L rostral middle frontal gyrus | 6.06E-11 | -4.18 | 1.23E-04 | 1.72E-03 | 0.57 | 8.31E-01 | 1.00E+00 | 6.89 | 1.76E-10 | 8.78E-10 |
| R superior parietal cortex | 8.05E-10 | 0.58 | 8.26E-01 | 9.95E-01 | 4.78 | 9.67E-06 | 9.67E-05 | 6.35 | 4.35E-09 | 1.90E-08 |
| L fusiform gyrus | 1.25E-09 | -2.21 | 6.90E-02 | 1.61E-01 | 2.35 | 4.93E-02 | 1.38E-01 | 6.73 | 4.45E-10 | 2.08E-09 |
| L pars triangularis | 8.44E-09 | -4.31 | 7.59E-05 | 1.72E-03 | -0.31 | 9.47E-01 | 1.00E+00 | 5.74 | 1.20E-07 | 4.19E-07 |
| R caudal anterior cingulate | 3.78E-08 | -0.26 | 9.62E-01 | 9.99E-01 | 3.68 | 8.26E-04 | 4.82E-03 | 5.92 | 4.26E-08 | 1.66E-07 |
| L caudal anterior cingulate | 4.74E-08 | -2.49 | 3.45E-02 | 1.02E-01 | 1.60 | 2.44E-01 | 4.74E-01 | 6.00 | 2.69E-08 | 1.11E-07 |
| R inferior temporal gyrus | 1.32E-07 | -1.06 | 5.37E-01 | 7.36E-01 | 2.86 | 1.25E-02 | 4.61E-02 | 5.82 | 7.58E-08 | 2.79E-07 |
| R fusiform gyrus | 2.81E-07 | -0.60 | 8.18E-01 | 9.95E-01 | 3.16 | 5.06E-03 | 2.21E-02 | 5.61 | 1.75E-07 | 5.82E-07 |
| R lateral occipital cortex | 4.62E-07 | -2.06 | 9.84E-02 | 1.97E-01 | 1.74 | 1.90E-01 | 3.92E-01 | 5.58 | 2.54E-07 | 8.09E-07 |
| R paracentral lobule | 1.05E-06 | -4.78 | 9.53E-06 | 6.67E-04 | -2.04 | 1.02E-01 | 2.54E-01 | 3.82 | 5.00E-04 | 1.17E-03 |
| L rostral anterior cingulate | 6.72E-06 | -3.22 | 4.04E-03 | 1.77E-02 | -0.02 | 1.00E+00 | 1.00E+00 | 4.61 | 2.02E-05 | 5.90E-05 |
| R precentral gyrus | 1.24E-05 | -3.89 | 3.78E-04 | 3.31E-03 | -4.88 | 5.79E-06 | 8.11E-05 | -1.74 | 1.90E-01 | 2.29E-01 |
| L temporal pole | 1.28E-05 | -0.47 | 8.82E-01 | 9.99E-01 | 2.73 | 1.79E-02 | 6.28E-02 | 4.79 | 9.60E-06 | 2.92E-05 |
| L paracentral lobule | 2.96E-05 | -4.61 | 2.02E-05 | 7.09E-04 | -3.02 | 7.64E-03 | 3.14E-02 | 2.10 | 8.94E-02 | 1.25E-01 |
| L postcentral gyrus | 3.61E-05 | -2.46 | 3.79E-02 | 1.02E-01 | 0.62 | 8.08E-01 | 1.00E+00 | 4.47 | 3.67E-05 | 1.03E-04 |
| R pars triangularis | 4.02E-05 | -3.38 | 2.38E-03 | 1.18E-02 | -0.60 | 8.15E-01 | 1.00E+00 | 3.97 | 2.91E-04 | 7.02E-04 |
| L middle temporal gyrus | 4.25E-05 | -2.79 | 1.53E-02 | 5.96E-02 | 0.18 | 9.81E-01 | 1.00E+00 | 4.30 | 7.63E-05 | 1.98E-04 |
| L inferior temporal gyrus | 5.03E-05 | -0.38 | 9.23E-01 | 9.99E-01 | 2.61 | 2.55E-02 | 7.78E-02 | 4.47 | 3.84E-05 | 1.04E-04 |
| R posterior cingulate | 7.88E-05 | -4.05 | 2.03E-04 | 2.11E-03 | -1.95 | 1.24E-01 | 3.00E-01 | 2.91 | 1.07E-02 | 1.79E-02 |
| R middle temporal gyrus | 1.41E-04 | -2.46 | 3.72E-02 | 1.02E-01 | 0.35 | 9.33E-01 | 1.00E+00 | 4.08 | 1.82E-04 | 4.55E-04 |
| L precentral gyrus | 1.44E-04 | -1.94 | 1.26E-01 | 2.38E-01 | -3.94 | 3.20E-04 | 2.03E-03 | -3.13 | 5.47E-03 | 1.09E-02 |
| L pars opercularis | 2.92E-04 | -4.04 | 2.11E-04 | 2.11E-03 | -2.61 | 2.50E-02 | 7.78E-02 | 1.89 | 1.39E-01 | 1.74E-01 |
| L posterior cingulate | 4.32E-04 | -3.79 | 5.68E-04 | 4.42E-03 | -2.09 | 9.16E-02 | 2.44E-01 | 2.33 | 5.28E-02 | 7.70E-02 |
| R insula | 5.42E-04 | -1.71 | 2.00E-01 | 3.50E-01 | -3.59 | 1.18E-03 | 6.35E-03 | -2.94 | 9.95E-03 | 1.70E-02 |
| L superior frontal gyrus | 5.83E-04 | -2.42 | 4.15E-02 | 1.08E-01 | 0.11 | 9.93E-01 | 1.00E+00 | 3.65 | 9.11E-04 | 1.99E-03 |
| L frontal pole | 6.01E-04 | -2.87 | 1.19E-02 | 4.92E-02 | -0.51 | 8.63E-01 | 1.00E+00 | 3.37 | 2.45E-03 | 5.20E-03 |
| L insula | 7.71E-04 | -1.53 | 2.72E-01 | 4.35E-01 | -3.44 | 1.97E-03 | 9.87E-03 | -2.96 | 9.28E-03 | 1.67E-02 |
| R postcentral gyrus | 8.10E-04 | -0.46 | 8.89E-01 | 9.99E-01 | 2.08 | 9.42E-02 | 2.44E-01 | 3.79 | 5.79E-04 | 1.31E-03 |
| R superior frontal gyrus | 1.22E-03 | -3.69 | 7.97E-04 | 4.70E-03 | -2.54 | 3.02E-02 | 8.81E-02 | 1.50 | 2.88E-01 | 3.31E-01 |
| R temporal pole | 2.79E-03 | 2.12 | 8.64E-02 | 1.78E-01 | 3.39 | 2.31E-03 | 1.08E-02 | 2.05 | 9.95E-02 | 1.35E-01 |
| R banks of STS | 3.38E-03 | -2.15 | 7.98E-02 | 1.70E-01 | 0.04 | 9.99E-01 | 1.00E+00 | 3.16 | 5.00E-03 | 1.03E-02 |
| R STG | 5.78E-03 | -2.15 | 7.97E-02 | 1.70E-01 | -0.10 | 9.94E-01 | 1.00E+00 | 2.95 | 9.65E-03 | 1.69E-02 |
| R pars orbitalis | 6.40E-03 | -1.88 | 1.42E-01 | 2.61E-01 | 0.21 | 9.75E-01 | 1.00E+00 | 3.03 | 7.33E-03 | 1.43E-02 |
| R supramarginal gyrus | 8.25E-03 | -1.77 | 1.79E-01 | 3.21E-01 | 0.29 | 9.54E-01 | 1.00E+00 | 2.98 | 8.63E-03 | 1.59E-02 |
| L medial orbitofrontal cortex | 1.13E-02 | -2.71 | 1.89E-02 | 6.31E-02 | -1.24 | 4.25E-01 | 7.26E-01 | 2.05 | 1.00E-01 | 1.35E-01 |
| R rostral anterior cingulate | 1.20E-02 | -0.83 | 6.78E-01 | 8.87E-01 | 1.20 | 4.48E-01 | 7.30E-01 | 3.01 | 7.99E-03 | 1.51E-02 |
| L STG | 1.36E-02 | -1.99 | 1.14E-01 | 2.21E-01 | -0.13 | 9.90E-01 | 1.00E+00 | 2.67 | 2.15E-02 | 3.34E-02 |
| L caudal middle frontal gyrus | 1.63E-02 | -2.75 | 1.71E-02 | 5.98E-02 | -2.70 | 1.97E-02 | 6.57E-02 | -0.10 | 9.95E-01 | 9.99E-01 |
| L pars orbitalis | 1.64E-02 | -0.10 | 9.95E-01 | 9.99E-01 | 1.77 | 1.77E-01 | 3.88E-01 | 2.80 | 1.47E-02 | 2.34E-02 |
| L entorhinal cortex | 1.95E-02 | -0.56 | 8.38E-01 | 9.95E-01 | 1.34 | 3.69E-01 | 6.63E-01 | 2.82 | 1.39E-02 | 2.26E-02 |
| L banks of STS | 2.63E-02 | -2.47 | 3.67E-02 | 1.02E-01 | -1.17 | 4.64E-01 | 7.38E-01 | 1.80 | 1.69E-01 | 2.08E-01 |
| R medial orbitofrontal cortex | 2.67E-02 | -2.38 | 4.60E-02 | 1.15E-01 | -1.00 | 5.71E-01 | 8.89E-01 | 1.93 | 1.30E-01 | 1.67E-01 |
| R inferior parietal cortex | 2.74E-02 | -2.15 | 8.04E-02 | 1.70E-01 | -0.61 | 8.14E-01 | 1.00E+00 | 2.18 | 7.38E-02 | 1.05E-01 |
| R entorhinal cortex | 2.94E-02 | -0.22 | 9.73E-01 | 9.99E-01 | 1.53 | 2.72E-01 | 5.02E-01 | 2.62 | 2.43E-02 | 3.70E-02 |
| L transverse temporal gyrus | 5.98E-02 | -0.82 | 6.84E-01 | 8.87E-01 | 0.80 | 7.01E-01 | 1.00E+00 | 2.38 | 4.55E-02 | 6.78E-02 |
| L isthmus cingulate | 8.47E-02 | -1.64 | 2.28E-01 | 3.88E-01 | -0.29 | 9.54E-01 | 1.00E+00 | 1.92 | 1.31E-01 | 1.67E-01 |
| R caudal middle frontal gyrus | 8.88E-02 | -2.21 | 6.91E-02 | 1.61E-01 | -1.73 | 1.91E-01 | 3.92E-01 | 0.58 | 8.26E-01 | 8.51E-01 |
| R lateral orbitofrontal cortex | 9.48E-02 | 0.20 | 9.78E-01 | 9.99E-01 | 1.54 | 2.69E-01 | 5.02E-01 | 2.03 | 1.05E-01 | 1.38E-01 |
| R pars opercularis | 1.66E-01 | -1.18 | 4.59E-01 | 6.42E-01 | -1.86 | 1.48E-01 | 3.35E-01 | -1.10 | 5.10E-01 | 5.67E-01 |
| R transverse temporal gyrus | 2.14E-01 | -1.21 | 4.43E-01 | 6.39E-01 | -0.11 | 9.93E-01 | 1.00E+00 | 1.57 | 2.55E-01 | 3.02E-01 |
| R frontal pole | 2.40E-01 | -1.29 | 3.98E-01 | 6.06E-01 | -1.70 | 2.05E-01 | 4.09E-01 | -0.70 | 7.63E-01 | 8.09E-01 |
| L lateral orbitofrontal cortex | 3.02E-01 | -0.13 | 9.90E-01 | 9.99E-01 | 0.88 | 6.47E-01 | 9.79E-01 | 1.52 | 2.78E-01 | 3.24E-01 |
| L inferior parietal cortex | 3.05E-01 | -1.00 | 5.75E-01 | 7.74E-01 | -0.01 | 1.00E+00 | 1.00E+00 | 1.42 | 3.28E-01 | 3.71E-01 |
| R isthmus cingulate | 4.65E-01 | -1.20 | 4.48E-01 | 6.39E-01 | -0.73 | 7.43E-01 | 1.00E+00 | 0.64 | 7.97E-01 | 8.33E-01 |
| R parahippocampal gyrus | 6.80E-01 | -0.42 | 9.05E-01 | 9.99E-01 | 0.17 | 9.85E-01 | 1.00E+00 | 0.86 | 6.62E-01 | 7.24E-01 |
| L parahippocampal gyrus | 7.07E-01 | 0.59 | 8.23E-01 | 9.95E-01 | 0.08 | 9.97E-01 | 1.00E+00 | -0.73 | 7.41E-01 | 7.98E-01 |
| L supramarginal gyrus | 8.88E-01 | 0.45 | 8.91E-01 | 9.99E-01 | 0.46 | 8.87E-01 | 1.00E+00 | 0.04 | 9.99E-01 | 9.99E-01 |

**Table S16b.** Comparison of Surface Area Differences between Subject with A-D deletions, A-B Deletions and Controls. The following linear model was used for the group comparison: SA ~ Group + Site + Sex + Age + ICV. Df=203 for all comparisons.

**Table S17a. Effects of Psychotropic Medications on CT**.

| **Region** | **T.TA** | **P.TA** | **P.TA FDR** | **T.AA** | **P.AA** | **P.AA FDR** | **T.AC** | **P.AC** | **P.AC FDR** | **T.AD** | **P.AD** | **P.AD FDR** |
| --- | --- | --- | --- | --- | --- | --- | --- | --- | --- | --- | --- | --- |
| **L banks of STS** | -0.24 | 0.81 | 0.90 | -1.79 | 0.07 | 0.37 | -0.86 | 0.39 | 0.55 | -0.79 | 0.43 | 0.85 |
| **L caudal anterior cingulate** | 0.05 | 0.96 | 0.99 | 0.12 | 0.91 | 0.96 | -0.60 | 0.55 | 0.68 | 0.33 | 0.74 | 0.89 |
| **L caudal middle frontal gyrus** | -0.60 | 0.55 | 0.76 | -0.12 | 0.90 | 0.96 | -2.03 | 0.04 | 0.26 | -0.74 | 0.46 | 0.85 |
| **L cuneus** | 0.72 | 0.47 | 0.72 | 1.10 | 0.27 | 0.55 | 0.54 | 0.59 | 0.70 | -1.03 | 0.31 | 0.79 |
| **L entorhinal cortex** | -0.61 | 0.54 | 0.76 | -1.10 | 0.27 | 0.55 | -1.95 | 0.05 | 0.26 | -1.11 | 0.27 | 0.79 |
| **L fusiform gyrus** | -1.88 | 0.06 | 0.28 | -1.10 | 0.27 | 0.55 | -2.07 | 0.04 | 0.26 | -1.49 | 0.14 | 0.79 |
| **L inferior parietal cortex** | -1.28 | 0.20 | 0.44 | 0.13 | 0.89 | 0.96 | -0.69 | 0.49 | 0.63 | -1.10 | 0.27 | 0.79 |
| **L inferior temporal gyrus** | -1.94 | 0.05 | 0.28 | -2.63 | 0.01 | 0.19 | -1.66 | 0.10 | 0.31 | -0.56 | 0.58 | 0.86 |
| **L isthmus cingulate** | 0.12 | 0.91 | 0.98 | -0.40 | 0.69 | 0.89 | -0.74 | 0.46 | 0.60 | -1.01 | 0.31 | 0.79 |
| **L lateral occipital cortex** | -0.02 | 0.99 | 0.99 | 0.91 | 0.36 | 0.65 | 0.81 | 0.42 | 0.55 | -0.75 | 0.45 | 0.85 |
| **L lateral orbitofrontal cortex** | -2.16 | 0.03 | 0.28 | -1.33 | 0.18 | 0.47 | -1.94 | 0.05 | 0.26 | -0.60 | 0.55 | 0.86 |
| **L lingual gyrus** | 0.65 | 0.52 | 0.75 | -0.06 | 0.95 | 0.98 | 0.28 | 0.78 | 0.85 | -0.90 | 0.37 | 0.80 |
| **L medial orbitofrontal cortex** | -2.01 | 0.05 | 0.28 | -1.55 | 0.12 | 0.40 | -1.49 | 0.14 | 0.34 | -1.42 | 0.16 | 0.79 |
| **L middle temporal gyrus** | -1.91 | 0.06 | 0.28 | -2.26 | 0.02 | 0.25 | -1.64 | 0.10 | 0.31 | -0.86 | 0.39 | 0.83 |
| **L parahippocampal gyrus** | -0.01 | 0.99 | 0.99 | 0.26 | 0.79 | 0.94 | -0.96 | 0.34 | 0.52 | -0.57 | 0.57 | 0.86 |
| **L paracentral lobule** | 0.29 | 0.77 | 0.88 | -0.49 | 0.62 | 0.86 | -0.81 | 0.42 | 0.55 | -0.49 | 0.62 | 0.86 |
| **L pars opercularis** | -1.79 | 0.07 | 0.28 | -2.53 | 0.01 | 0.19 | -2.39 | 0.02 | 0.24 | 0.08 | 0.94 | 0.98 |
| **L pars orbitalis** | -1.50 | 0.14 | 0.38 | -1.68 | 0.09 | 0.39 | -1.80 | 0.07 | 0.28 | -0.46 | 0.65 | 0.86 |
| **L pars triangularis** | -1.32 | 0.19 | 0.44 | -0.66 | 0.51 | 0.76 | -0.48 | 0.63 | 0.72 | 0.20 | 0.84 | 0.94 |
| **L pericalcarine cortex** | 2.26 | 0.02 | 0.28 | 0.62 | 0.54 | 0.76 | 1.09 | 0.28 | 0.46 | -0.55 | 0.58 | 0.86 |
| **L postcentral gyrus** | -0.68 | 0.50 | 0.74 | -1.07 | 0.28 | 0.55 | -1.29 | 0.20 | 0.43 | -1.49 | 0.14 | 0.79 |
| **L posterior cingulate** | -2.06 | 0.04 | 0.28 | 0.46 | 0.65 | 0.87 | -1.33 | 0.19 | 0.42 | 0.03 | 0.97 | 0.98 |
| **L precentral gyrus** | -0.78 | 0.44 | 0.70 | -1.73 | 0.08 | 0.39 | -1.73 | 0.09 | 0.30 | -1.46 | 0.15 | 0.79 |
| **L precuneus** | -1.20 | 0.23 | 0.46 | -0.58 | 0.56 | 0.79 | -0.83 | 0.41 | 0.55 | -0.45 | 0.65 | 0.86 |
| **L rostral anterior cingulate** | -0.50 | 0.62 | 0.80 | -0.98 | 0.33 | 0.61 | -1.19 | 0.24 | 0.44 | -0.47 | 0.64 | 0.86 |
| **L rostral middle frontal gyrus** | -1.29 | 0.20 | 0.44 | -0.64 | 0.53 | 0.76 | -1.75 | 0.08 | 0.30 | -0.17 | 0.86 | 0.94 |
| **L superior frontal gyrus** | -1.28 | 0.20 | 0.44 | -1.95 | 0.05 | 0.36 | -2.61 | 0.01 | 0.23 | -1.09 | 0.28 | 0.79 |
| **L superior parietal cortex** | -0.34 | 0.73 | 0.87 | 0.69 | 0.49 | 0.76 | -0.59 | 0.56 | 0.68 | -0.70 | 0.48 | 0.86 |
| **L STG** | -0.85 | 0.40 | 0.66 | -1.83 | 0.07 | 0.37 | -1.83 | 0.07 | 0.28 | -1.00 | 0.32 | 0.79 |
| **L supramarginal gyrus** | -1.23 | 0.22 | 0.45 | -0.38 | 0.71 | 0.90 | -1.42 | 0.16 | 0.36 | -0.67 | 0.50 | 0.86 |
| **L frontal pole** | 0.09 | 0.93 | 0.98 | -0.68 | 0.50 | 0.76 | -0.05 | 0.96 | 0.97 | -0.82 | 0.41 | 0.85 |
| **L temporal pole** | -0.76 | 0.45 | 0.70 | -1.96 | 0.05 | 0.36 | -1.12 | 0.26 | 0.46 | -0.11 | 0.91 | 0.98 |
| **L transverse temporal gyrus** | -2.18 | 0.03 | 0.28 | -0.99 | 0.33 | 0.61 | -0.50 | 0.61 | 0.72 | 0.94 | 0.35 | 0.80 |
| **L insula** | -1.73 | 0.08 | 0.28 | -1.80 | 0.07 | 0.37 | -2.06 | 0.04 | 0.26 | -1.14 | 0.25 | 0.79 |
| **R banks of STS** | -1.86 | 0.06 | 0.28 | 0.15 | 0.88 | 0.96 | -1.13 | 0.26 | 0.46 | 0.36 | 0.72 | 0.89 |
| **R caudal anterior cingulate** | -0.35 | 0.73 | 0.87 | -0.68 | 0.49 | 0.76 | -1.49 | 0.14 | 0.34 | -1.44 | 0.15 | 0.79 |
| **R caudal middle frontal gyrus** | -0.28 | 0.78 | 0.88 | -1.16 | 0.24 | 0.55 | -0.42 | 0.67 | 0.76 | -1.24 | 0.22 | 0.79 |
| **R cuneus** | -0.05 | 0.96 | 0.99 | -0.08 | 0.94 | 0.98 | -0.54 | 0.59 | 0.70 | -1.55 | 0.12 | 0.79 |
| **R entorhinal cortex** | -1.82 | 0.07 | 0.28 | -1.08 | 0.28 | 0.55 | -2.36 | 0.02 | 0.24 | -0.26 | 0.79 | 0.91 |
| **R fusiform gyrus** | -2.14 | 0.03 | 0.28 | -1.80 | 0.07 | 0.37 | -2.75 | 0.01 | 0.23 | -0.57 | 0.57 | 0.86 |
| **R inferior parietal cortex** | -1.51 | 0.13 | 0.38 | 0.14 | 0.89 | 0.96 | -0.87 | 0.39 | 0.55 | -1.83 | 0.07 | 0.79 |
| **R inferior temporal gyrus** | -1.92 | 0.06 | 0.28 | -3.53 | 0.00 | 0.03 | -2.32 | 0.02 | 0.24 | -0.98 | 0.33 | 0.79 |
| **R isthmus cingulate** | -1.29 | 0.20 | 0.44 | -0.20 | 0.84 | 0.96 | -0.25 | 0.81 | 0.87 | -0.56 | 0.58 | 0.86 |
| **R lateral occipital cortex** | -0.51 | 0.61 | 0.80 | 0.02 | 0.99 | 0.99 | -0.17 | 0.86 | 0.90 | -0.74 | 0.46 | 0.85 |
| **R lateral orbitofrontal cortex** | -1.02 | 0.31 | 0.55 | -0.77 | 0.44 | 0.76 | -0.14 | 0.89 | 0.92 | -1.00 | 0.32 | 0.79 |
| **R lingual gyrus** | 0.49 | 0.63 | 0.80 | 0.76 | 0.45 | 0.76 | -0.02 | 0.98 | 0.98 | -1.67 | 0.10 | 0.79 |
| **R medial orbitofrontal cortex** | -1.07 | 0.28 | 0.53 | -1.29 | 0.20 | 0.47 | -1.24 | 0.22 | 0.44 | -2.18 | 0.03 | 0.79 |
| **R middle temporal gyrus** | -1.64 | 0.10 | 0.31 | -1.43 | 0.15 | 0.43 | -1.21 | 0.23 | 0.44 | -1.20 | 0.23 | 0.79 |
| **R parahippocampal gyrus** | 0.59 | 0.55 | 0.76 | 0.67 | 0.50 | 0.76 | -1.59 | 0.11 | 0.32 | -0.33 | 0.74 | 0.89 |
| **R paracentral lobule** | -0.34 | 0.74 | 0.87 | -0.31 | 0.76 | 0.94 | -1.20 | 0.23 | 0.44 | 0.25 | 0.80 | 0.91 |
| **R pars opercularis** | -1.39 | 0.17 | 0.43 | -1.32 | 0.19 | 0.47 | -1.08 | 0.28 | 0.46 | -1.08 | 0.28 | 0.79 |
| **R pars orbitalis** | -1.78 | 0.07 | 0.28 | -2.25 | 0.02 | 0.25 | -1.45 | 0.15 | 0.35 | -0.29 | 0.77 | 0.91 |
| **R pars triangularis** | -1.77 | 0.08 | 0.28 | -1.12 | 0.26 | 0.55 | -1.81 | 0.07 | 0.28 | -0.25 | 0.81 | 0.91 |
| **R pericalcarine cortex** | 0.09 | 0.93 | 0.98 | 0.29 | 0.77 | 0.94 | -0.86 | 0.39 | 0.55 | -1.52 | 0.13 | 0.79 |
| **R postcentral gyrus** | -1.87 | 0.06 | 0.28 | -1.44 | 0.15 | 0.43 | -0.30 | 0.76 | 0.85 | -1.05 | 0.30 | 0.79 |
| **R posterior cingulate** | -1.78 | 0.08 | 0.28 | -0.24 | 0.81 | 0.94 | -1.58 | 0.11 | 0.32 | -0.02 | 0.98 | 0.98 |
| **R precentral gyrus** | -0.31 | 0.75 | 0.88 | -1.57 | 0.12 | 0.40 | 0.20 | 0.85 | 0.90 | -0.58 | 0.56 | 0.86 |
| **R precuneus** | -0.88 | 0.38 | 0.65 | 0.42 | 0.68 | 0.89 | -0.93 | 0.35 | 0.53 | -1.03 | 0.31 | 0.79 |
| **R rostral anterior cingulate** | -1.08 | 0.28 | 0.53 | 0.03 | 0.98 | 0.99 | -1.55 | 0.12 | 0.33 | -1.02 | 0.31 | 0.79 |
| **R rostral middle frontal gyrus** | -1.77 | 0.08 | 0.28 | -1.60 | 0.11 | 0.40 | -1.64 | 0.10 | 0.31 | -0.40 | 0.69 | 0.88 |
| **R superior frontal gyrus** | -1.69 | 0.09 | 0.29 | -2.70 | 0.01 | 0.19 | -2.59 | 0.01 | 0.23 | -2.26 | 0.02 | 0.79 |
| **R superior parietal cortex** | -0.79 | 0.43 | 0.70 | 0.26 | 0.80 | 0.94 | -1.24 | 0.21 | 0.44 | -0.47 | 0.64 | 0.86 |
| **R STG** | -0.90 | 0.37 | 0.65 | -2.49 | 0.01 | 0.19 | -2.06 | 0.04 | 0.26 | 0.07 | 0.95 | 0.98 |
| **R supramarginal gyrus** | -1.85 | 0.06 | 0.28 | -1.52 | 0.13 | 0.40 | -1.05 | 0.29 | 0.47 | -0.51 | 0.61 | 0.86 |
| **R frontal pole** | -0.55 | 0.58 | 0.79 | -0.63 | 0.53 | 0.76 | -0.92 | 0.36 | 0.54 | 0.06 | 0.96 | 0.98 |
| **R temporal pole** | -0.44 | 0.66 | 0.82 | -1.63 | 0.10 | 0.40 | -2.12 | 0.03 | 0.26 | -0.41 | 0.68 | 0.88 |
| **R transverse temporal gyrus** | -1.27 | 0.21 | 0.44 | -1.99 | 0.05 | 0.36 | -1.21 | 0.23 | 0.44 | 1.97 | 0.05 | 0.79 |
| **R insula** | -1.07 | 0.29 | 0.53 | -1.51 | 0.13 | 0.40 | -1.08 | 0.28 | 0.46 | -1.18 | 0.24 | 0.79 |
| **L hemisphere** | -1.48 | 0.14 | 0.38 | -1.41 | 0.16 | 0.43 | -1.98 | 0.05 | 0.26 | -0.92 | 0.36 | 0.80 |
| **R hemisphere** | -1.74 | 0.08 | 0.28 | -1.70 | 0.09 | 0.39 | -1.91 | 0.06 | 0.27 | -1.21 | 0.23 | 0.79 |

T-values and p-values for CT comparison of 22q11DS patients on vs. not on each category of psychotropic medications. No significant effects were detected between psychotropic medications at the time of MRI and CT measure in any cortical regions.

**T.TA**: t-values of typical antipsychotic effects; **P.TA**: p-values of typical antipsychotic effects; **P.TA FDR**: FDR-corrected p-values of typical antipsychotic effects. **T.AA**: t-values of atypical antipsychotic effects; **P.AA**: p-values of atypical antipsychotic effects; **P.AA FDR**: FDR-corrected p-values of atypical antipsychotic effects. **T.AC**: t-values of anticonvulsant effects; **P.AC**: p-values of anticonvulsant effects; **P.AC FDR**: FDR-corrected p-values of anticonvulsant effects. **T.AD**: t-values of antidepressant effects; **P.AD**: p-values of antidepressant effects; **P.AD FDR**: FDR-corrected p-values of antidepressant effects.

**Table S17b. Effects of Psychotropic Medications on SA.**

| **Region** | **T.TA** | **P.TA** | **P.TA FDR** | **T.AA** | **P.AA** | **P.AA FDR** | **T.AC** | **P.AC** | **P.AC FDR** | **T.AD** | **P.AD** | **P.AD FDR** |
| --- | --- | --- | --- | --- | --- | --- | --- | --- | --- | --- | --- | --- |
| **L banks of STS** | 1.03 | 0.30 | 0.78 | -0.67 | 0.50 | 0.99 | 0.29 | 0.77 | 0.99 | -0.70 | 0.48 | 0.99 |
| **L caudal anterior cingulate** | 0.67 | 0.50 | 0.88 | -1.28 | 0.20 | 0.99 | -0.51 | 0.61 | 0.99 | 0.16 | 0.87 | 0.99 |
| **L caudal middle frontal gyrus** | 1.54 | 0.13 | 0.78 | -0.91 | 0.36 | 0.99 | -0.86 | 0.39 | 0.96 | -1.07 | 0.29 | 0.99 |
| **L cuneus** | -0.20 | 0.85 | 1.00 | 0.32 | 0.75 | 0.99 | 0.29 | 0.77 | 0.99 | 0.25 | 0.81 | 0.99 |
| **L entorhinal cortex** | -0.71 | 0.48 | 0.88 | -0.62 | 0.53 | 0.99 | -1.10 | 0.27 | 0.79 | 0.81 | 0.42 | 0.99 |
| **L frontal pole** | 0.57 | 0.57 | 0.88 | -1.45 | 0.15 | 0.99 | -1.43 | 0.15 | 0.76 | -1.01 | 0.32 | 0.99 |
| **L fusiform gyrus** | 0.36 | 0.72 | 0.93 | -0.27 | 0.79 | 0.99 | 0.04 | 0.97 | 0.99 | 1.60 | 0.11 | 0.99 |
| **L hemisphere** | 0.96 | 0.34 | 0.78 | -0.18 | 0.86 | 0.99 | -0.79 | 0.43 | 0.99 | 0.41 | 0.68 | 0.99 |
| **L inferior parietal cortex** | -1.26 | 0.21 | 0.78 | -1.15 | 0.25 | 0.99 | -0.74 | 0.46 | 0.99 | 0.69 | 0.49 | 0.99 |
| **L inferior temporal gyrus** | 0.31 | 0.76 | 0.95 | -2.12 | 0.04 | 0.99 | -1.47 | 0.14 | 0.76 | 0.80 | 0.43 | 0.99 |
| **L insula** | 1.00 | 0.32 | 0.78 | 1.27 | 0.21 | 0.99 | -0.02 | 0.99 | 0.99 | 0.76 | 0.45 | 0.99 |
| **L isthmus cingulate** | 1.51 | 0.13 | 0.78 | 0.04 | 0.97 | 0.99 | -0.08 | 0.93 | 0.99 | 1.15 | 0.25 | 0.99 |
| **L lateral occipital cortex** | 0.16 | 0.87 | 1.00 | 0.25 | 0.80 | 0.99 | -0.58 | 0.56 | 0.99 | -1.15 | 0.25 | 0.99 |
| **L lateral orbitofrontal cortex** | -0.53 | 0.60 | 0.89 | 0.22 | 0.82 | 0.99 | -1.41 | 0.16 | 0.76 | 1.47 | 0.14 | 0.99 |
| **L lingual gyrus** | 0.94 | 0.35 | 0.78 | 0.86 | 0.39 | 0.99 | 1.29 | 0.20 | 0.79 | -1.44 | 0.15 | 0.99 |
| **L medial orbitofrontal cortex** | 1.03 | 0.30 | 0.78 | 0.27 | 0.79 | 0.99 | -1.15 | 0.25 | 0.79 | 0.83 | 0.41 | 0.99 |
| **L middle temporal gyrus** | -0.03 | 0.98 | 1.00 | -0.83 | 0.41 | 0.99 | -0.70 | 0.48 | 0.99 | 0.40 | 0.69 | 0.99 |
| **L paracentral lobule** | 1.66 | 0.10 | 0.78 | 0.42 | 0.67 | 0.99 | -1.15 | 0.25 | 0.79 | -0.80 | 0.42 | 0.99 |
| **L parahippocampal gyrus** | 0.12 | 0.91 | 1.00 | -0.28 | 0.78 | 0.99 | -0.49 | 0.62 | 0.99 | 0.85 | 0.40 | 0.99 |
| **L pars opercularis** | -0.24 | 0.81 | 0.98 | 0.20 | 0.84 | 0.99 | -0.12 | 0.91 | 0.99 | 1.86 | 0.06 | 0.99 |
| **L pars orbitalis** | -0.03 | 0.97 | 1.00 | -0.53 | 0.60 | 0.99 | -0.18 | 0.86 | 0.99 | 1.01 | 0.31 | 0.99 |
| **L pars triangularis** | 0.45 | 0.66 | 0.91 | 0.51 | 0.61 | 0.99 | 0.02 | 0.99 | 0.99 | 1.13 | 0.26 | 0.99 |
| **L pericalcarine cortex** | 1.48 | 0.14 | 0.78 | 1.08 | 0.28 | 0.99 | 0.51 | 0.61 | 0.99 | -0.96 | 0.34 | 0.99 |
| **L postcentral gyrus** | 0.63 | 0.53 | 0.88 | 0.49 | 0.62 | 0.99 | -0.28 | 0.78 | 0.99 | -0.92 | 0.36 | 0.99 |
| **L posterior cingulate** | 0.97 | 0.33 | 0.78 | -0.07 | 0.94 | 0.99 | -0.53 | 0.59 | 0.99 | 1.05 | 0.29 | 0.99 |
| **L precentral gyrus** | 1.66 | 0.10 | 0.78 | 0.11 | 0.91 | 0.99 | -0.37 | 0.72 | 0.99 | -1.02 | 0.31 | 0.99 |
| **L precuneus** | -0.61 | 0.54 | 0.88 | 0.53 | 0.60 | 0.99 | -0.41 | 0.69 | 0.99 | 0.48 | 0.63 | 0.99 |
| **L rostral anterior cingulate** | 0.70 | 0.49 | 0.88 | -0.40 | 0.69 | 0.99 | -0.84 | 0.40 | 0.96 | 0.01 | 0.99 | 1.00 |
| **L rostral middle frontal gyrus** | 1.69 | 0.09 | 0.78 | -1.23 | 0.22 | 0.99 | -1.44 | 0.15 | 0.76 | 0.41 | 0.69 | 0.99 |
| **L STG** | 2.49 | 0.01 | 0.73 | 0.15 | 0.88 | 0.99 | -0.11 | 0.92 | 0.99 | 1.15 | 0.25 | 0.99 |
| **L superior frontal gyrus** | 0.42 | 0.67 | 0.91 | 0.33 | 0.74 | 0.99 | -1.06 | 0.29 | 0.79 | 0.57 | 0.57 | 0.99 |
| **L superior parietal cortex** | 1.27 | 0.20 | 0.78 | 0.24 | 0.81 | 0.99 | 0.21 | 0.83 | 0.99 | 0.11 | 0.91 | 0.99 |
| **L supramarginal gyrus** | 0.57 | 0.57 | 0.88 | 0.17 | 0.87 | 0.99 | -0.61 | 0.54 | 0.99 | 1.04 | 0.30 | 0.99 |
| **L temporal pole** | -0.55 | 0.58 | 0.89 | 1.65 | 0.10 | 0.99 | -0.93 | 0.35 | 0.92 | 1.36 | 0.17 | 0.99 |
| **L transverse temporal gyrus** | 0.43 | 0.67 | 0.91 | -0.98 | 0.33 | 0.99 | -1.77 | 0.08 | 0.76 | 0.68 | 0.50 | 0.99 |
| **R banks of STS** | 1.66 | 0.10 | 0.78 | -0.89 | 0.38 | 0.99 | 0.02 | 0.99 | 0.99 | 0.32 | 0.75 | 0.99 |
| **R caudal anterior cingulate** | 0.31 | 0.76 | 0.95 | 1.85 | 0.07 | 0.99 | -0.27 | 0.79 | 0.99 | -0.33 | 0.74 | 0.99 |
| **R caudal middle frontal gyrus** | -0.05 | 0.96 | 1.00 | -0.15 | 0.88 | 0.99 | -2.26 | 0.02 | 0.76 | -0.11 | 0.91 | 0.99 |
| **R cuneus** | 0.98 | 0.33 | 0.78 | 1.21 | 0.23 | 0.99 | 0.11 | 0.91 | 0.99 | -0.33 | 0.74 | 0.99 |
| **R entorhinal cortex** | 0.60 | 0.55 | 0.88 | -0.02 | 0.99 | 0.99 | -0.53 | 0.60 | 0.99 | -0.37 | 0.71 | 0.99 |
| **R frontal pole** | 0.69 | 0.49 | 0.88 | 0.50 | 0.62 | 0.99 | -1.56 | 0.12 | 0.76 | 0.22 | 0.83 | 0.99 |
| **R fusiform gyrus** | -0.76 | 0.45 | 0.88 | -1.02 | 0.31 | 0.99 | -1.51 | 0.13 | 0.76 | 0.45 | 0.65 | 0.99 |
| **R hemisphere** | 0.92 | 0.36 | 0.78 | -0.46 | 0.65 | 0.99 | -1.05 | 0.29 | 0.79 | 0.08 | 0.94 | 0.99 |
| **R inferior parietal cortex** | 0.00 | 1.00 | 1.00 | -1.50 | 0.14 | 0.99 | 0.06 | 0.95 | 0.99 | -0.46 | 0.64 | 0.99 |
| **R inferior temporal gyrus** | -0.37 | 0.71 | 0.93 | -1.68 | 0.09 | 0.99 | -2.12 | 0.04 | 0.76 | 0.39 | 0.70 | 0.99 |
| **R insula** | 1.36 | 0.18 | 0.78 | 0.57 | 0.57 | 0.99 | -0.37 | 0.71 | 0.99 | 0.62 | 0.53 | 0.99 |
| **R isthmus cingulate** | 0.14 | 0.89 | 1.00 | -0.07 | 0.94 | 0.99 | -0.52 | 0.60 | 0.99 | -0.64 | 0.52 | 0.99 |
| **R lateral occipital cortex** | 0.98 | 0.33 | 0.78 | -0.22 | 0.83 | 0.99 | 0.39 | 0.70 | 0.99 | 0.00 | 1.00 | 1.00 |
| **R lateral orbitofrontal cortex** | -1.01 | 0.32 | 0.78 | -0.76 | 0.45 | 0.99 | -2.48 | 0.01 | 0.76 | 0.22 | 0.83 | 0.99 |
| **R lingual gyrus** | 1.23 | 0.22 | 0.78 | 1.39 | 0.16 | 0.99 | 1.63 | 0.10 | 0.76 | 0.06 | 0.95 | 0.99 |
| **R medial orbitofrontal cortex** | -0.13 | 0.90 | 1.00 | 0.05 | 0.96 | 0.99 | -1.48 | 0.14 | 0.76 | 0.91 | 0.36 | 0.99 |
| **R middle temporal gyrus** | -0.06 | 0.96 | 1.00 | -1.52 | 0.13 | 0.99 | -1.25 | 0.21 | 0.79 | -0.20 | 0.84 | 0.99 |
| **R paracentral lobule** | 2.32 | 0.02 | 0.73 | -0.62 | 0.53 | 0.99 | 0.43 | 0.67 | 0.99 | -0.10 | 0.92 | 0.99 |
| **R parahippocampal gyrus** | 1.00 | 0.32 | 0.78 | -2.66 | 0.01 | 0.57 | -0.32 | 0.75 | 0.99 | -0.09 | 0.93 | 0.99 |
| **R pars opercularis** | 0.69 | 0.49 | 0.88 | 0.16 | 0.88 | 0.99 | -1.47 | 0.14 | 0.76 | -0.26 | 0.80 | 0.99 |
| **R pars orbitalis** | -1.69 | 0.09 | 0.78 | -0.90 | 0.37 | 0.99 | -1.36 | 0.17 | 0.76 | -0.14 | 0.89 | 0.99 |
| **R pars triangularis** | 1.37 | 0.17 | 0.78 | 0.82 | 0.41 | 0.99 | 0.06 | 0.95 | 0.99 | -0.24 | 0.81 | 0.99 |
| **R pericalcarine cortex** | 0.24 | 0.81 | 0.98 | 0.66 | 0.51 | 0.99 | 0.17 | 0.86 | 0.99 | -1.20 | 0.23 | 0.99 |
| **R postcentral gyrus** | 0.00 | 1.00 | 1.00 | -0.93 | 0.35 | 0.99 | -1.10 | 0.27 | 0.79 | -0.69 | 0.49 | 0.99 |
| **R posterior cingulate** | 0.98 | 0.33 | 0.78 | 1.41 | 0.16 | 0.99 | 0.23 | 0.82 | 0.99 | -0.05 | 0.96 | 0.99 |
| **R precentral gyrus** | 1.50 | 0.13 | 0.78 | -0.79 | 0.43 | 0.99 | -0.75 | 0.45 | 0.99 | -1.14 | 0.26 | 0.99 |
| **R precuneus** | -0.09 | 0.93 | 1.00 | 0.50 | 0.62 | 0.99 | -0.26 | 0.79 | 0.99 | 1.61 | 0.11 | 0.99 |
| **R rostral anterior cingulate** | 0.44 | 0.66 | 0.91 | 0.09 | 0.93 | 0.99 | -1.42 | 0.16 | 0.76 | -0.68 | 0.50 | 0.99 |
| **R rostral middle frontal gyrus** | 0.77 | 0.44 | 0.88 | -0.31 | 0.76 | 0.99 | -1.36 | 0.17 | 0.76 | 0.56 | 0.57 | 0.99 |
| **R STG** | 2.10 | 0.04 | 0.78 | 0.39 | 0.70 | 0.99 | -0.44 | 0.66 | 0.99 | 0.51 | 0.61 | 0.99 |
| **R superior frontal gyrus** | 1.15 | 0.25 | 0.78 | -0.87 | 0.39 | 0.99 | -1.18 | 0.24 | 0.79 | 0.88 | 0.38 | 0.99 |
| **R superior parietal cortex** | 1.43 | 0.15 | 0.78 | 0.47 | 0.64 | 0.99 | 0.42 | 0.67 | 0.99 | -1.73 | 0.08 | 0.99 |
| **R supramarginal gyrus** | 0.73 | 0.47 | 0.88 | -0.07 | 0.95 | 0.99 | -1.24 | 0.22 | 0.79 | 1.47 | 0.14 | 0.99 |
| **R temporal pole** | 0.46 | 0.65 | 0.91 | -0.18 | 0.85 | 0.99 | -0.10 | 0.92 | 0.99 | -0.52 | 0.61 | 0.99 |
| **R transverse temporal gyrus** | 1.23 | 0.22 | 0.78 | 0.67 | 0.50 | 0.99 | -0.20 | 0.84 | 0.99 | 0.37 | 0.71 | 0.99 |

**Table S17b**. Effects of Psychotropic Medications on Cortical Surface Area (t-values and p-values for comparison of 22q11DS subjects on vs. not on each category of psychotropic medication).

No significant effects were detected between psychotropic medications at the time of MRI and SA measure in any cortical regions.

**T.TA**: t-values of typical antipsychotic effects; **P.TA**: p-values of typical antipsychotic effects; **P.TA FDR**: FDR-corrected p-values of typical antipsychotic effects. **T.AA**: t-values of atypical antipsychotic effects; **P.AA**: p-values of atypical antipsychotic effects; **P.AA FDR**: FDR-corrected p-values of atypical antipsychotic effects. **T.AC**: t-values of anticonvulsant effects; **P.AC**: p-values of anticonvulsant effects; **P.AC FDR**: FDR-corrected p-values of anticonvulsant effects. **T.AD**: t-values of antidepressant effects; **P.AD**: p-values of antidepressant effects; **P.AD FDR**: FDR-corrected p-values of antidepressant effects.

# **Table S18a. Effects of Antipsychotic Medications on Cortical Thickness in 22q11DS-Subjects with Psychotic Disorder.**

| Region | p-value | FDR p-value |
| --- | --- | --- |
| R inferior temporal gyrus | 0.00 | 0.02 |
| L temporal pole | 0.00 | 0.09 |
| R temporal pole | 0.01 | 0.13 |
| L inferior temporal gyrus | 0.01 | 0.16 |
| R fusiform gyrus | 0.01 | 0.17 |
| L fusiform gyrus | 0.01 | 0.17 |
| L lateral orbitofrontal cortex | 0.03 | 0.29 |
| R entorhinal cortex | 0.03 | 0.29 |
| R isthmus cingulate | 0.06 | 0.38 |
| L medial orbitofrontal cortex | 0.06 | 0.38 |
| R pars orbitalis | 0.06 | 0.38 |
| L cuneus | 0.07 | 0.38 |
| L entorhinal cortex | 0.11 | 0.49 |
| R superior parietal cortex | 0.11 | 0.49 |
| L superior parietal cortex | 0.11 | 0.49 |
| R lateral occipital cortex | 0.12 | 0.49 |
| R pars triangularis | 0.12 | 0.49 |
| L middle temporal gyrus | 0.13 | 0.52 |
| R posterior cingulate | 0.15 | 0.55 |
| L hemisphere | 0.19 | 0.63 |
| R hemisphere | 0.19 | 0.63 |
| L pars orbitalis | 0.25 | 0.79 |
| R middle temporal gyrus | 0.26 | 0.79 |
| R inferior parietal cortex | 0.28 | 0.79 |
| L pars triangularis | 0.29 | 0.79 |
| R postcentral gyrus | 0.30 | 0.79 |
| L rostral middle frontal gyrus | 0.31 | 0.79 |
| R cuneus | 0.34 | 0.82 |
| L precuneus | 0.35 | 0.82 |
| L posterior cingulate | 0.36 | 0.82 |
| R precuneus | 0.36 | 0.82 |
| R rostral middle frontal gyrus | 0.39 | 0.82 |
| R insula | 0.40 | 0.82 |
| L transverse temporal gyrus | 0.41 | 0.82 |
| R pars opercularis | 0.42 | 0.82 |
| R lateral orbitofrontal cortex | 0.42 | 0.82 |
| R parahippocampal gyrus | 0.44 | 0.84 |
| R superior frontal gyrus | 0.46 | 0.84 |
| L precentral gyrus | 0.48 | 0.84 |
| L postcentral gyrus | 0.49 | 0.84 |
| R pericalcarine cortex | 0.50 | 0.84 |
| R banks of STS | 0.52 | 0.84 |
| L caudal middle frontal gyrus | 0.53 | 0.84 |
| L caudal anterior cingulate | 0.53 | 0.84 |
| L pars opercularis | 0.55 | 0.84 |
| L parahippocampal gyrus | 0.56 | 0.84 |
| L rostral anterior cingulate | 0.56 | 0.84 |
| L frontal pole | 0.62 | 0.88 |
| R caudal anterior cingulate | 0.63 | 0.88 |
| L superior frontal gyrus | 0.64 | 0.88 |
| L inferior parietal cortex | 0.66 | 0.88 |
| R supramarginal gyrus | 0.67 | 0.88 |
| R frontal pole | 0.67 | 0.88 |
| L insula | 0.71 | 0.88 |
| L pericalcarine cortex | 0.72 | 0.88 |
| L STG | 0.72 | 0.88 |
| R lingual gyrus | 0.74 | 0.88 |
| L lateral occipital cortex | 0.74 | 0.88 |
| R precentral gyrus | 0.74 | 0.88 |
| L lingual gyrus | 0.75 | 0.88 |
| R rostral anterior cingulate | 0.81 | 0.91 |
| R caudal middle frontal gyrus | 0.81 | 0.91 |
| L supramarginal gyrus | 0.82 | 0.91 |
| L banks of STS | 0.84 | 0.91 |
| L isthmus cingulate | 0.85 | 0.91 |
| R paracentral lobule | 0.92 | 0.97 |
| R STG | 0.93 | 0.97 |
| L paracentral lobule | 0.95 | 0.97 |
| R medial orbitofrontal cortex | 0.97 | 0.97 |
| R transverse temporal gyrus | 0.97 | 0.97 |

**Table S18a**. Effects of Antipsychotic Medications on Cortical Thickness in 22q11DS-Subjects with Psychotic Disorder. Group differences among subjects who were on typical antipsychotics, atypical antipsychotics, both types of antipsychotics, and no antipsychotics were compared using Analysis of Covariance (ANCOVA). P-values are from the overall differences among the 4 groups. Only the right inferior temporal cortex showed significant difference among all 68 regions from the analysis.

# **Table S18b. Effects of Antipsychotic Medications on Surface Area in 22q11DS Subjects with Psychotic Disorder.**

| Region | p-value | FDR p-value |
| --- | --- | --- |
| R posterior cingulate | 0.01 | 0.70 |
| R isthmus cingulate | 0.05 | 0.75 |
| R postcentral gyrus | 0.05 | 0.75 |
| L postcentral gyrus | 0.06 | 0.75 |
| R superior parietal cortex | 0.08 | 0.75 |
| R pars opercularis | 0.11 | 0.75 |
| R inferior temporal gyrus | 0.11 | 0.75 |
| R banks of STS | 0.12 | 0.75 |
| L insula | 0.12 | 0.75 |
| R caudal anterior cingulate | 0.14 | 0.75 |
| L pars opercularis | 0.14 | 0.75 |
| L superior parietal cortex | 0.15 | 0.75 |
| L posterior cingulate | 0.15 | 0.75 |
| L pars orbitalis | 0.16 | 0.75 |
| R entorhinal cortex | 0.20 | 0.75 |
| L paracentral lobule | 0.21 | 0.75 |
| L precuneus | 0.21 | 0.75 |
| L pars triangularis | 0.23 | 0.75 |
| L caudal anterior cingulate | 0.24 | 0.75 |
| L caudal middle frontal gyrus | 0.26 | 0.75 |
| R lateral orbitofrontal cortex | 0.26 | 0.75 |
| R caudal middle frontal gyrus | 0.26 | 0.75 |
| R paracentral lobule | 0.28 | 0.75 |
| R pars triangularis | 0.30 | 0.75 |
| L middle temporal gyrus | 0.30 | 0.75 |
| R pars orbitalis | 0.32 | 0.75 |
| L pericalcarine cortex | 0.32 | 0.75 |
| R precuneus | 0.33 | 0.75 |
| L inferior temporal gyrus | 0.34 | 0.75 |
| L precentral gyrus | 0.34 | 0.75 |
| L inferior parietal cortex | 0.36 | 0.75 |
| L lateral occipital cortex | 0.38 | 0.75 |
| R cuneus | 0.38 | 0.75 |
| L lateral orbitofrontal cortex | 0.38 | 0.75 |
| R precentral gyrus | 0.39 | 0.75 |
| R medial orbitofrontal cortex | 0.41 | 0.75 |
| L banks of STS | 0.42 | 0.75 |
| L entorhinal cortex | 0.43 | 0.75 |
| L hemisphere | 0.43 | 0.75 |
| L rostral middle frontal gyrus | 0.44 | 0.75 |
| R STG | 0.46 | 0.75 |
| R temporal pole | 0.47 | 0.75 |
| R pericalcarine cortex | 0.49 | 0.75 |
| R frontal pole | 0.50 | 0.75 |
| L STG | 0.51 | 0.75 |
| R supramarginal gyrus | 0.51 | 0.75 |
| R hemisphere | 0.51 | 0.75 |
| L temporal pole | 0.51 | 0.75 |
| L transverse temporal gyrus | 0.54 | 0.77 |
| R middle temporal gyrus | 0.55 | 0.77 |
| R rostral middle frontal gyrus | 0.59 | 0.81 |
| R rostral anterior cingulate | 0.61 | 0.82 |
| L isthmus cingulate | 0.62 | 0.82 |
| L medial orbitofrontal cortex | 0.66 | 0.85 |
| R parahippocampal gyrus | 0.68 | 0.86 |
| R inferior parietal cortex | 0.69 | 0.86 |
| R lingual gyrus | 0.74 | 0.89 |
| L fusiform gyrus | 0.75 | 0.89 |
| R transverse temporal gyrus | 0.76 | 0.89 |
| L cuneus | 0.76 | 0.89 |
| R fusiform gyrus | 0.78 | 0.89 |
| L superior frontal gyrus | 0.80 | 0.90 |
| L frontal pole | 0.81 | 0.90 |
| R lateral occipital cortex | 0.85 | 0.92 |
| L supramarginal gyrus | 0.86 | 0.92 |
| L rostral anterior cingulate | 0.89 | 0.94 |
| L parahippocampal gyrus | 0.91 | 0.94 |
| L lingual gyrus | 0.91 | 0.94 |
| R superior frontal gyrus | 0.95 | 0.96 |
| R insula | 0.96 | 0.96 |

**Table S18b**. Effects of Antipsychotic Medications on Surface Area in 22q11DS Subjects with Psychotic Disorder. Group differences among subjects who were on typical antipsychotics, atypical antipsychotics, both types of antipsychotics, and no antipsychotics were compared using Analysis of Covariance (ANCOVA). P-values are from the overall differences among the 4 groups. No significant difference was found in any cortical regions from the analysis.

# **Table S19a. Effects of covarying for handedness on CT (Effect sizes and p-values)**

| Region | Cohen’s d  HD unadjust. | Cohen’d  HD adjust. | p-FDR  HD unadjust. | p-FDR  HD adjust. |
| --- | --- | --- | --- | --- |
| L parahippocampal gyrus | -0.5669 | -0.5676 | 5.190E-11 | 4.760E-11 |
| L STG | -0.4978 | -0.4826 | 6.120E-09 | 1.670E-08 |
| R parahippocampal gyrus | -0.3096 | -0.3109 | 2.960E-04 | 2.740E-04 |
| R STG | -0.3033 | -0.2956 | 3.890E-04 | 5.380E-04 |
| L transverse temporal gyrus | -0.2452 | -0.2384 | 4.243E-03 | 5.261E-03 |
| L caudal anterior cingulate | -0.1860 | -0.1837 | 2.995E-02 | 3.208E-02 |
| R posterior cingulate | -0.1441 | -0.1378 | 9.495E-02 | 1.077E-01 |
| L posterior cingulate | -0.1227 | -0.1181 | 1.536E-01 | 1.705E-01 |
| R isthmus cingulate | -0.0813 | -0.0849 | 3.588E-01 | 3.350E-01 |
| L temporal pole | -0.0547 | -0.0488 | 5.534E-01 | 6.040E-01 |
| L banks of STS | -0.0203 | -0.0226 | 8.408E-01 | 8.178E-01 |
| R caudal anterior cingulate | -0.0091 | -0.0047 | 9.299E-01 | 9.541E-01 |
| R entorhinal cortex | -0.0086 | -0.0132 | 9.299E-01 | 8.978E-01 |
| R banks of STS | 0.0046 | 0.0112 | 9.549E-01 | 9.049E-01 |
| R transverse temporal gyrus | 0.0267 | 0.0300 | 7.910E-01 | 7.587E-01 |
| R temporal pole | 0.0296 | 0.0334 | 7.740E-01 | 7.366E-01 |
| R lateral occipital cortex | 0.1315 | 0.1412 | 1.263E-01 | 1.003E-01 |
| L frontal pole | 0.1402 | 0.1423 | 1.033E-01 | 9.926E-02 |
| R rostral anterior cingulate | 0.1545 | 0.1592 | 7.321E-02 | 6.398E-02 |
| L lateral occipital cortex | 0.1572 | 0.1640 | 6.904E-02 | 5.675E-02 |
| R frontal pole | 0.1919 | 0.2012 | 2.532E-02 | 1.888E-02 |
| L entorhinal cortex | 0.1938 | 0.1985 | 2.424E-02 | 2.028E-02 |
| L fusiform gyrus | 0.2050 | 0.2157 | 1.705E-02 | 1.190E-02 |
| L rostral anterior cingulate | 0.2093 | 0.2135 | 1.497E-02 | 1.260E-02 |
| L superior parietal cortex | 0.2355 | 0.2398 | 5.923E-03 | 5.082E-03 |
| R inferior parietal cortex | 0.2423 | 0.2464 | 4.659E-03 | 4.002E-03 |
| L inferior parietal cortex | 0.2569 | 0.2633 | 2.714E-03 | 2.066E-03 |
| R superior parietal cortex | 0.2637 | 0.2691 | 2.089E-03 | 1.655E-03 |
| R fusiform gyrus | 0.2665 | 0.2740 | 1.906E-03 | 1.371E-03 |
| L inferior temporal gyrus | 0.2938 | 0.2965 | 5.900E-04 | 5.270E-04 |
| L isthmus cingulate | 0.3177 | 0.3186 | 2.040E-04 | 1.940E-04 |
| R inferior temporal gyrus | 0.3332 | 0.3355 | 9.710E-05 | 8.520E-05 |
| L lingual gyrus | 0.3874 | 0.3943 | 5.290E-06 | 3.660E-06 |
| L middle temporal gyrus | 0.3882 | 0.3907 | 5.180E-06 | 4.410E-06 |
| R pars orbitalis | 0.3882 | 0.3879 | 5.180E-06 | 5.060E-06 |
| R pars opercularis | 0.3940 | 0.4009 | 3.900E-06 | 2.550E-06 |
| L precuneus | 0.4079 | 0.4091 | 1.760E-06 | 1.600E-06 |
| R postcentral gyrus | 0.4194 | 0.4304 | 9.000E-07 | 4.690E-07 |
| R middle temporal gyrus | 0.4199 | 0.4292 | 8.950E-07 | 4.910E-07 |
| R precentral gyrus | 0.4219 | 0.4269 | 8.170E-07 | 5.500E-07 |
| R lateral orbitofrontal cortex | 0.4378 | 0.4381 | 3.100E-07 | 2.950E-07 |
| L precentral gyrus | 0.4536 | 0.4607 | 1.140E-07 | 6.980E-08 |
| L pars orbitalis | 0.4629 | 0.4663 | 6.370E-08 | 4.950E-08 |
| L cuneus | 0.4908 | 0.4949 | 9.680E-09 | 7.300E-09 |
| L lateral orbitofrontal cortex | 0.5035 | 0.5096 | 4.210E-09 | 2.830E-09 |
| R precuneus | 0.5044 | 0.5079 | 4.100E-09 | 2.980E-09 |
| R pars triangularis | 0.5060 | 0.5078 | 3.810E-09 | 2.980E-09 |
| R lingual gyrus | 0.5106 | 0.5203 | 2.840E-09 | 1.350E-09 |
| L paracentral lobule | 0.5176 | 0.5230 | 1.770E-09 | 1.150E-09 |
| L superior frontal gyrus | 0.5279 | 0.5321 | 8.630E-10 | 6.070E-10 |
| R paracentral lobule | 0.5345 | 0.5382 | 5.480E-10 | 4.010E-10 |
| L pars triangularis | 0.5421 | 0.5472 | 3.250E-10 | 2.110E-10 |
| R cuneus | 0.5601 | 0.5660 | 8.450E-11 | 5.090E-11 |
| R superior frontal gyrus | 0.5683 | 0.5712 | 4.930E-11 | 3.780E-11 |
| L caudal middle frontal gyrus | 0.5684 | 0.5728 | 4.930E-11 | 3.530E-11 |
| L medial orbitofrontal cortex | 0.5922 | 0.5948 | 8.100E-12 | 6.250E-12 |
| R insula | 0.6081 | 0.6152 | 2.310E-12 | 1.210E-12 |
| L supramarginal gyrus | 0.6228 | 0.6294 | 7.150E-13 | 3.880E-13 |
| L postcentral gyrus | 0.6258 | 0.6306 | 6.010E-13 | 3.800E-13 |
| L pars opercularis | 0.6259 | 0.6328 | 6.010E-13 | 3.420E-13 |
| R medial orbitofrontal cortex | 0.6344 | 0.6356 | 3.430E-13 | 2.940E-13 |
| L hemisphere | 0.6420 | 0.6493 | 1.960E-13 | 9.790E-14 |
| R pericalcarine cortex | 0.6589 | 0.6692 | 4.920E-14 | 1.850E-14 |
| R hemisphere | 0.6663 | 0.6735 | 2.900E-14 | 1.430E-14 |
| R caudal middle frontal gyrus | 0.6825 | 0.6817 | 7.750E-15 | 7.870E-15 |
| L pericalcarine cortex | 0.6919 | 0.6977 | 3.900E-15 | 2.150E-15 |
| R supramarginal gyrus | 0.7324 | 0.7387 | 1.040E-16 | 5.300E-17 |
| L rostral middle frontal gyrus | 0.7433 | 0.7504 | 4.810E-17 | 2.230E-17 |
| R rostral middle frontal gyrus | 0.8385 | 0.8421 | 4.160E-21 | 2.610E-21 |
| L insula | 0.9067 | 0.9105 | 4.670E-24 | 2.760E-24 |

**Table S19a.** Effects of Covarying for Handedness on CT (Effect Sizes and P-Values). Effect size values in Cohen’s d were shown here for the group differences in CT between 22q11DS cases and controls, contrasting those from the statistical model that adjusted for handedness with that did not adjust for it, in order to examine the influence of handedness on group differences in CT. As indicated, Cohen’s d values and the significance levels remain almost the same whether handedness was adjusted for or not.

# **Table S19b. Effects of covarying for handedness on SA (Effect sizes and p-values)**

| Region | Cohen’s d  HD unadjust. | Cohen’d  HD adjust. | p-FDR  HD unadjust. | p-FDR  HD adjust. |
| --- | --- | --- | --- | --- |
| L banks of STS | -0.3488 | -0.3461 | 3.946E-05 | 4.365E-05 |
| L caudal anterior cingulate | -1.1600 | -1.1599 | 2.117E-38 | 1.870E-38 |
| L caudal middle frontal gyrus | -0.0663 | -0.0688 | 4.390E-01 | 4.206E-01 |
| L cuneus | -1.5383 | -1.5422 | 3.964E-60 | 1.789E-60 |
| L entorhinal cortex | -0.4362 | -0.4351 | 3.051E-07 | 3.196E-07 |
| L frontal pole | -0.1916 | -0.1874 | 2.464E-02 | 2.802E-02 |
| L fusiform gyrus | -1.0250 | -1.0285 | 2.985E-31 | 1.732E-31 |
| L hemisphere | -1.0892 | -1.0930 | 1.238E-34 | 6.246E-35 |
| L inferior parietal cortex | -0.0702 | -0.0742 | 4.171E-01 | 3.891E-01 |
| L inferior temporal gyrus | -0.7143 | -0.7219 | 9.859E-17 | 4.707E-17 |
| L insula | 0.2827 | 0.2794 | 8.554E-04 | 9.763E-04 |
| L isthmus cingulate | -0.1548 | -0.1557 | 7.022E-02 | 6.818E-02 |
| L lateral occipital cortex | -0.8304 | -0.8300 | 1.033E-21 | 1.002E-21 |
| L lateral orbitofrontal cortex | -0.2102 | -0.2113 | 1.426E-02 | 1.361E-02 |
| L lingual gyrus | -1.3990 | -1.3989 | 4.150E-52 | 3.486E-52 |
| L medial orbitofrontal cortex | -0.1794 | -0.1731 | 3.548E-02 | 4.276E-02 |
| L middle temporal gyrus | -0.5048 | -0.5094 | 3.128E-09 | 2.186E-09 |
| L paracentral lobule | -0.2917 | -0.2901 | 5.961E-04 | 6.329E-04 |
| L parahippocampal gyrus | 0.0870 | 0.0833 | 3.125E-01 | 3.349E-01 |
| L pars opercularis | -0.2019 | -0.2035 | 1.855E-02 | 1.748E-02 |
| L pars orbitalis | -0.2901 | -0.2871 | 6.259E-04 | 7.082E-04 |
| L pars triangularis | -0.5311 | -0.5377 | 5.141E-10 | 3.134E-10 |
| L pericalcarine cortex | -1.1998 | -1.2008 | 1.306E-40 | 9.837E-41 |
| L postcentral gyrus | -0.6916 | -0.6951 | 7.981E-16 | 5.446E-16 |
| L posterior cingulate | -0.4150 | -0.4125 | 1.086E-06 | 1.237E-06 |
| L precentral gyrus | 0.4247 | 0.4232 | 6.103E-07 | 6.582E-07 |
| L precuneus | -1.3946 | -1.3956 | 6.171E-52 | 4.408E-52 |
| L rostral anterior cingulate | -0.7181 | -0.7150 | 7.206E-17 | 8.724E-17 |
| L rostral middle frontal gyrus | -0.8934 | -0.8959 | 1.219E-24 | 8.414E-25 |
| L STG | -0.3979 | -0.4010 | 2.794E-06 | 2.290E-06 |
| L superior frontal gyrus | -0.3639 | -0.3620 | 1.838E-05 | 2.008E-05 |
| L superior parietal cortex | -1.1240 | -1.1262 | 1.964E-36 | 1.288E-36 |
| L supramarginal gyrus | -0.1584 | -0.1641 | 6.462E-02 | 5.468E-02 |
| L temporal pole | -0.6109 | -0.6139 | 9.889E-13 | 7.279E-13 |
| L transverse temporal gyrus | -0.4044 | -0.4061 | 1.951E-06 | 1.724E-06 |
| R banks of STS | -0.2382 | -0.2376 | 5.230E-03 | 5.315E-03 |
| R caudal anterior cingulate | -0.8671 | -0.8623 | 2.093E-23 | 3.240E-23 |
| R caudal middle frontal gyrus | -0.1079 | -0.1103 | 2.075E-01 | 1.965E-01 |
| R cuneus | -1.4576 | -1.4593 | 1.399E-55 | 8.903E-56 |
| R entorhinal cortex | -0.5301 | -0.5303 | 5.365E-10 | 5.118E-10 |
| R frontal pole | 0.0013 | 0.0022 | 9.875E-01 | 9.784E-01 |
| R fusiform gyrus | -1.0869 | -1.0932 | 1.530E-34 | 6.246E-35 |
| R hemisphere | -1.0960 | -1.0983 | 5.742E-35 | 3.768E-35 |
| R inferior parietal cortex | -0.1404 | -0.1396 | 9.954E-02 | 1.013E-01 |
| R inferior temporal gyrus | -0.7639 | -0.7690 | 8.491E-19 | 4.746E-19 |
| R insula | 0.5124 | 0.5143 | 1.868E-09 | 1.569E-09 |
| R isthmus cingulate | -0.0268 | -0.0192 | 7.667E-01 | 8.390E-01 |
| R lateral occipital cortex | -0.8083 | -0.8135 | 1.006E-20 | 5.417E-21 |
| R lateral orbitofrontal cortex | -0.1177 | -0.1207 | 1.697E-01 | 1.579E-01 |
| R lingual gyrus | -1.4825 | -1.4831 | 6.001E-57 | 4.454E-57 |
| R medial orbitofrontal cortex | -0.3459 | -0.3458 | 4.497E-05 | 4.365E-05 |
| R middle temporal gyrus | -0.4059 | -0.4067 | 1.831E-06 | 1.711E-06 |
| R paracentral lobule | -0.5152 | -0.5148 | 1.569E-09 | 1.567E-09 |
| R parahippocampal gyrus | -0.1969 | -0.2003 | 2.150E-02 | 1.876E-02 |
| R pars opercularis | 0.0070 | 0.0071 | 9.460E-01 | 9.451E-01 |
| R pars orbitalis | -0.1954 | -0.2004 | 2.218E-02 | 1.876E-02 |
| R pars triangularis | -0.5757 | -0.5756 | 1.717E-11 | 1.662E-11 |
| R pericalcarine cortex | -1.2054 | -1.2072 | 7.172E-41 | 4.842E-41 |
| R postcentral gyrus | -0.7943 | -0.7981 | 4.076E-20 | 2.567E-20 |
| R posterior cingulate | -0.3352 | -0.3326 | 7.513E-05 | 8.444E-05 |
| R precentral gyrus | 0.3431 | 0.3465 | 5.107E-05 | 4.365E-05 |
| R precuneus | -1.3698 | -1.3704 | 1.651E-50 | 1.260E-50 |
| R rostral anterior cingulate | -0.4956 | -0.4882 | 5.864E-09 | 9.634E-09 |
| R rostral middle frontal gyrus | -0.9602 | -0.9639 | 6.272E-28 | 3.686E-28 |
| R STG | -0.3922 | -0.3933 | 3.820E-06 | 3.517E-06 |
| R superior frontal gyrus | -0.1502 | -0.1478 | 7.809E-02 | 8.297E-02 |
| R superior parietal cortex | -1.1135 | -1.1162 | 6.781E-36 | 4.223E-36 |
| R supramarginal gyrus | -0.4858 | -0.4855 | 1.136E-08 | 1.128E-08 |
| R temporal pole | -0.5353 | -0.5348 | 3.884E-10 | 3.771E-10 |
| R transverse temporal gyrus | -0.3526 | -0.3564 | 3.303E-05 | 2.654E-05 |

**Table S19b.** Effects of Covarying for Handedness on SA (Effect Sizes and P-Values). Effect size values in Cohen’s d were shown here for the group differences in SA between 22q11DS cases and controls, contrasting those from the statistical model that adjusted for handedness with that did not adjust for it, in order to examine the influence of handedness on group differences in SA. As indicated, Cohen’s d values and the significance levels remain almost the same whether handedness was adjusted for or not.
