# Supplementary material for: Large-scale mapping of cortical alterations in 22q11.2 deletion syndrome: Convergence with idiopathic psychosis and effects of deletion size
Source: Mol Psychiatry. 2018 Jun 13;25(8):1822–34. doi: 10.1038/s41380-018-0078-5 (PMC6292748; doi:10.1038/s41380-018-0078-5)
Supplement: Supplementary file 4 — Supplement 4 [file 41380_2018_78_MOESM4_ESM.docx]

Supplementary Figures- Table of Contents

[Figure S1a. Age Effects on Cortical Thickness for 22q11DS vs Control Comparison 3](#_Toc507515379)

[Figure S1b. Age Effects on Cortical Surface Area for 22q11DS vs Control Comparison 5](#_Toc507515380)

[Figure S2. Relationships between Total Intracranial Volume (ICV) and Global CT/SA 6](#_Toc507515381)

[Figure S3. Differences in Global Brain Measures, by Study Site 7](#_Toc507515382)

[Figure S4a. Scatterplots of Regional Differences in Cortical Thickness between 22q11DS and Control Subjects 9](#_Toc507515383)

[Figure S4b. Scatterplots of Regional Differences in Surface Area between 22q11DS and Control Subjects 11](#_Toc507515384)

[Figure S5a. Scatterplots of Regional Cortical Thickness Differences between 22q11DS+Psychosis vs. 22q11DS-No Psychosis Subjects 13](#_Toc507515385)

[Figure S5b. Scatterplots of Regional Surface Area Differences between 22q11DS+Psychosis vs. 22q11DS-No Psychosis Subjects 15](#_Toc507515386)

[Figure S6a. Scatterplots of Regional Cortical Thickness Differences between A-B Deletion, A-D Deletion and Control Subjects 17](#_Toc507515387)

[Figure S6b. Scatterplots of Regional Surface Area Differences between A-B Deletion, A-D Deletion and Control Subjects 19](#_Toc507515388)

[Figure S7a. IQ Adjusted and Unadjusted Results (Cohen’s d) for CT: 22q11DS Cases vs. Controls 21](#_Toc507515389)

[Figure S7b. IQ Adjusted and Unadjusted Results (Cohen’s d) for SA: 22q11DS Cases vs. Controls 23](#_Toc507515390)

[Figure S8. IQ Adjusted and Unadjusted Results (Cohen’s d) for CT: 22q11DS cases with psychosis and without psychosis 25](#_Toc507515391)

**Supplementary Figures**

Ggplot2, a R package, was used for the plotting and curve fitting for all the below supplementary figures. All curve fitting was done with Locally Weighted Scatter-plot Smoother (LOESS) smoothing (Cleveland, Grosse & Shyu 1992).


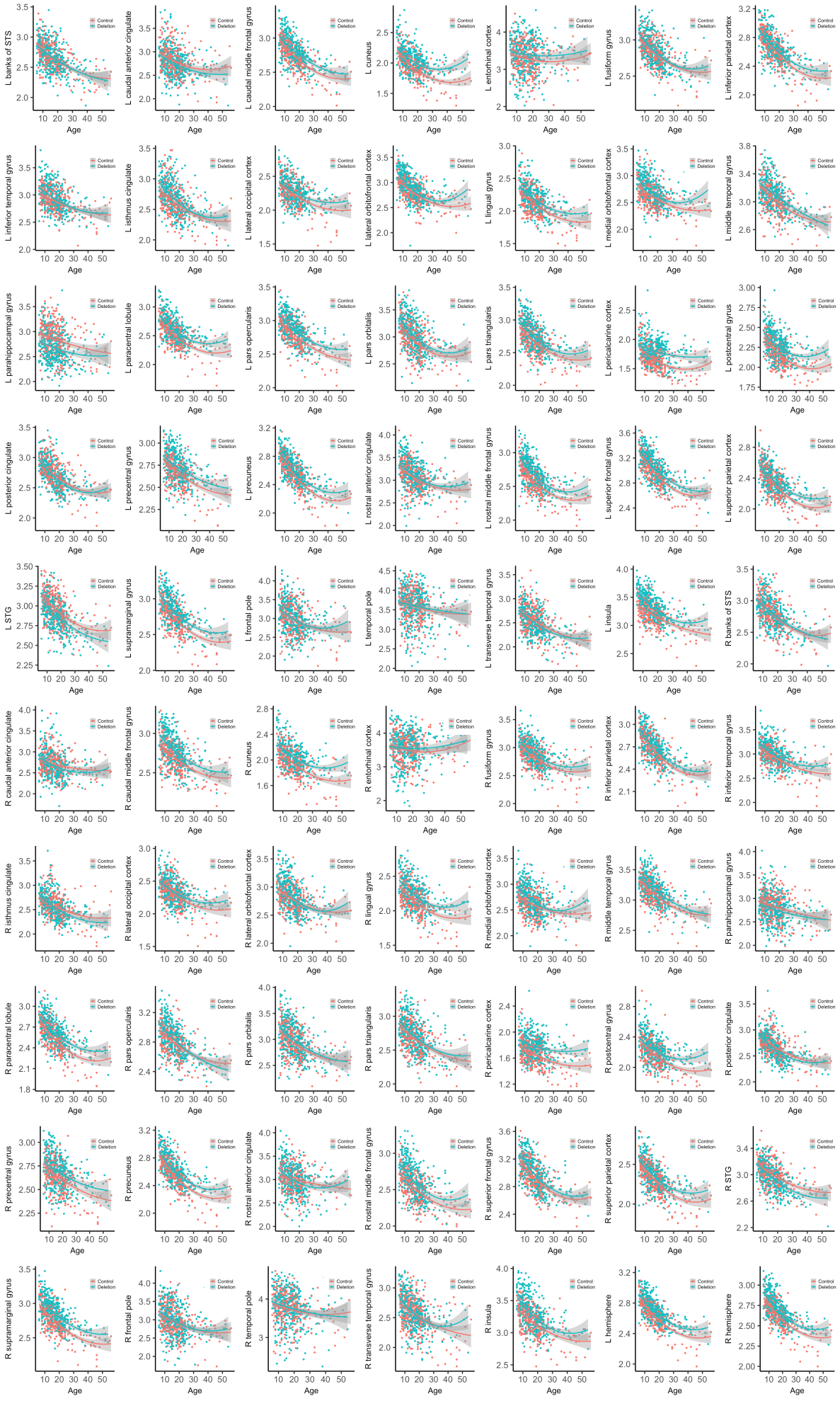


Figure S1a. Age Effects on Cortical Thickness for 22q11DS vs Control Comparison (above). A non-linear age effect can be observed in the majority of the regions and both hemispheres, which is further confirmed by comparing models with and without a quadratic age term (Table S3a). No clear pattern of group-age interactions can be seen (confirmed in Table 5a).


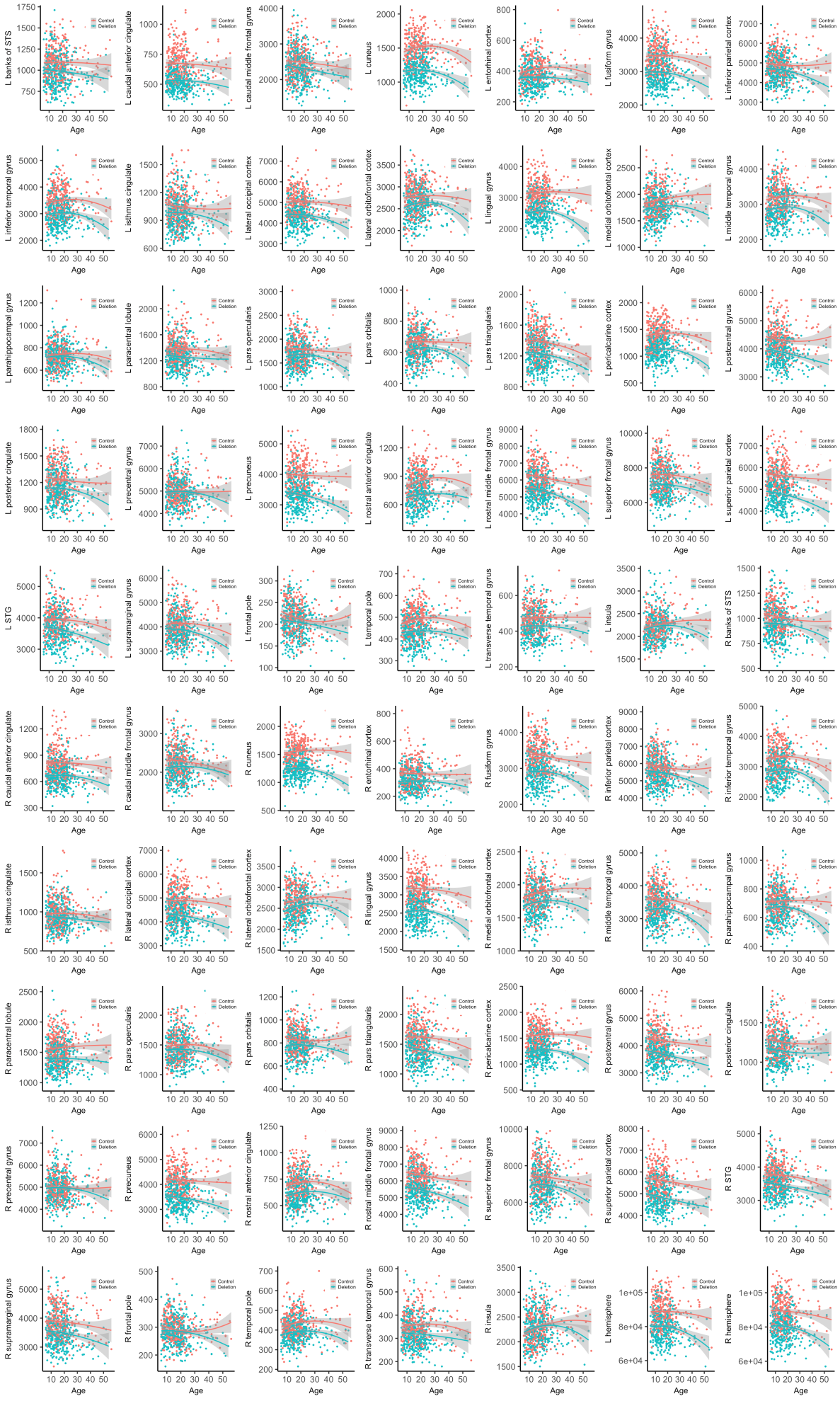


Figure S1b. Age Effects on Cortical Surface Area for 22q11DS vs Control Comparison (above). No clear non-linear age effect is observed in the majority of the regions or either hemisphere, which is further confirmed by comparing models with and without a quadratic age term (Table S3b). No clear pattern of group-age interactions can be seen (confirmed in Table 5b).


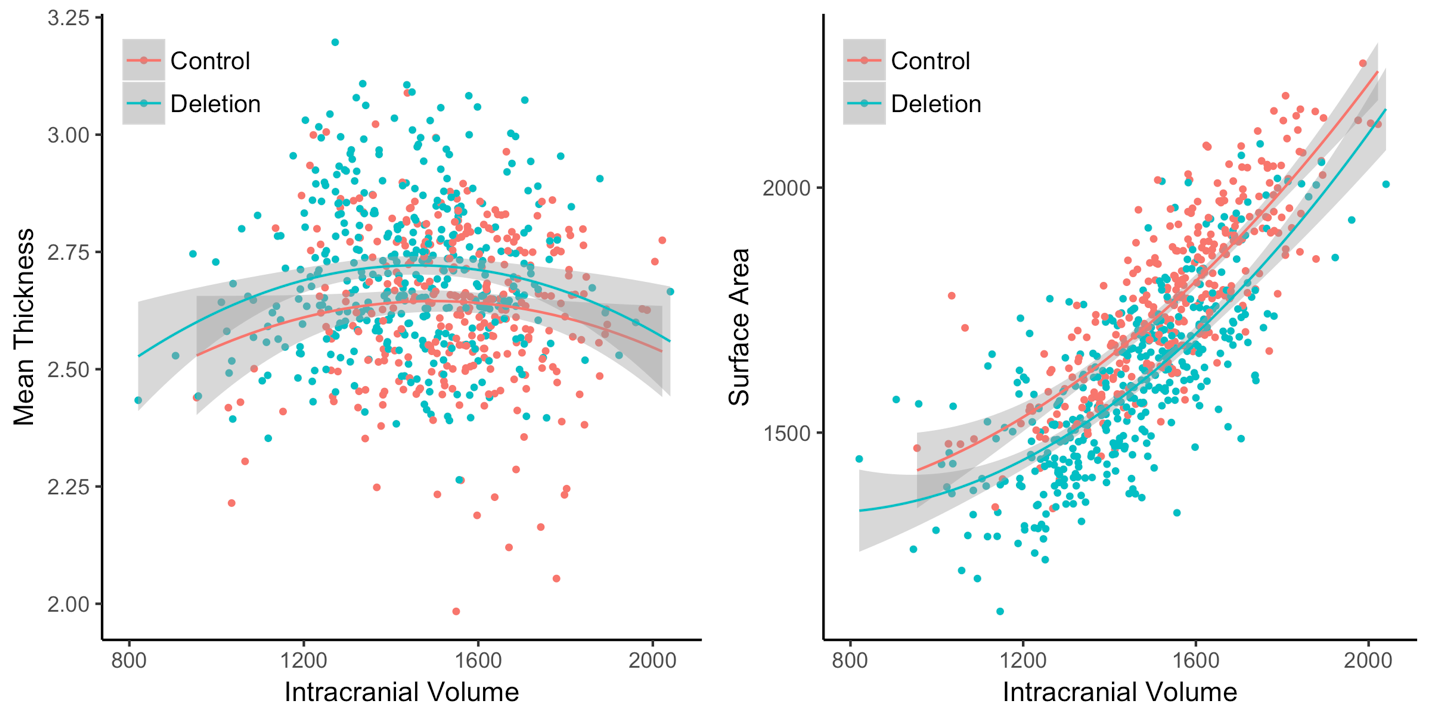


Figure S2. Relationships between Total Intracranial Volume (ICV) and Global CT/SA. The relationships were fitted between ICV and CT averaged across both hemispheres (left panel), and between ICV and total cortical surface area (right panel), respectively. No linear effect is observed for CT, while a clear linear effect is seen for SA.


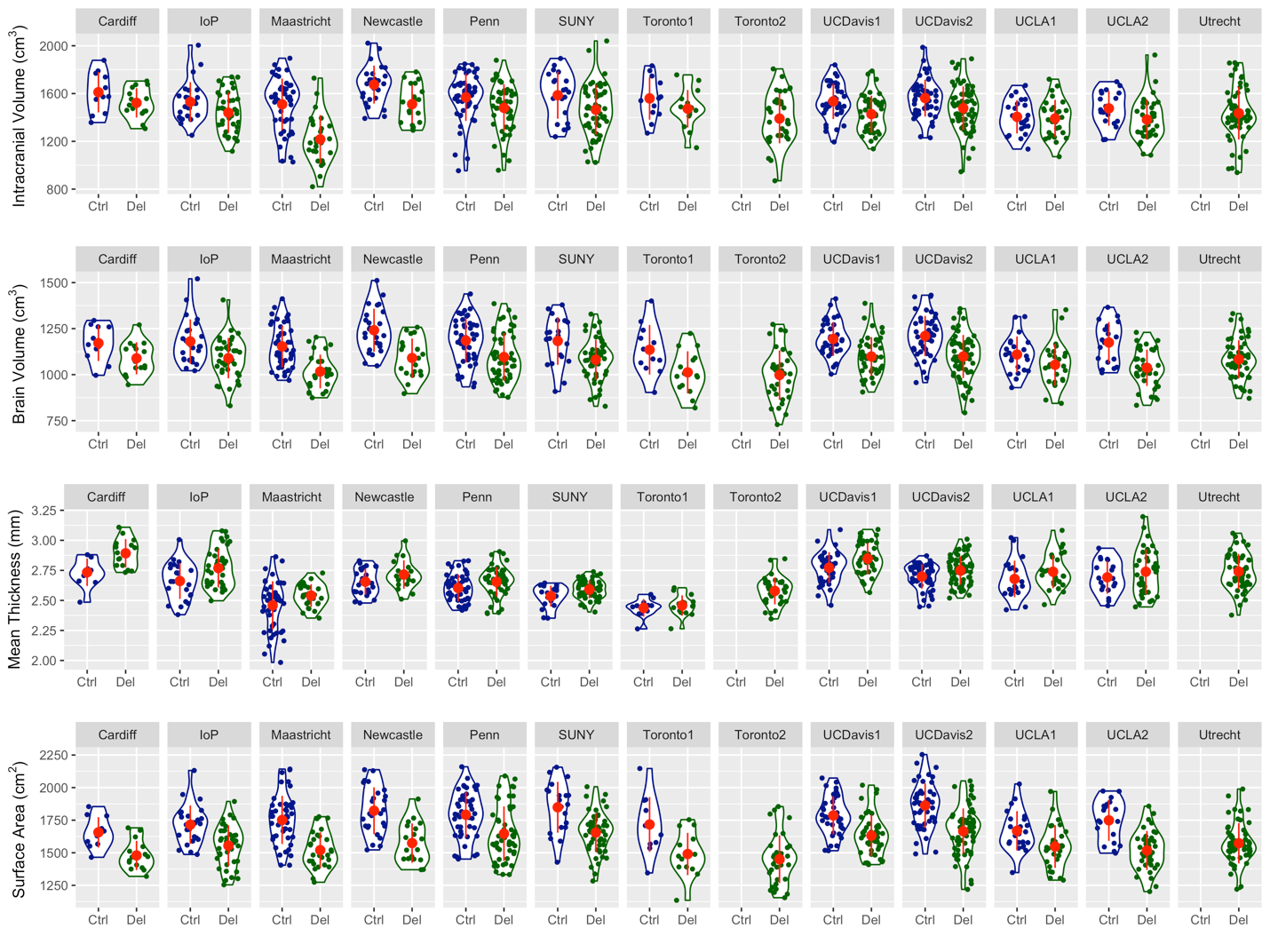


Figure S3. Differences in Global Brain Measures, by Study Site. Scatter- and violin-plots were made to show the distribution of four global brain measures (ICV, total brain volume, mean cortical thickness, and total cortical surface area) for each of the 13 datasets from 10 study sites. Despite cross-site variations, consistent patterns of group differences are clearly observed. Red dots represent group means, and red vertical lines represent within-group standard deviations.


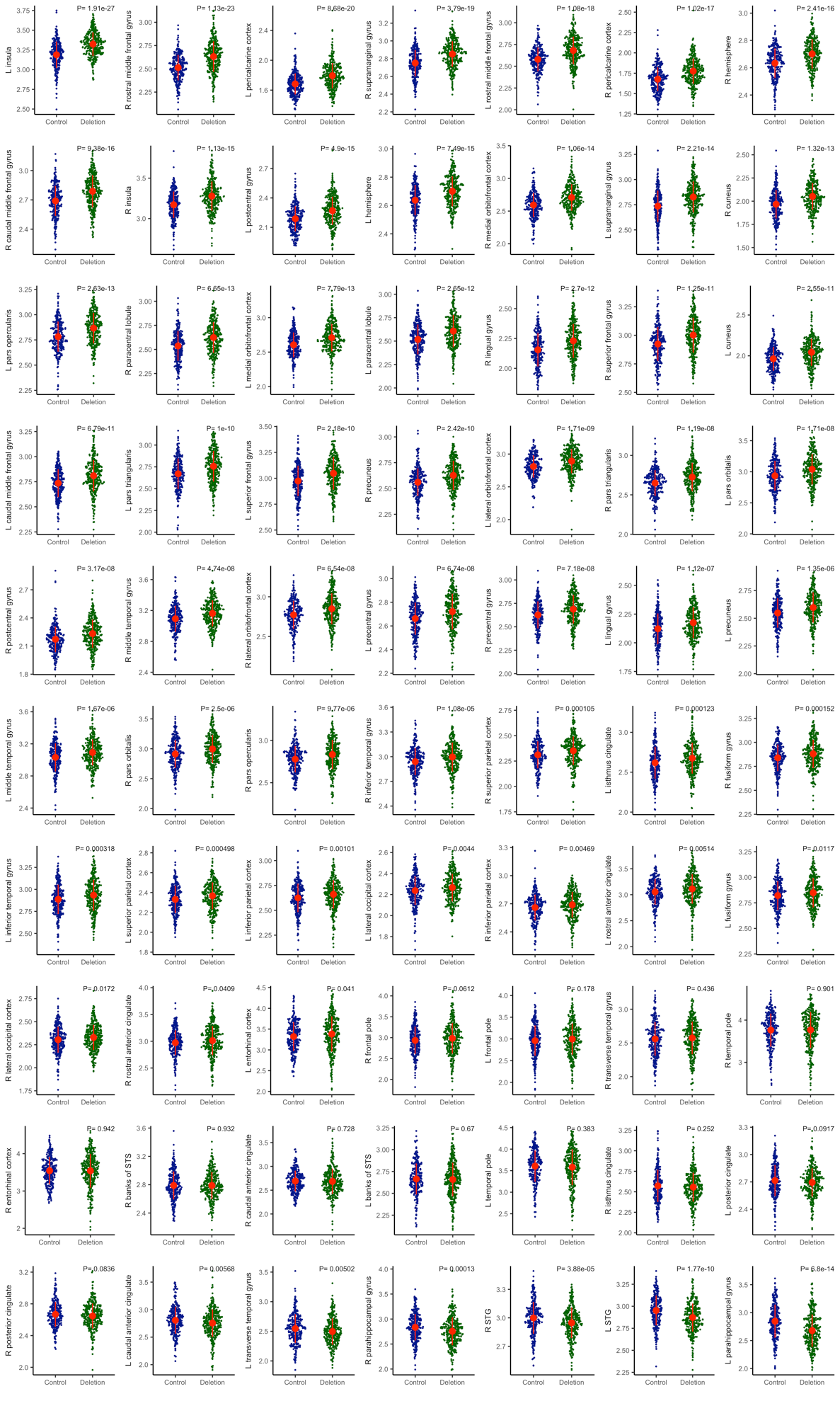


Figure S4a. Scatterplots of Regional Differences in Cortical Thickness between 22q11DS and Control Subjects (above). The data-points were adjusted for study site, sex, and both linear and quadratic age terms. Red dots represent group means, and red vertical lines represent with-group standard deviations of the residuals from the above modeling.


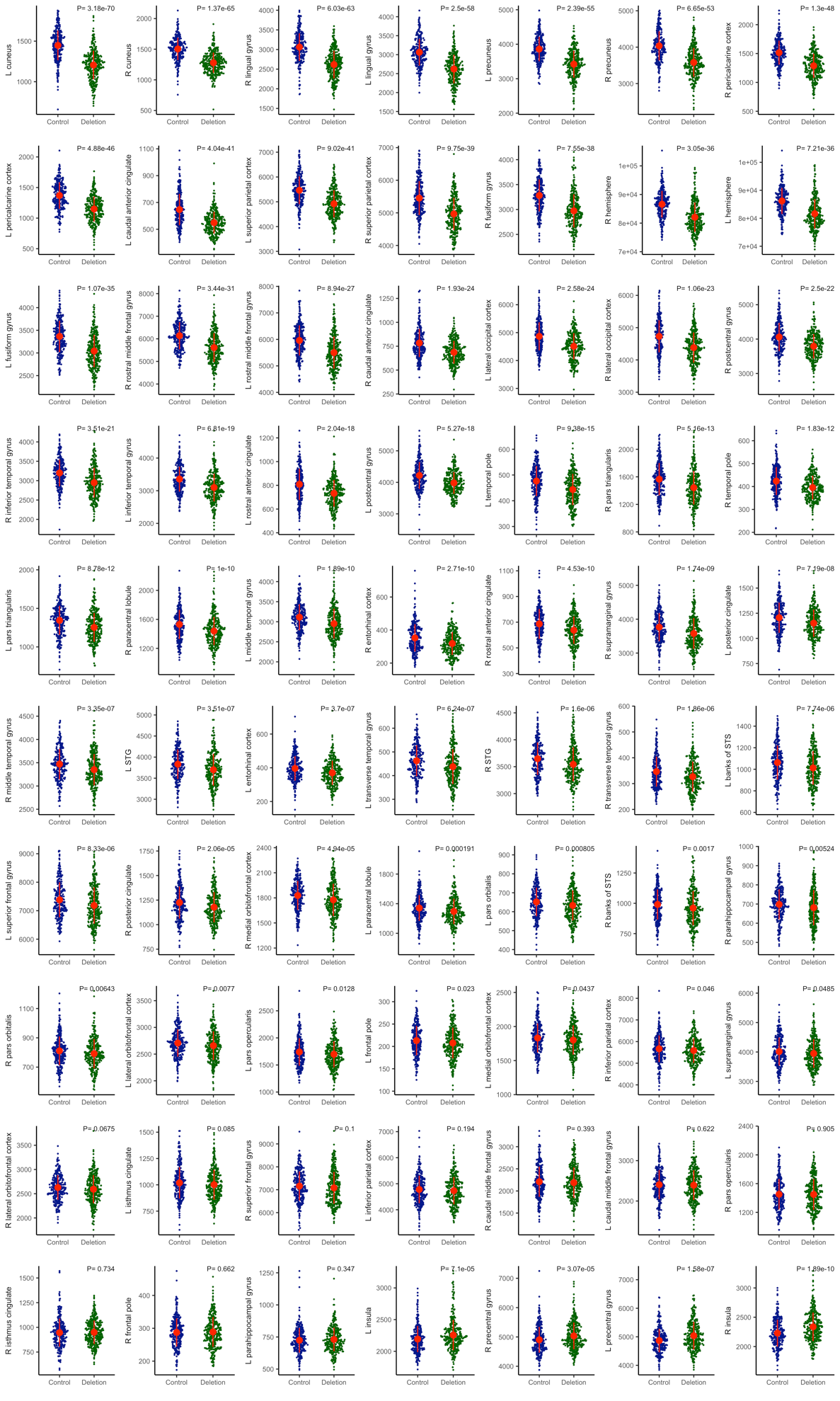


Figure S4b. Scatterplots of Regional Differences in Surface Area between 22q11DS and Control Subjects (above). The data-points were adjusted for study site, sex, age and ICV. Red dots represent group means, and red vertical lines represent within-group standard deviations of the residuals from the above modeling.


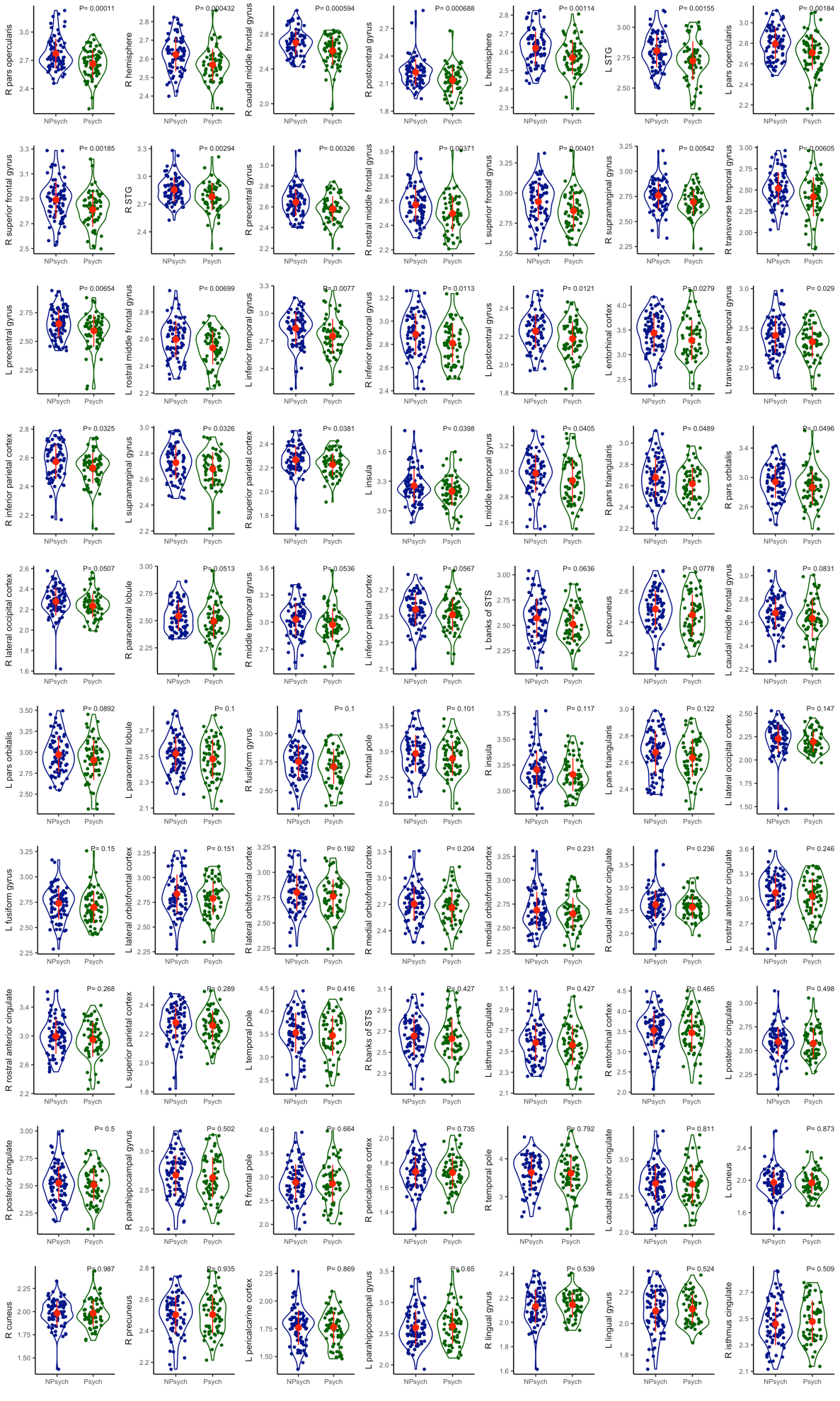


Figure S5a. Scatterplots of Regional Cortical Thickness Differences between 22q11DS+Psychosis vs. 22q11DS-No Psychosis Subjects (above). The data-points were adjusted for study site, sex, and age. Red dots represent group means, and red vertical lines represent within-group standard deviations of the residuals from the above modeling.


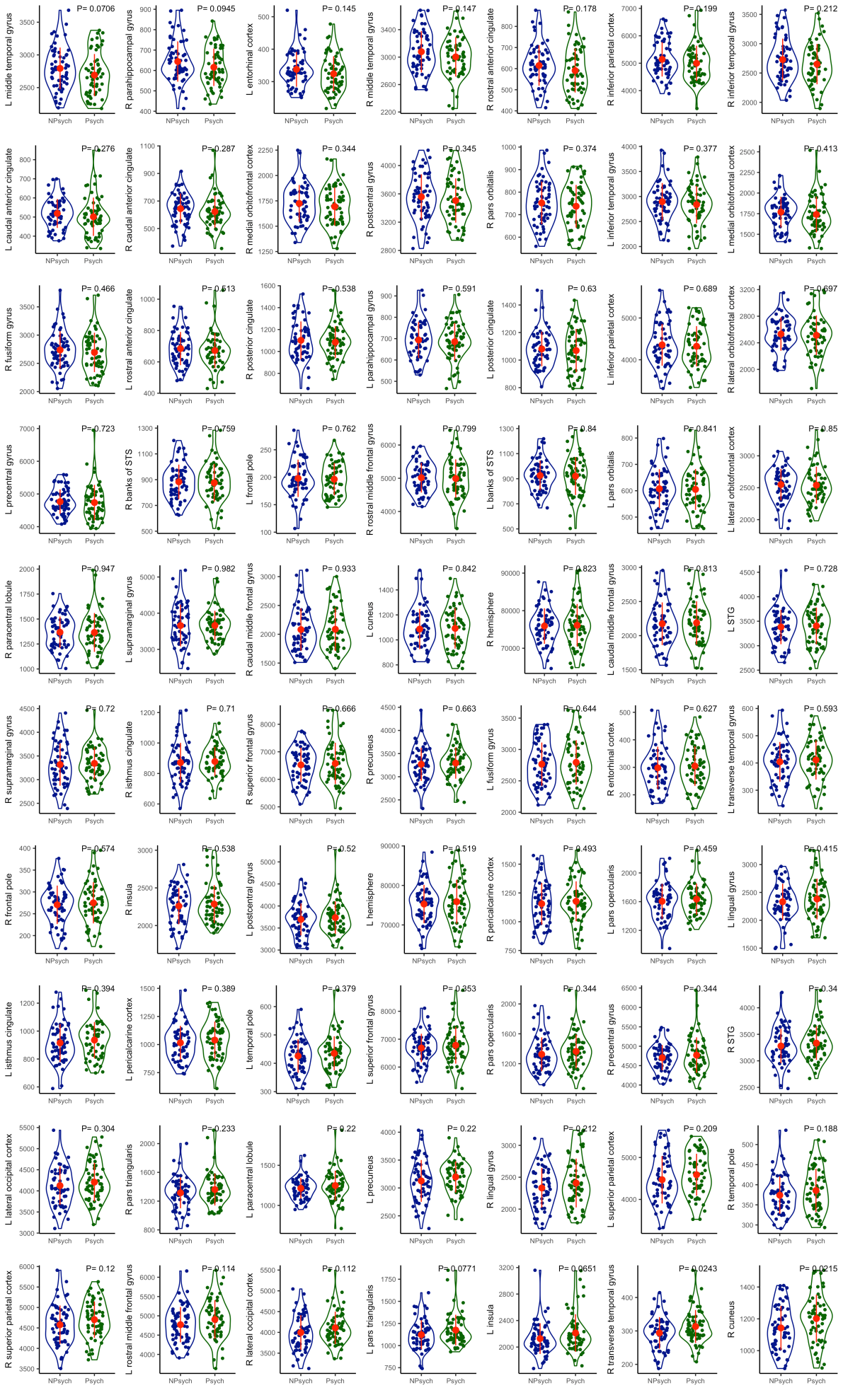


Figure S5b. Scatterplots of Regional Surface Area Differences between 22q11DS+Psychosis vs. 22q11DS-No Psychosis Subjects (above). The data-points were adjusted for study site, sex, age, and ICV. Red dots represent group means, and red vertical lines represent within-group standard deviations of the residuals from the above modeling.


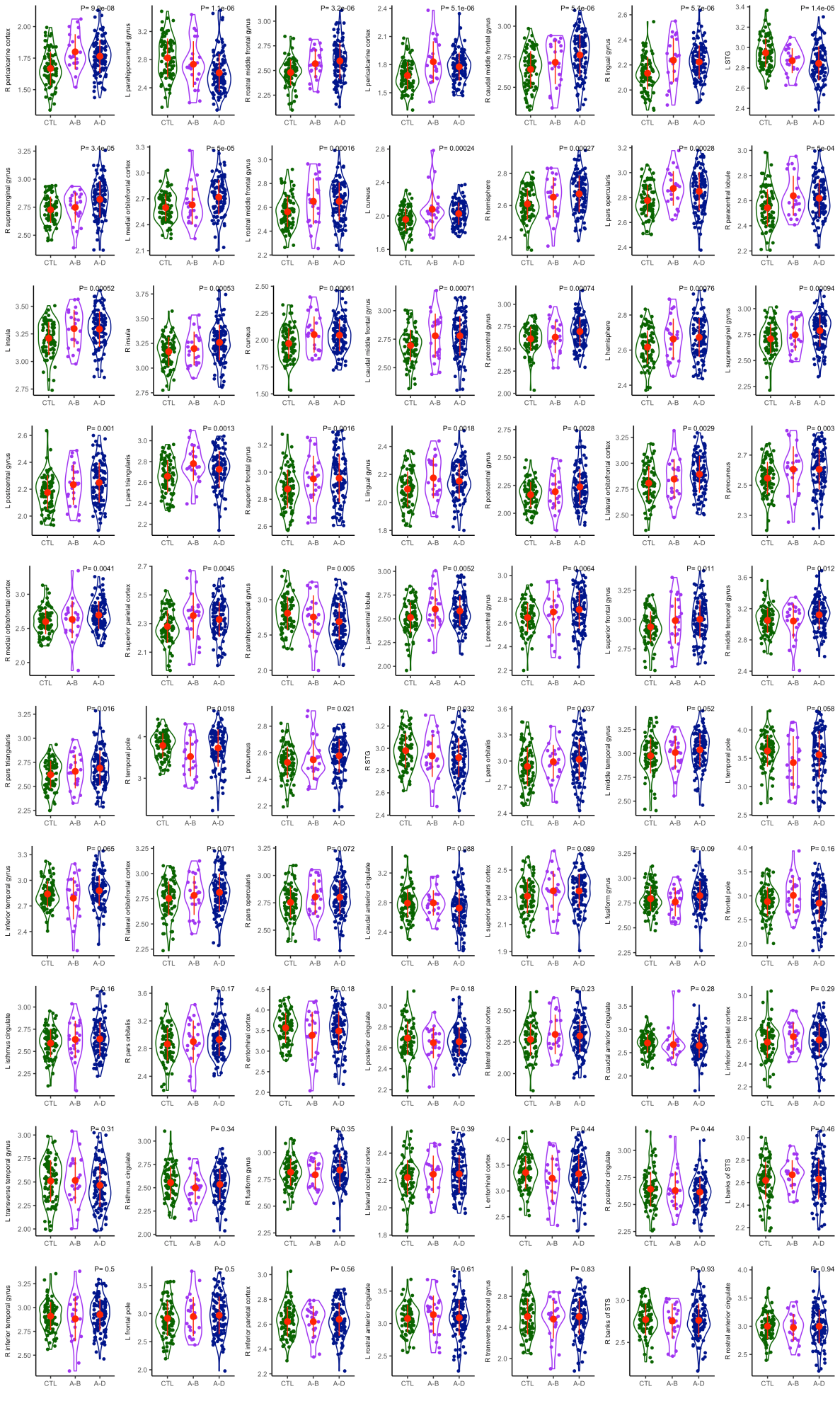


Figure S6a. Scatterplots of Regional Cortical Thickness Differences between A-B Deletion, A-D Deletion and Control Subjects (above). The data-points were adjusted for study site, sex, age and quadratic age. Red dots represent group means, and red vertical lines represent within-group standard deviations of the residuals from the above modeling. P-values shown are for the overall differences across the 3 groups.


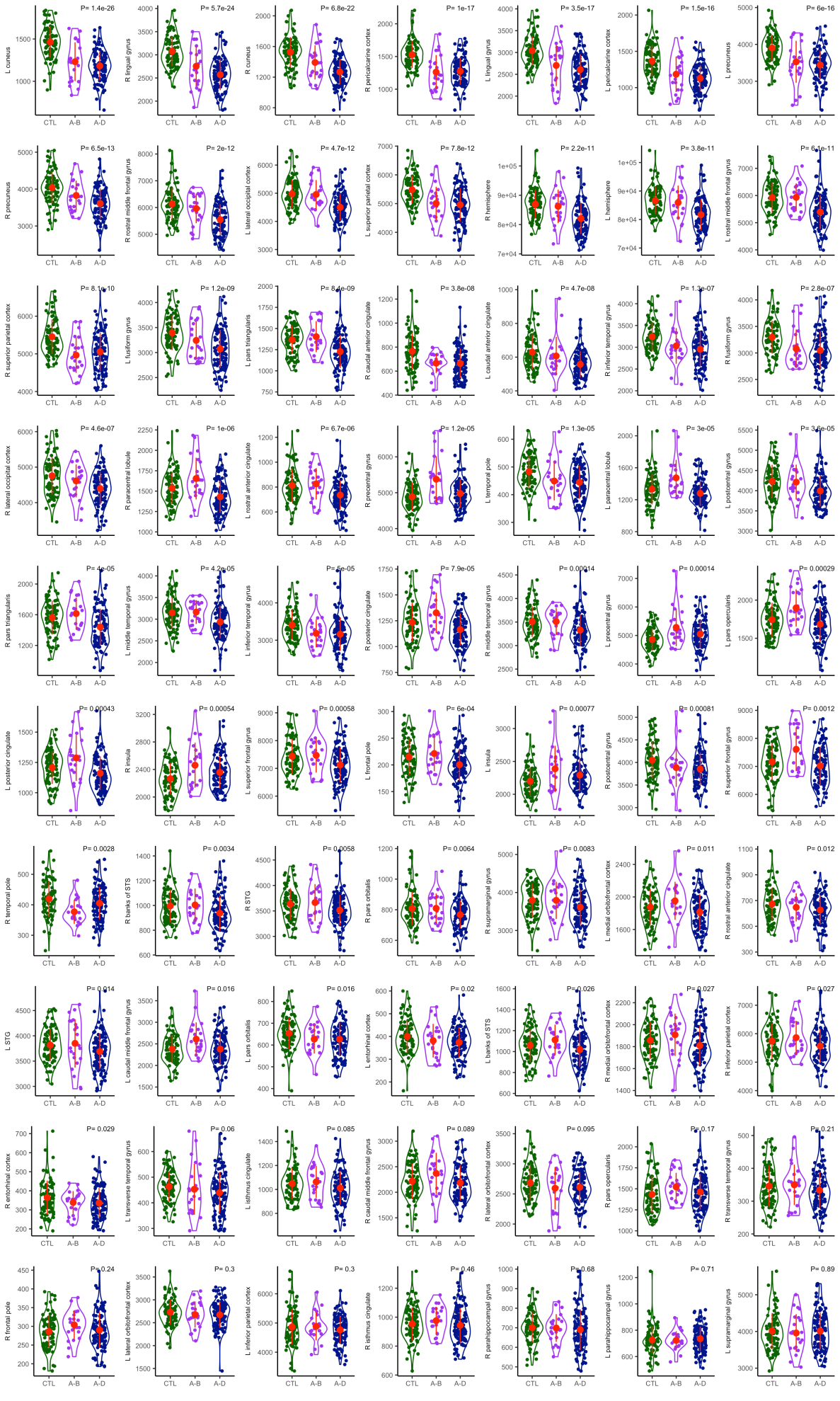


Figure S6b. Scatterplots of Regional Surface Area Differences between A-B Deletion, A-D Deletion and Control Subjects (above). The data-points were adjusted for study site, sex, age, and ICV. Red dots represent group means, and red vertical lines represent within-group standard deviations of the residuals from the above modeling. P-values shown are for the overall differences across the 3 groups.


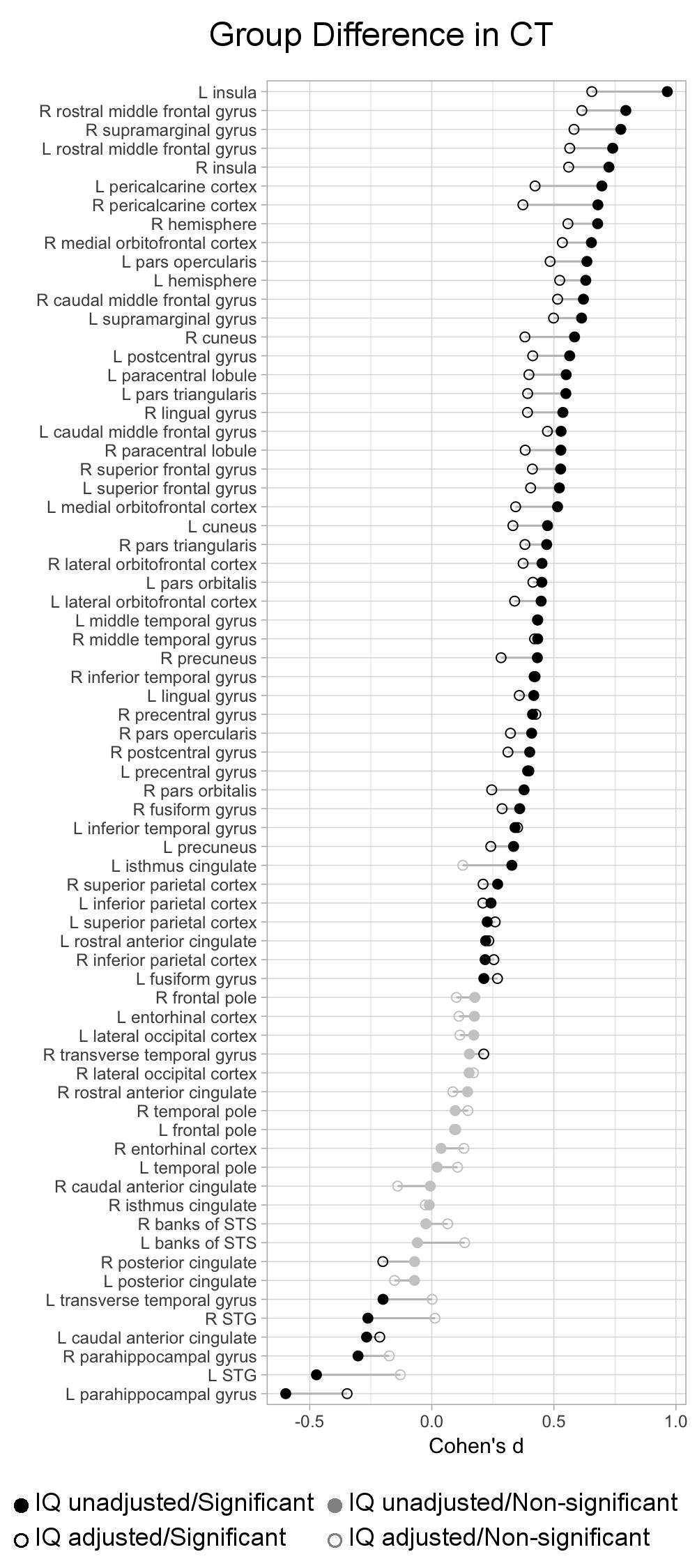


Figure S7a. IQ Adjusted and Unadjusted Group Differences (Cohen’s d) for CT: 22q11DS Cases vs. Controls (above). Effect size values in Cohen’s d were plotted for the group differences in CT between 22q11DS cases and controls, contrasting those from the statistical model that adjusted for IQ with that didn’t adjust for IQ, in order to examine the influence of IQ on CT differences. Solid circles show Cohen’s d values unadjusted for IQ, and hollow circles show values adjusted for IQ. The black color indicates statistical significance after FDR corrections, while the gray color indicates non-significant results. The figure demonstrates that although the absolute effect sizes are reduced when adjusted for IQ, the significance levels and overall pattern of group differences largely remain the same.


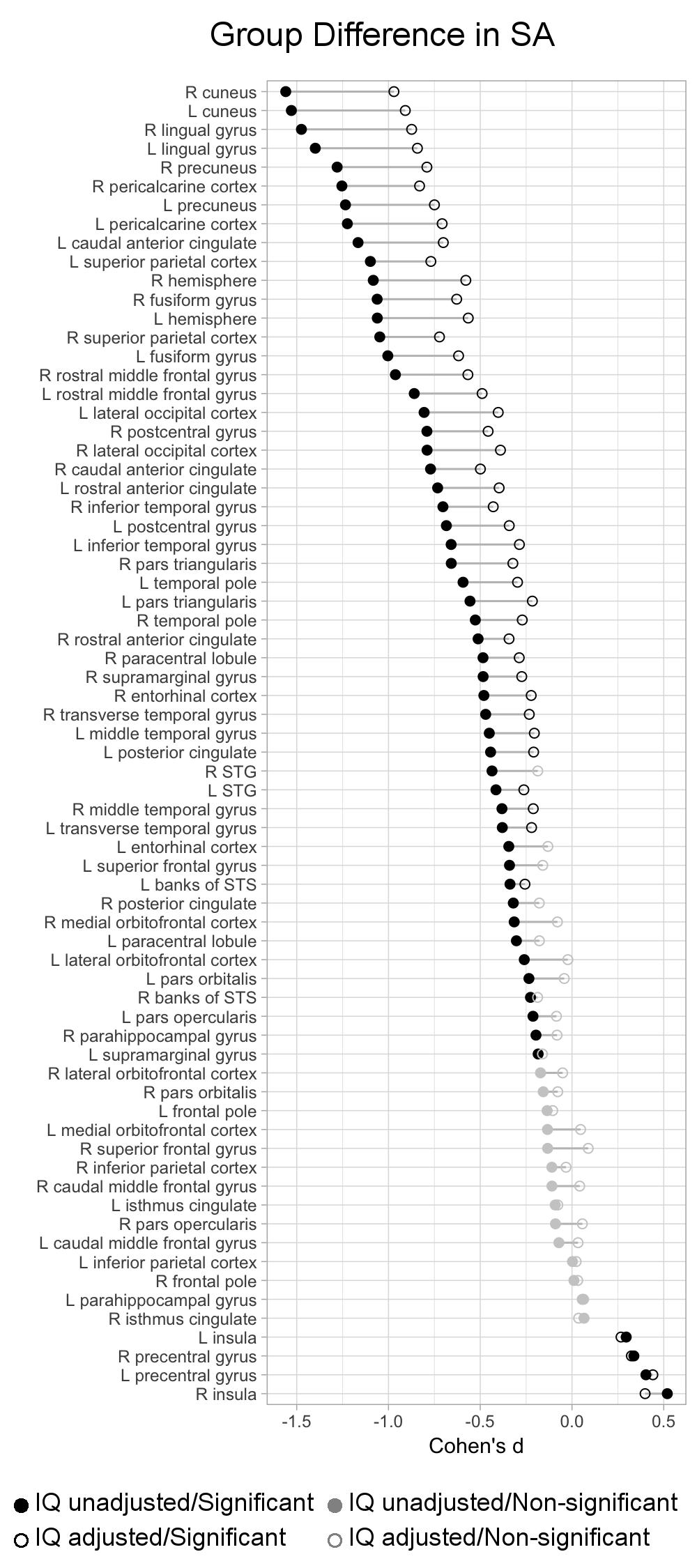


Figure S7b. IQ Adjusted and Unadjusted Group Differences (Cohen’s d) for SA: 22q11DS Cases vs. Controls (above). Effect size values in Cohen’s d were plotted for the group differences in SA between 22q11DS cases and controls, contrasting those from the statistical model that adjusted for IQ with that didn’t adjust for IQ, in order to examine the influence of IQ on SA differences. Solid circles show Cohen’s d values unadjusted for IQ, and hollow circles show values adjusted for IQ. The black color indicates statistical significance after FDR corrections, while the gray color indicates non-significant results. The figure demonstrates that although the absolute effect sizes are reduced when adjusted for IQ, the significance levels and overall pattern of group differences largely remain the same.


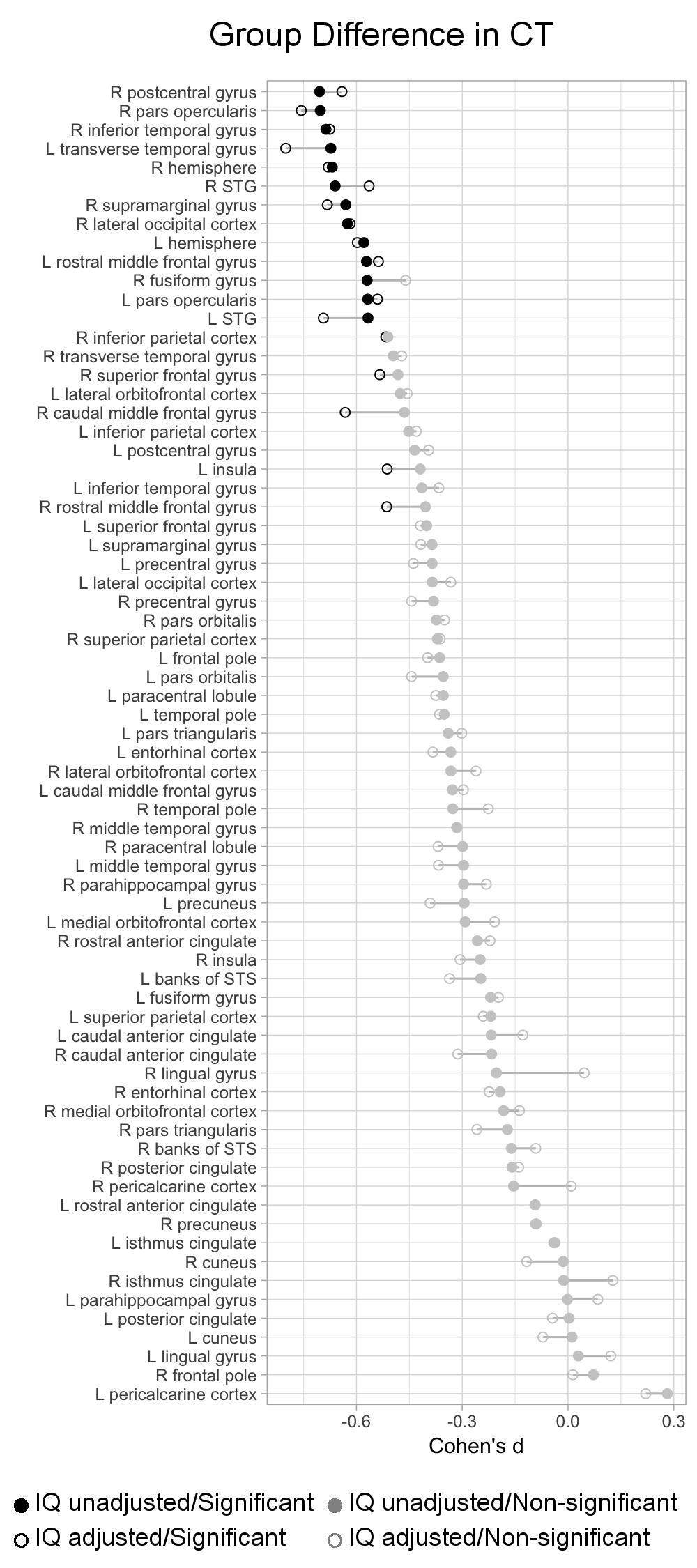


Figure S8. IQ Adjusted and Unadjusted Group Differences (Cohen’s d) for CT: 22q11DS cases with psychosis and without psychosis (above). Effect size values in Cohen’s d were plotted for the group differences in CT between 22q11DS cases with a diagnosis of psychosis and without psychosis, contrasting those from the statistical model that adjusted for IQ with that didn’t adjust for IQ, in order to examine the influence of IQ on CT differences. Solid circles show Cohen’s d values unadjusted for IQ, and hollow circles show values adjusted for IQ. The black color indicates statistical significance after FDR corrections, while the gray color indicates non-significant results. The figure demonstrates that although the absolute effect sizes are slightly altered when adjusted for IQ, the significance levels and overall pattern of group differences largely remain the same.
